# [3+2]-Cycloaddition of Nitrile Imines to Parabanic Acid Derivatives—An Approach to Novel Spiroimidazolidinediones

**DOI:** 10.3390/ijms25010018

**Published:** 2023-12-19

**Authors:** Juliana V. Kuznetsova, Varvara T. Tkachenko, Lada M. Petrovskaya, Maria E. Filkina, Dmitry E. Shybanov, Yuri K. Grishin, Vitaly A. Roznyatovsky, Viktor A. Tafeenko, Anna S. Pestretsova, Vera A. Yakovleva, Vadim S. Pokrovsky, Maxim E. Kukushkin, Elena K. Beloglazkina

**Affiliations:** 1 Department of Chemistry, M.V. Lomonosov Moscow State University, Leninskie Gory, 1-3, 119991 Moscow, Russia; ulenkaiulenka@mail.ru (J.V.K.); varya.tkachenko.99@list.ru (V.T.T.); petrovskayalada@mail.ru (L.M.P.); suuperolen@yandex.ru (M.E.F.); deshyb@mail.ru (D.E.S.); ykgris@mail.ru (Y.K.G.); vit.rozn@nmr.chem.msu.ru (V.A.R.); tafeenko-victor@yandex.ru (V.A.T.); lemmingg@mail.ru (M.E.K.); 2 Blokhin National Medical Research Center of Oncology, Ministry of Health of the Russian Federation, 115478 Moscow, Russia; annaparilla.88@gmail.com (A.S.P.); pokrovskiy-vs@rudn.ru (V.S.P.); 3 Occupational Health Risks Lab, Peoples’ Friendship University of Russia (RUDN University), 117198 Moscow, Russia; 4 Department of Biochemistry, People’s Friendship University of Russia (RUDN University), 117198 Moscow, Russia; yakovleva_va@mail.ru; 5 Research Institute of Molecular and Cellular Medicine, People’s Friendship University of Russia (RUDN University), 117198 Moscow, Russia

**Keywords:** parabanic acids, imines, imidazolones, spiro-compounds, 1,3-dipolar cycloaddition, nitrile imines

## Abstract

Approximately 1,3-Dipolar cycloaddition of imidazolidine derivatives containing exocyclic double bonds is a convenient method of creating spiro-conjugated molecules with promising anticancer activity. In this work, the derivatives of parabanic acid (2-thioxoimidazolidine-4,5-diones and 5-aryliminoimidazolidine-2,4-diones) were first investigated as dipolarophiles in the reactions with nitrile imines. The generation of nitrile imines was carried out either by the addition of tertiary amine to hydrazonoyl chlorides «drop by drop» or using the recently proposed diffusion mixing technique, which led to ~5–15% increases in target compound yields. It was found that the addition of nitrile imines to C=S or C=N exocyclic double bonds led to 1,2,4-thiazolines or triazolines and occurred regioselectively in accordance with the ratio of FMO coefficients of reactants. The yield of the resulting spiro-compound depended on the presence of alkyl substituents in the nitrile imine structure and was significantly decreased in reactions with imines with strong electron donor or electron-withdrawing groups. Some of the obtained compounds showed reasonable in vitro cytotoxicity. IC50 values were calculated for HCT116 (colon cancer) cells using the MTT (3-(4,5-dimethylthiazol-2-yl)-2,5-diphenyltetrazolium bromide) test.

## 1. Introduction

**Figure 1:**
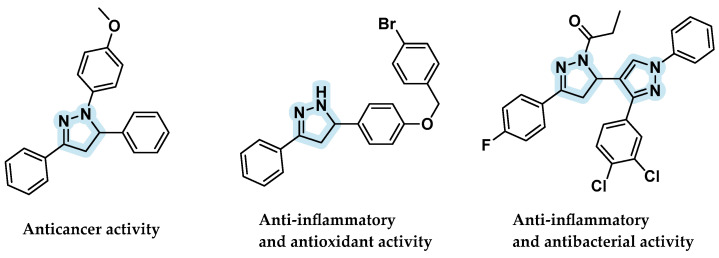
Pyrazolines have different types of biological activity [3,5,6].

Nitrile imines are widely recognized for their high reactivity in 1,3-dipolar cycloaddition reactions, both in carbon-carbon multiple bonds and carbon-heteroatom bonds [1]. The reactions of nitrile imines with asymmetric dipolarophiles usually proceed regio- and chemoselectively [2]. The addition of nitrile imine to the C=C double bond results in the formation of a pyrazoline fragment, which is found in compounds possessing a broad spectrum of biological activities, such as anti-inflammatory [3], antiviral, antimicrobial [4], analgesic, immunosuppressive, antibacterial [5], anticancer [6], antidepressant, and neuroprotective [7] properties (Figure 1). The discovery of these properties has made the reactions of dipolar cycloaddition of nitrile imines a common tool in the synthesis of biologically active molecules and natural compounds [8].

**Figure 2:**
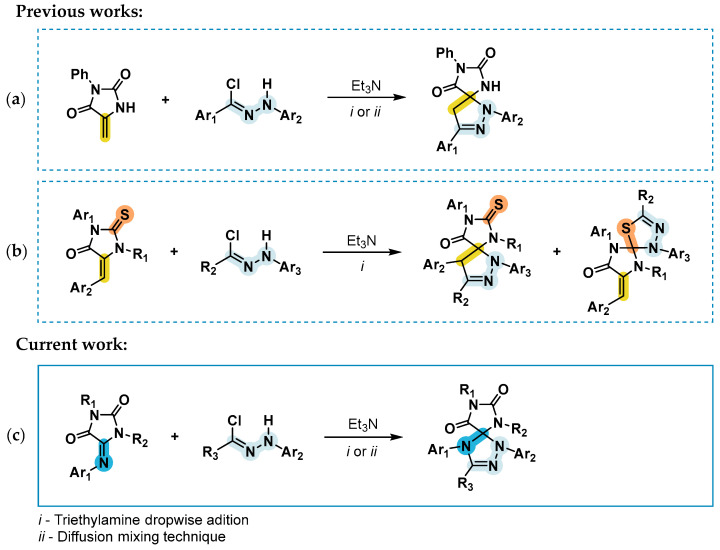
A total of 1,3-dipolar cycloaddition reactions of nitrile imines to (**a**) 5-methylidene hydantoin [11,14]; (**b**) 5-arylidene thiohydantoins [10,13]; and (**c**) 5-arylimino hydantoins (this work).

Hydantoins exhibit a wide range of biological activity, among which antitumor, antibacterial, anticonvulsant, antiepileptic, and antiarrhythmic properties, as well as action as muscle relaxants, can be distinguished [9]. The introduction of methylidene derivatives of hydantoins and thiohydantoins in the 1,3-dipolar cycloaddition reaction enables the preparation of spiro-conjugated compounds containing several heterocyclic pharmacophore fragments in one molecule [10,11,12] (Figure 2a,b). Sterically constrained structure of spiro-conjugated molecules coupled with bioactive fragments suggests the appearance of noticeable cytotoxic properties in the resulting compounds and thereby causes interest not only for organic chemistry but also for medical chemistry. Despite the fact that 5-arylidene(thio)hydantoins have long been known as dipolarophiles [12,13], their analogues containing exocyclic C=N bonds have not been previously investigated in such reactions.

Unfortunately, we did not find any data in the literature on the biological properties of 5-arylidene(thio)hydantoins. However, the recent work of Ribeiro [15] revealed that the replacement of a pyrazoline ring by a triazoline ring in the spirooxindole core altered the antiproliferative profile. Spiropyrazolines showed in general less activity and selectivity towards tested cancer cell lines over the non-cancer-derived cell line than spirotriazolines with the same substitution pattern. Consequently, we assume that the products of the addition of nitrile imines to 5-imino hydantoins studied in this work should also have pronounced cytotoxicity.

There are in the literature a few examples where compounds with exocyclic C=N bonds were used as dipolarophiles. However, the available data demonstrated a good reactivity of the imino group in comparison with the trisubstituted bond in alkenes [16]. In all cases, the addition of nitrile imines to the C=N bond occurs regioselectively with the formation of 1,2,4-triazole [1].

In a recent work by S.H. Ungoren [17], it has been shown that the interaction of N,N-disubstituted parabanic acids with aryliminophosphoranes leads to the formation of 5-imino-substituted hydantoins with a good yield. Among the data available in the literature on the chemical properties of 5-substituted hydantoins in the 1,3-dipolar cycloaddition reactions, we found no examples of the interaction of some 1,3-dipoles and an exocyclic bond C=N of such compounds.

**Scheme 1:**
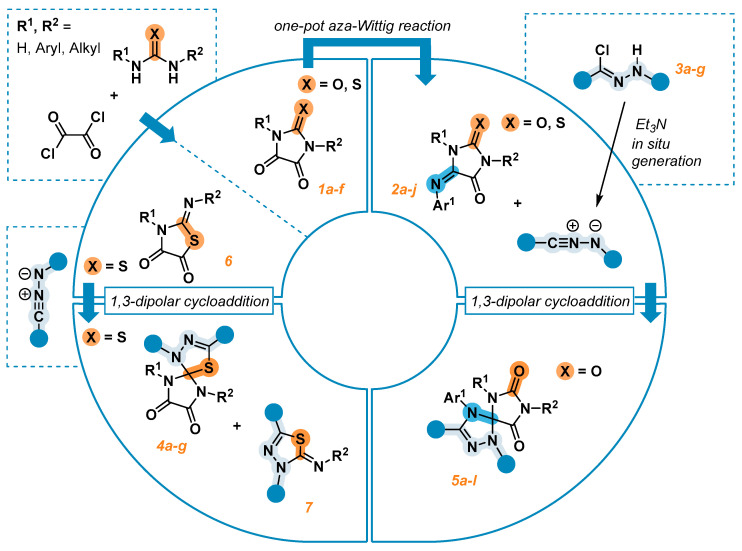
Synthetic approaches to the regioselective 1,3-dipolar cycloaddition reaction of nitrile imines to thioparabanic acids and 5-imino-substituted hydantoins.

Therefore, in this paper, we investigated the reactivity of N- and N,N’-substituted thioparabanic acids in reactions of 1,3-dipolar cycloaddition. In addition, a one-pot method was proposed for the synthesis of 5-arylimino-1,3-diphenylimidazolidine-4-ones, which were further used as dipolarophiles in reactions with nitrile imines. The transformations discussed in this article are summed up in Scheme 1.

## 2. Results and Discussion

### 2.1. Synthesis of the Imidazolidine-4,5-Diones ***1c–f***

**Scheme 2:**
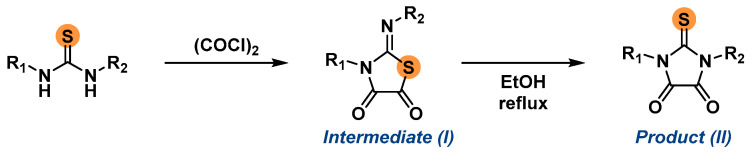
Reaction of disubstituted thiourea and oxalyl chloride.

The preparation of substituted parabanic acids and their sulfur-containing analogues by the reaction of oxalyl chloride and corresponding thioureas was described more than a century ago [18], but for a long time, the structure of the resulting product remained the subject of discussion [18,19,20]. The differences in the obtained results were caused by the possibility of the formation not only of substituted thioparabanic acid **II** but also of intermediate 2-iminothiazolidine-4,5-dione **I**, which in some cases could not be isolated (Scheme 2). At the same time, it was found that the isomerization of intermediate **I** into product **II** occurs quite easily, for example, when boiling in ethanol [20].

**Figure 3:**
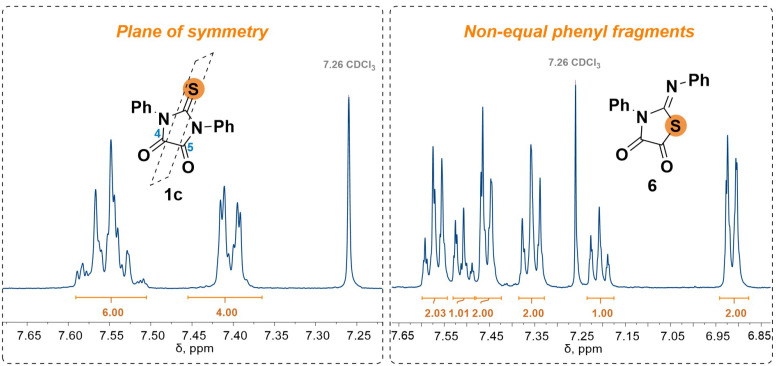
Fragments of ^1^H NMR spectra of 1,3-diphenyl-2-thioxoimidazolidine-4,5-dione **1c** and 3-phenyl-2-(phenylimino)thiazolidine-4,5-dione **6**.

Investigating the reaction of diphenyl thiourea and oxalyl chloride, we found that the formation of a particular of the two isomeric products can be conveniently monitored using NMR spectroscopy. Since 1,3-diphenyl-2-thioxoimidazolidine-4,5-dione **1c** has a plane of symmetry passing through the C=S bond and the middle of the C^4^–C^5^ bond, the protons of aromatic fragments are equivalent and present in the spectrum as two sets of signals. Approximately 3-Phenyl-2-(phenylimino)thiazolidine-4,5-dione **6** does not contain symmetry elements, so the signals of aromatic protons have a more complex multiplicity (Figure 3).

In many publications, data on the formation of isomeric products **I** and **II** (Scheme 2) as a result of the reaction of diphenyl thiourea and oxalyl chloride were presented implicitly [17,21,22], and we could not find a clear methodology that allows us to obtain exclusively isomer **II**. By conducting reactions of various thioureas with oxalyl chloride, we found that a thiazolidine-type product (**I**) was formed very easily. The product was obtained with a high yield after 1–2 h of boiling the reaction mixture in an aprotic solvent with a low boiling point, such as dichloromethane. When boiling the reaction mixture in a more highly boiling solvent, such as acetonitrile, a noticeable amount of isomerized imidazolidine-type product (**II**) was formed in a few hours (about 10% after 2 h). We also found that chromatographic separation of isomers **6** and **1c** is not feasible since 2-imino-thiazolidine-4,5-dione **6** was completely transformed into 2-thioxoimidazolidine-4,5-dione **1c** passing through silica gel.

In contrast to the spectrally distinguishable compounds **1c** and **6**, the determination of the structure of products obtained from oxalyl chloride and non-symmetric thioureas (R_1_ ≠ R_2_) is a more difficult task since the result of the reaction can be both an imidazolidine product **II** and two isomeric thiazolidines **I**. In this regard, imidazolidine-2,4,5-triones **1a–b** and 2-thioxoimidazolidine-4,5-diones **1c–f** were obtained according to two different synthetic strategies. The first ones were obtained by boiling a mixture of oxalyl chloride and urea in DCM, followed by treatment with a solution of sodium bicarbonate to neutralize oxalyl chloride and hydrochloric acid residues. To obtain sulfur-containing products, we used acetonitrile as a solvent and flash chromatography as a purification method, after which only imidazolidine-type products **1c–f** were isolated. The yields of all compounds **1a–f** were good to excellent, with the exception of product **1f**, which contains an unsubstituted N^3^ atom (Table 1).

### 2.2. Synthesis of the 5-Aryliminoimidazolidine-2,4-Diones ***2a–l***

In a recent paper by Ş.H. Ungoren and colleagues [17], it was reported that substituted parabanic acids react with phosphonium ylides to form 5-alkylidene and 5-imino-substituted hydantoins. We modified this technique and carried out the one-pot aza-Wittig reaction without the isolation of intermediate aryliminophosphoranes. Typically, a mixture of arylazide and triphenylphosphine in dry xylene was stirred at a temperature of about 50 °C until nitrogen release stopped (0.5 to 2 h). Imidazoldione **1** was added to the phosphorus ylide formed in situ, after which the mixture was boiled for 2 h. The product was isolated using column chromatography and, if necessary, additionally recrystallized from isopropanol. In all cases, imines **2a–j** were obtained with good yields (Table 2). The resulting product’s C=N double bond configuration was attributed in accordance with the literature data on the structure of similar compounds [17].

**Scheme 3:**
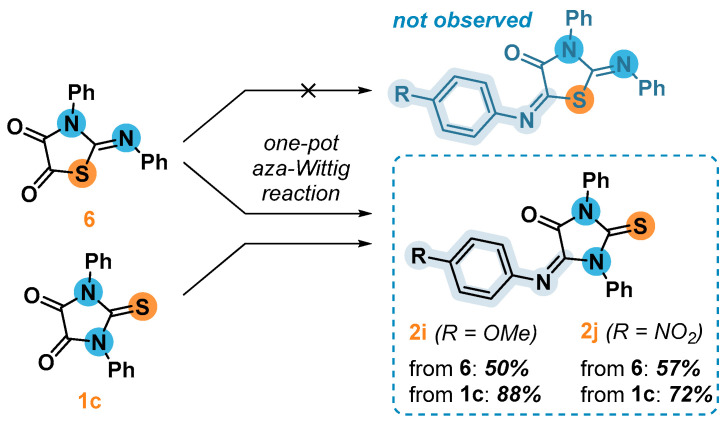
Aza-Wittig reaction of isomers **1c** and **6**.

As previously described [22], Wittig reactions with thiazolidine **6** give the corresponding trisubstituted alkene. Therefore, carrying out a similar aza-reaction to obtain thiazolidine with two C=N bonds simultaneously at positions C^2^ and C^5^ seemed quite real. Contrary to this, we unexpectedly found that compound **6** in the reactions with phosphonium ylides, formed in situ from PPh_3_ and corresponding arylazides, undergoes a one-pot aza-Wittig reaction to furnish the same product as it was formed at the identical conditions from thioparabanic acid **1c**, but with a significantly lower yield (Scheme 3). The presence of an exocyclic C=S bond in compounds **2i** and **2j** was indicated by the appearance of a carbon atom in the thiocarbonyl group signal at ~180 ppm in the ^13^C NMR spectrum. The occurrence of this signal in the crude reaction mixture spectrum suggests isomerization of the thiazolidine cycle into imidazolidine was initiated during the reaction and did not occur during the chromatographic purification of imines **2i** and **2j**.

### 2.3. 1,3-Dipolar Cycloaddition of Nitrile Imines to 2-Thioxoimidazolidine-4,5-Diones ***1c–f*** and 3-Phenyl-2-(Phenylimino)Thiazolidine-4,5-Dione ***6***

**Figure 4:**
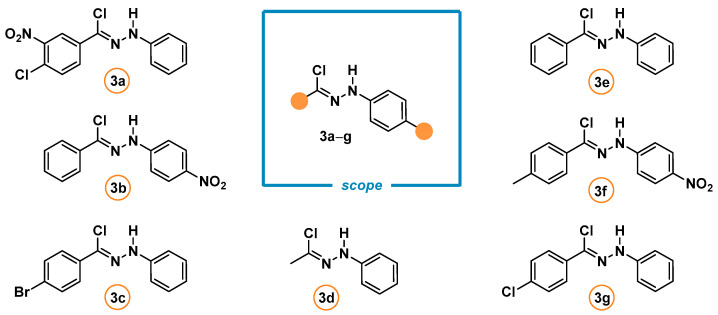
Structures of the hydrazonoyl chlorides **3a–g** used in this study.

Despite the widespread recognition of 1,3-dipolar cycloaddition of thiohydantoins and their 5-methylidene/arylidene substituted analogues for creating spiro-linked compounds with 1,3,4-thiadiazoline [10,11,13] fragments, there are no examples in the literature of 5-oxothiohydantoins (thioparabanic acids) being used as dipolarophiles. Nitrile imines are highly active 1,3-dipoles, so they are usually generated in situ from the corresponding precursors. To obtain nitrile imines, we used hydrazonoyl chlorides **3a–g** (Figure 4), which are readily available by the method [11].

Our laboratory has recently developed the so-called “diffusion reagent mixing technique”, which allows the gradual generation of 1,3-dipoles in solution and effectively suppresses the processes of their unwanted dimerization [14]. In many cases, the diffusion mixing technique makes it possible to significantly increase the yield of the target [3+2]-cycloaddition products and is especially effective in cases of interaction with highly reactive dipoles. For the reactions of 2-thioxoimidazolidine-4,5-diones **1c–f** with nitrile imines, we compared two synthetic approaches, which differed in the way of introducing in the reaction mixture a base generating a dipole from hydrazonoyl chloride **3a–d**. In the first “classical” method, the base solution was added drop by drop to a reaction mixture containing a dipolarophile **1c–f** and a dipole precursor **3a–d**. In the second approach, we used the equipment for the diffusion reagent mixture (see [15] and Supplementary Information), whereby the volatile base spontaneously diffused into a solution of reagents.

Both methods of reagent mixing enabled us to obtain spiro compounds **4a–g** with good yields (Table 3).

**Scheme 4:**
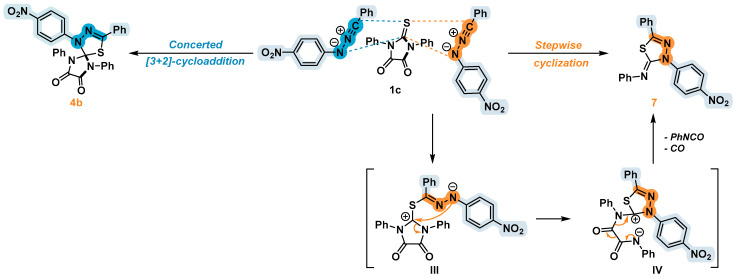
The proposed mechanism of 1,3-diphenyl-2-thioxoimidazolidine-4,5-dione **1c** and nitrile imine interaction.

It was found that the use of the diffusion mixing technique resulted in target product yields comparable to or exceeding the results obtained by adding amine drop by drop. Compound **4c** was obtained with a yield near quantitative using both methods. The only exception was compound **4b**, whose yield when using the diffusion mixing technique was significantly lower due to the formation of a by-product **7** (Scheme 4).

It is known that in several cases [11,23], the interaction of nitrile imines and cyclic dipolarophiles led to the destruction of the initial imidazolidine cycle instead of the creation of a spiro-jointed product. Probably, in our case, as in the above publications, the presence of a strong electron acceptor (nitro group) in the dipole structure stabilizes the zwitterionic intermediate **III**, leading to the formation of the product **7** (Scheme 4). At the same time, the fragmentation of the molecule apparently occurs only when two aromatic fragments are present in the structure of the thioparabanic acid (no formation of a by-product was observed when products **4f** and **4g** were obtained). An analogous influence of the nature of substituents on the tendency to fragmentation was previously observed in the formation of spiro compounds from 5-arylidene thiohydantoins and thiosemicarbazides [24]. The structure of product **7** was proposed based on a match between the spectral data obtained by us and those described in the literature [25].

Optimizing the conditions for the reaction of nitrile imines and 2-thioxoimidazolidine-4,5-diones, we found out that the yield of the target spiro-conjugated product **4a–g** for most substrates was primarily influenced by the electronic properties of the substituents and did not depend much on the solvent selection. The situation dramatically changed when product **7** with a disrupted imidazolidine fragment could be formed as a result of the reaction (Table 4). TLC analysis of the reaction mixture revealed that compound **7** in high-polar solvents was either formed in trace amounts (Entry 2) or was absent from the mixture (Entry 1). Furthermore, the use of slightly less polar acetone or chloroform in the reaction led to the formation of a large amount of by-product **7**, in some cases even exceeding the amount of the target spiro compound **4b** (Entry 4 and 5). The yield of product 4b slightly increased when the reaction mixture cooled (Entry 3), but the significance of this factor was negligible.

**Scheme 5:**
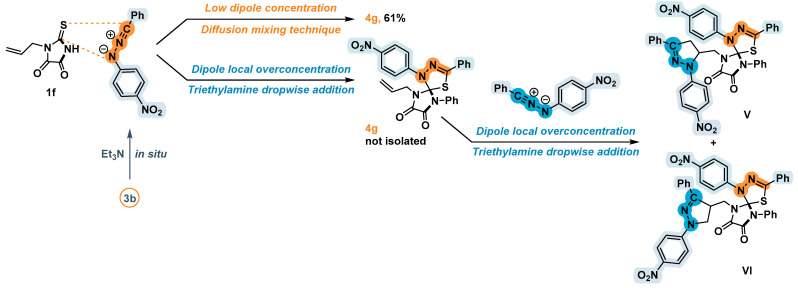
The proposed products of the 1-allyl-2-thioxoimidazolidine-4,5-dione **1f** and hydrazonoyl chloride **3b** reactions.

The monosubstituted double bond –CH=CH_2_ is known to be capable of reacting easily in the 1,3-dipolar cycloaddition, but in most cases, the thiocarbonyl group is a significantly more active dipolarophile than the carbon-carbon double bond [8]. The difference in the reactivity of C=S and C=C groups made it possible to synthesize the compound **4g**, the reaction product of the thioparabanic acid **1f** containing an allyl substituent at the nitrogen atom. By employing the diffusion mixing technique, we managed to obtain the product **4g** in a good yield (Table 3), while carrying out this reaction by adding triethylamine drop by drop led to the formation of a mixture of compounds that could not be qualitatively separated by chromatography. Based on the analysis of the spectral data obtained for this mixture, we assumed that the dropwise addition of a very dilute triethylamine solution to **1f** and hydrazonoyl chloride **3b** led to a local overconcentration of the generated nitrile imine, which was sufficient to attach the second dipole molecule to the already formed spiro compound **4g** (Scheme 5). Presumably, the addition of nitrile imine to the carbon-carbon double bonds of the allyl substituent of the product **4g** occurred analogously to reactions with monosubstituted olefins [1] and led to the formation of two regioisomers **V** and **VI** very similar in their chromatographic properties. In the case of diffusion, the unstable 1,3-dipole was present in the solution at a low concentration sufficient for reaction with only one most active C=X bond.

**Scheme 6:**
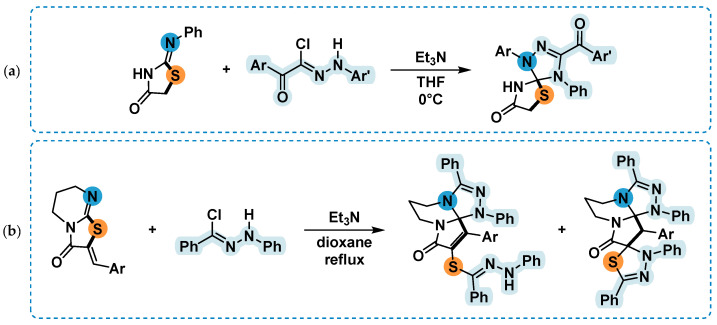
Reactions of nitrilimines with 2-iminothiazolidine-4-one (**a**) and its 5-arylidene derivative (**b**) [26,27].

Earlier, it was reported that the cycloaddition of nitrile imines to the C=N bond of 2-iminothiazolidine-4-ones makes it possible to obtain a spiro-joined product containing the unaffected thiazolidine cycle [26] (Scheme 6a). Meanwhile, the reactions of diphenyl nitrile imine with 5-arylidene-substituted 2-iminothiazolidine-4-one resulted in the formation of a product of the sequential addition of two dipole molecules along both exocyclic double bonds C=N and C=S, which were formed as a result of the rearrangement of the intermediate spiro compound [27] (Scheme 6b).

**Scheme 7:**
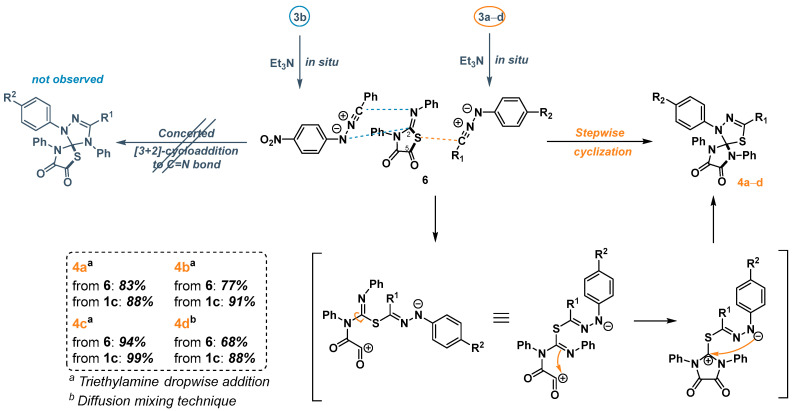
The proposed mechanism of 3-phenyl-2-(phenylimino)thiazolidine-4,5-dione **6** and nitrile imine interaction.

In our study, when compound **6** was introduced into the reaction with nitrile imines, we established that the sulfur atom’s tendency to isomerize to an exocyclic position prevailed over the reactivity of the 2-arylimino fragment. The presence of a second carbonyl group in the structure of compound **6** facilitated a process similar to that occurring during isomerization in **1c**, which was described in [20]. It appears that, unlike the process described in the article [27], interaction with the dipole leads to a cleavage of the S-C^5^ bond instead of the S-C^2^ bond (Scheme 7).

Based on the obtained results, it can be concluded that 2-thioxoimidazolidine-4,5-diones **1c–f** possess the capability to undergo the 1,3-dipolar cycloaddition with nitrile imines obtained in situ from the corresponding hydrazonoyl chlorides **3a–d**. As a result, heterocycles **4a–g** consisting of spiro-conjugated imidazolidine and 1,2,4-thiadiazoline fragments were formed. The nitrile imine generation can be accomplished through both the “classical” method of adding the base drop by drop and the previously proposed diffusion mixing technique. The use of the latter enabled the site-selective attachment of the dipole to the C=S bond of 2-thioxoimidazolidine-4,5-dione **1f** in the presence of a second dipolarophilic double bond in the molecule and the formation of compound **4g**. The formation of imidazolidine cycle fragmentation product **7** was observed when spiro compounds were obtained from 1,3-diphenyl-2-thioxoimidazolidine-4,5-dione **1c** and nitrile imine generated by the diffusion mixing technique. Cycloaddition of nitrile imines to 3-phenyl-2-(phenylimino)thiazolidine-4,5-dione **6** resulted in the same products **4a–d** as were obtained from 1,3-diphenyl-2-thioxoimidazolidine-4,5-dione **1c**.

### 2.4. 1,3-Dipolar Cycloaddition of Nitrile Imines to 5-Arylimino-1,3-Diphenylimidazolidine-2,4,5-Triones

Approximately 5-Arylimino-1,3-diphenylimidazolidine-2,4,5-triones **2b–g** were introduced into reactions with nitrile imines obtained in situ from hydrazonoyl halides **3b–g** (Figure 4). Two alternative techniques were also used to generate dipoles: diffusion mixing and triethylamine dropwise addition (see detailed description in Section 2.3). Spiro compounds **5a–l** were obtained in a moderate-to-high yield, which, however, was lower on average compared to those obtained for compounds **4** (Table 5).

The diffusion mixing technique revealed a higher yield of the spiro compounds than when triethylamine was added drop by drop, except for products **5f** and **5j** obtained from imine **2e** (R_1_ = 4-Me). The yield of the cycloaddition product was highly influenced by the electronic properties of the substituents in the initial imine; the presence of a strong donor or acceptor substituent at the R_1_ position led to a sharp decrease in the yield of the compounds **5b**, **5g–i**. The obtained results indicate that the C=N group of 5-arylimino-1,3-diphenylimidazolidine-2,4-diones is, in most cases, less active than the 2-thiocarbonyl group of compounds **1**. When imines **2h–j** containing a couple of reactive C=S and C=N bonds were introduced into the reactions with hydrazonoyl chlorides, we observed the formation of a complex mixture of products, presumably consisting of spiro compounds obtained as a result of the addition of a dipole along one of the C=S or C=N bonds, as well as the addition products of two dipole molecules along both of these bonds with two possible diastereomers formation.

We also tried to introduce 1,3-dipoles generated from hydrazonoyl chlorides **3** with N-heterocyclic substituents (see Table 5, R_2_ = 2-pyridyl- or 5-(H-methyl-pyrazolinyl)) into reactions with imidazolidine-diones. However, the test reactions showed that under conditions optimized for aryl-substituted reagents, not only is it not possible to carry out prepatively the cycloaddition reactions of the corresponding nitrilylides with the formation of the target spiro-compounds, but even the starting hydrazonoyl chlorides **3** were obtained in low yields. It is possible that it occurs due to the presence of basic nitrogen atoms in the nitrogen-containing heterocycles, which can be protonated by HCl released during the dehydrohalogenation of a starting hydrazonyl halide. The reaction conditions when using heterocyclic derivatives, therefore, should be additionally optimized and will be the subject of our subsequent studies.

**Figure 5:**
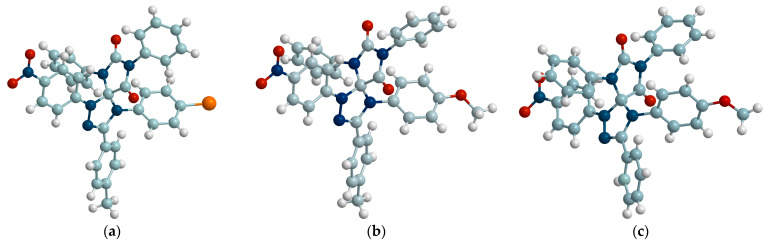
Molecular structures of compounds (**a**) **5c**; (**b**) **5g**; and (**c**) **5i**.

Considering the cycloaddition reactions of nitrile imines from the point of frontier molecular orbital (FMO) theory, they are referred to as dipoles, reactions with which can be controlled both by the interaction between the highest occupied molecular orbital of the dipole (HOMO_dipole_) and the lowest unoccupied molecular orbital of the dipolarophile (LUMO_dipolarophile_) and by the interaction between the LUMO of the dipole and the HOMO of the dipolarophile [28]. Despite the limited quantity of calculated data regarding the reactions of these dipoles with imines, it is widely acknowledged that the regioselectivity of the 1,3-dipolar cycloaddition is governed by the relative disposition of dipole and dipolarophile frontier orbitals [28]. Both with the control of the HOMO_imine_-LUMO_dipole_ gap and with the overlap of the LUMO_dipole_-HOMO_imine_, the reaction leads to the formation of a 1,3,4-triazole fragment [29]. In all the cases examined, we observed a regioselective addition of nitrile imines to the C=N double bond of 5-arylimino-1,3-diphenylimidazolidine-2,4-diones, which corresponds to the theoretical principle regarding the course of such reactions. The structures of compounds **5c**, **5g**, and **5i** were confirmed by X-ray diffraction analysis for the single crystals (Figure 5).

The presence of substituents with pronounced electron-donating or electron-acceptor effects in the dipolarophile structure changes the energy of the frontier molecular orbitals, raising it (if the substituent has donor properties) or decreasing it (acceptor). As a result, one of the ways of overlapping the dipole and the dipolarophile frontier orbitals becomes more preferable [28]. Upon analyzing the correlation between the yield of the resulting spiro compounds and the electronic properties of substituents in aromatic fragments, we found a number of patterns:

- The presence of substituents with strong mesomeric effects in the aromatic ring of imine 2 decreased the yield of the spiro compound, regardless of whether the substituent was a donor or acceptor. For example, the yield decreased in the sequence **5c** (R_1_ = 4-Br) > **5g** (R_1_ = 4-OMe) > **5b** (R_1_ = 4-NO_2_) (Table 5).
- The introduction of a donor substituent into the aromatic fragment at the terminal carbon atom of nitrile imine increased the yield of the cycloaddition product. For example, the yields of the products **5c** (R_1_ = 4-Br, R_2_ = 4-Me-C_6_H_4_) and **5g** (R_1_ = 4-OMe, R_2_ = 4-Me-C_6_H_4_) were higher than those of **5h** (R_1_ = 4-Br, R_2_ = Ph) and **5i** (R_1_ = 4-OMe, R_2_ = Ph), respectively (Table 5).

The side processes of the dipole dimerization, which are typical for the 1,3-dipolar cycloaddition, were suppressed during the diffusion addition of amine by generating a dipole at low concentration and with the addition of a base dropwise due to adding its dilute solution. Therefore, the decrease in the yields of target spiro compounds **5b** and **5g–i** (Table 5) is presumably attributed to the low imine reactivity instead of other factors impeding the dipole addition. In these cases, the conversion of the initial imines remained low even after several days of conducting the reaction, while the yield of the product changed insignificantly with an increase in the amount of hydrazonoyl chlorides or triethylamine added to the reaction.

It also should be noted that when the reacting nitrile imines were stabilized by the single aromatic substituent (compounds **4d** in Table 4, **5k**, and **5l** in Table 5), the difference between the yields of the spiro compound **5** was more significant using different dipole generating techniques than for compounds **4**, where the cycloaddition of nitrile imine occurred to the C=S bond. For instance, the difference in yields of the compound **5k** obtained by diffusion mixing and by triethylamine dropwise addition was nearly twice as large as for the product **4d** and reached almost 30%. This disparity may be caused by the facile dimerization of the dipole obtained from the hydrazonoyl halide **3d**, with a relatively low reactivity of the dipolarophile. For compound **5l**, the difference in yields of target spiro-compounds at different methods of dipole generation was not so significant (16%), apparently due to the greater activity of the C=N bond.

**Figure 6:**
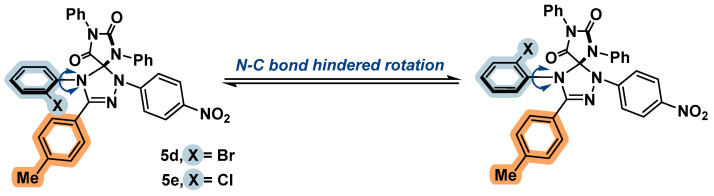
Hindered rotation in compounds **5d** and **5e** is caused by the presence of ortho-substituents in aryl fragments.

The surprising result was that substituents in the aromatic fragment of imine **2** did not have a significant steric effect on the yields of **5c** (R_1_ = 4-Br), **5d** (R_1_ = 2-Br), and **5e** (R_1_ = 2-Cl), which were comparable for both methods of dipole generation (dropwise or diffusion mixing). Despite this, the NMR spectra of the isolated products **5d** and **5e** demonstrated two sets of signals, indicating the formation of two isomers. These isomers in the case of compound **5d** were separated by column chromatography (see Section 3.5), in contrast to the isomers of **5e**, distinguishable by TLC analysis but hardly separable preparatively. It was also demonstrated using NMR ^1^H that in the solution, individual isomers of compound **5d** were gradually transformed into an isomeric mixture. In about a month, they reached an equilibrium ratio that coincided with that that had been previously determined for the reaction mixture. If necessary, this isomer mixture may be repeatedly separated. Based on [30], the observed phenomena can be explained by the atropoisomerism conferred by the repulsive interactions of ortho-substituent R_1_ (the halogen atom) and the aromatic substituent R_2_ at the neighboring nitrogen atom of the triazoline cycle (Figure 6).

In conclusion, it is worth noting that the 1,3-dipolar cycloaddition of nitrile imines to compounds **2** containing an exocyclic bond C=N resulted in the formation of a heterocyclic product **5** with the jointed imidazolidine and 1,2,4-triazoline fragments. The yields of the products **5** were generally good to excellent, except for those obtained from imines **2f** and **2g**, whose substituents had pronounced electron-donating or electron-acceptor properties. The formation of complex mixtures comprising the products of the addition of 1 or 2 nitrile imine molecules was observed for the sulfur-containing dipolarophiles **2h–j**. The structures of compounds **5c**, **5g**, and **5i** were confirmed by X-ray diffraction analysis. In the reactions of nitrile imine formed from hydrazonoyl halide **3e** and dipolarophiles **2b** and **2d** containing ortho-substituents in arylimino fragments, products **5d** and **5e** were obtained as a mixture of atropoisomers.

### 2.5. Synthesis and 1,3-Dipolar Cycloaddition Reactions of the Imines ***2k*** и ***2l*** Obtained from Unsimmetrical Ureas

**Scheme 8:**
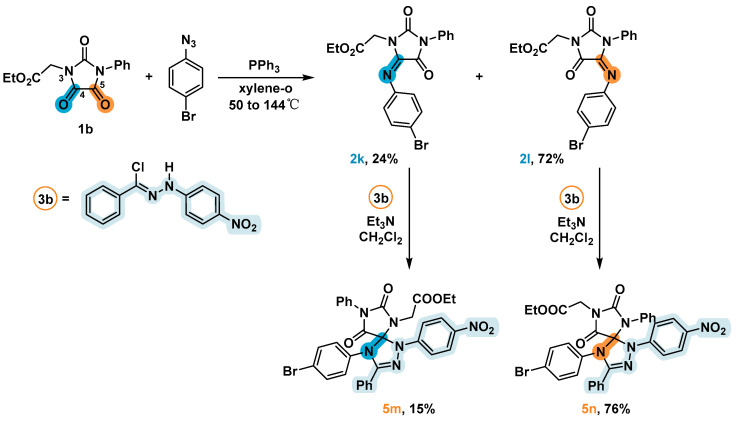
Synthesis of **5m** and **5n**. Isolated yields are presented.

Trying to obtain imine from non-symmetrically substituted imidazoletrione **1b**, we found that both neighboring C=O groups may undergo the reaction, thus giving a mixture of products (Scheme 8). In contrast to the chemoselective aza-Wittig reaction of the carbonyl group at N-Alkyl claimed in [17], in our case, the major product contained an imino group in a position adjacent to N-Ph. The isomeric products **2k** and **2l** had almost identical chromatographic properties and were indistinguishable for TLC analysis at CHCl_3_ elution (this eluent was used in [17]). But the difference between **2k** and **2l** retention factors became noticeable in less polar systems (for example, hexane-EtOAc or hexane-DCM) and was maximum in the hexane-Et_2_O system that we used for separation (see Section 3.3). Since X-ray diffraction analysis of **2k** and **2l** turned out to be unrealizable, a structural organization of the imines was proposed based on the structures of the spiro compounds **5m** and **5n** obtained by their reaction with hydrazonoyl chloride **3b**. The aza-Wittig reaction chemoselectivity observed for substrate **1b** can be attributed to partial delocalization of the lone pair of electrons on nitrogen by an aromatic fragment, which enhanced the carbonyl activity of the C^5^ atom nearby. The ratio of the isolated products (**2k**:**2l** ≈ 1:3) corresponded with the ratio of isomers estimated by the ^1^H NMR spectrum of the aliquot taken from the reaction mixtures before separation.

Compounds **2k** and **2l** were further reacted with hydrazonoyl chloride **3b** to form corresponding products **5m** and **5n** (triethylamine was added dropwise), the structure of which was established on the basis of NMR spectra data. In the ^1^H NMR spectrum of the compound **5m**, the signals of the CH_2_-group protons at the nitrogen atom of the imidazolidine cycle are presented as two doublets at 4.32 and 4.10 ppm with a coupling constant value of 17.6 Hz (see Supplemental Information), which may be due to the difference in the local surroundings of these protons, which are located near the spiro connection. The aliphatic region of the compound **5n** ^1^H NMR spectrum was almost identical to the original imine **2l**, which indicates the remoteness of this substituent from other fragments of the molecule.

The activity of compounds **2k** and **2l** in 1,3-dipolar cycloaddition reactions may be generalized as follows: On the one hand, at the identical preparative procedures, the yield of **5n** was significantly higher than that obtained in the reaction of the same hydrazonoyl chloride (**3b**) and imine containing only aromatic substituents (Yield_drop_ (**5h**) = 50% vs. 76% for **5n**). On the other hand, the compound **5m** was obtained with a low yield of 15%. At the same time, we observed a very low conversion of imine **2k**, which was isolated unchanged at the end of the reaction.

The replacement of phenyl by the -CH_2_COOEt group in imine **2c** led to an increase in the nitrogen atom N^3^ lone electron pair delocalization between neighboring carbon atoms C^2^ and C^4^ in both compounds (**2k** and **2l**). Consequently, if the fragment C=N is located at C^4^ (imine **2k**), the electron density on this atom increases significantly more than in compound **2l**, where the arylimino group is located at atom C^5^ (due to two factors: first, this atom is more distant from N^3^, and second, N^3^ turns out to be surrounded by two C=O groups, delocalizing its electron density). This small increase in the electron density of the C=N bond could contribute to the optimal overlap between the MO imine and the dipole, thereby dramatically raising the yield of the cycloaddition product.

### 2.6. Biological Evaluation of Selected Spiro-Compounds

The cytotoxicity of some spiro derivatives of both structural types **4** and **5** was evaluated using the MTT (3-(4,5-dimethylthiazol-2-yl)-2,5-diphenyltetrazolium bromide) test in the human colorectal carcinoma cell line HCT116 (Table 6). Overall, the selected compounds demonstrated moderate cytotoxic activity with IC_50_ values in the range of ~25–45 µM.

The obtained cytotoxicity results allow us to draw some preliminary conclusions about the effect of substituents in aromatic fragments on the activity of compounds. Firstly, type **4** compounds with a NO_2_-group in one of the aromatic fragments showed slightly better results (compounds **4a**, **4b**, and **4f**) compared to **4e**. On the other hand, compound **5j**, which does not contain NO_2_-groups, showed lower IC_50_ values compared to **5c** and **5f**. When comparing the IC_50_ values for compounds **4b** and **4f,** it can be noticed that the combination of a substituted aromatic fragment and a CH_2_COOEt group at nitrogen atoms is somewhat more preferable than the presence of two phenyls. It can also be pointed out that the presence of a halogen atom in the R_1_ position of the compound **5c** led to a slight increase in cytotoxicity compared to **5f** (R_1_ = CH_3_). As can be seen from the IC_50_ measurements, the cytotoxicity of the presented compounds belongs to the region of micromolar concentrations, which is a good but insufficient value compared to the known lead compounds [12,30]. This result may be explained by the high lipophilicity of compounds containing 3–5 aromatic fragments within a relatively small volume of the molecule.

## 3. Materials and Methods

### 3.1. General Information

The reagents were purchased from commercial sources and used without further purification. All solvents used were purified and dehydrated, as described in [31]. Reactions were checked by thin layer chromatography (TLC) analysis using silica plates with a fluorescent indicator (254 nm) and visualized with a UV lamp.

The nuclear magnetic experiments were recorded using two different spectrometers, BrukerAvance (Bruker Optik GmbH, Ettlingen, Germany) and Agilent 400-MR (Agilent Technologies, Santa Clara, CA, US), operating at 400 MHz for ^1^H and 100 MHz for ^13^C nuclei. Chemical shifts were reported in delta (δ) units (ppm) relative to the residual peak of solvents (ref: CDCl3/DMSO-d6, ^1^H: 7.26/2.50 ppm; ^13^C: 77.16/39.52 ppm) and coupling constants (*J*) in Hz.

The IR spectra were recorded using a Thermo Nicolet iS5 FT-IR Spectrometer (Thermo Electron Scientific Instruments LLC, Madison, WI, USA) with an ATR module.

Electrospray ionization high-resolution mass spectra were recorded in positive ion mode on a TripleTOF 5600+ quadrupole time-of-flight mass spectrometer (ABSciex, Concord, Vaughan, ON, Canada) equipped with a DuoSpray ion source. The following MS parameters were applied: capillary voltage 5.5 kV; nebulizing and curtain gas pressures—15 and 25 psi; respectively; ion source temperature—ambient; declustering potential 20 V; *m*/*z* range 100–1200. Elemental compositions of the detected ions were determined based on accurate masses and isotopic distributions using Formula Finder software (ABSciex, Concord, ON, Canada). The maximum allowed deviation of the experimental molecular mass from the calculated one was 5 ppm.

The X-ray data were collected via the STOE diffractometer Pilatus100K detector (DECTRIS AG, Baden, Switzerland), Cu Kα (1.54086Å) radiation, and rotation method mode. STOE X-AREA software was used for cell refinement and data reduction. Data collection and image processing were performed with X-Area 1.67 (STOE & Cie GmbH, Darmstadt, Germany, 2013). Intensity data were scaled with LANA (part of X-Area) in order to minimize differences in intensities of symmetry-equivalent reflections (multi-scan method). CCDC 2310170 JK269 (compound **5i**), CCDC 2310171 JK314-2 (compound **5g**), and CCDC 2310172MB-13 (compound **5c**) contain the supplementary crystallographic data for this paper. The data can be obtained free of charge from the Cambridge Crystallographic Data Centre via the www.ccdc.cam.ac.uk/data_request/cif (accessed on 17 December 2023) website.

### 3.2. General Procedure for the Synthesis of the Imidazolidine-3,4,5-Triones ***1a–b*** and 3-phenyl-2-(Phenylimino) Thiazolidine-4,5-Dione ***6***

Oxalyl chloride (1.3 eq) was added dropwise to a stirring solution of urea or thiourea (1 eq) in DCM (0.1 M). The resulting mixture was heated at reflux for 2 h, then cooled to room temperature and washed with brine. The aqueous phase was separated and washed twice with small portions of DCM. Combined organic extracts were dried over anhydrous Na_2_SO_4_ and evaporated at reduced pressure to give a solid precipitate of the product.

1,3-Diphenylimidazolidine-2,4,5-trione (**1a**) was obtained from 1,3-diphenylurea (2.12 g, 10 mmol) and oxalyl chloride (1.65 g, 13 mmol) as a white solid (2.64 g, 99%). ^1^H NMR (400 MHz, CDCl_3_) δ 7.57–7.52 (m, 4H, Ar), 7.51–7.45 (m, 6H, Ar).

Ethyl 2-(2,4,5-trioxo-3-phenylimidazolidin-1-yl)acetate (**1b**) was obtained from ethyl (phenylcarbamoyl)glycinate (1.11 g, 5 mmol) and oxalyl chloride (0.83 g, 6.5 mmol) as a white solid (1.37 g, 99%). ^1^H NMR (400 MHz, CDCl_3_) δ 7.55–7.49 (m, 2H, Ar), 7.48–7.41 (m, 3H, Ar), 4.49 (s, 2H, NCH_2_), 4.28 (q, *J* = 7.1 Hz, 2H, COOCH_2_), 1.32 (t, *J* = 7.1 Hz, 3H, CH_3_). ^13^C NMR (101 MHz, CDCl_3_) δ 166.0, 155.7, 155.3, 152.2, 129.8, 129.6, 129.3, 125.8, 62.7, 40.0, 14.2. HRMS (ESI): calcd for C_13_H_12_N_2_O_5_ (M+Na)^+^ 299.0638, found 299.0637.

3-Phenyl-2-(phenylimino)thiazolidine-4,5-dione (**6**) was obtained from 1,3-diphenylthiourea (2.28 g, 10 mmol) and oxalyl chloride (1.65 g, 13 mmol) as a yellow solid (2.80 g, 99%). ^1^H NMR (400 MHz, CDCl_3_) δ 7.60–7.53 (m, 2H, Ar), 7.53–7.48 (m, 1H, Ar), 7.48–7.42 (m, 2H, Ar), 7.39–7.32 (m, 2H, Ar), 7.23–7.18 (m, 1H, Ar), 6.94–6.89 (m, 2H, Ar)

### 3.3. General Procedure for the Synthesis of the 2-Thioxoimidazolidine-4,5-Diones ***1c–f***

Oxalyl chloride (1.3 eq) was added dropwise to a stirring solution of thiourea (1 eq) in acetonitrile (0.1 M). The resulting mixture was heated at reflux for 6 h. The solvent was removed at reduced pressure, and the residue was purified by flash column chromatography on silica gel using CHCl_3_ as eluent.

1,3-Diphenyl-2-thioxoimidazolidine-4,5-dione (**1c**) was obtained from 1,3-diphenylthiourea (2.28 g, 10 mmol) and oxalyl chloride (1.65 g, 13 mmol) as an orange solid (2.68 g, 95%). ^1^H NMR (400 MHz, CDCl_3_):δ 7.59–7.50 (m, 6H, Ar), 7.42–7.37 (m, 4H, Ar).

Ethyl 2-(3-(4-ethoxyphenyl)-4,5-dioxo-2-thioxoimidazolidin-1-yl)acetate (**1d**) was obtained from ethyl ((4-ethoxyphenyl)carbamothioyl)glycinate (0.07 g, 0.25 mmol) and oxalyl chloride (0.04 g, 0.33 mmol) as beige solid (0.08 g, 93%). ^1^H NMR (400 MHz, CDCl_3_) δ 7.25–7.20 (m, 2H, Ar), 7.02–6.97 (m, 2H, Ar), 4.76 (s, 2H, NCH_2_), 4.27 (q, *J* = 7.1 Hz, 2H, COOCH_2_CH_3_), 4.07 (q, *J* = 7.0 Hz, 2H, OCH_2_CH_3_), 1.44 (t, *J* = 7.0 Hz, 3H, OCH_2_CH_3_), 1.31 (t, *J* = 7.2 Hz, 3H, COOCH_2_CH_3_). ^13^C NMR (101 MHz, CDCl_3_) δ 180.0, 165.9, 160.0, 154.8, 154.4, 129.2, 123.7, 115.3, 63.9, 62.5, 42.7, 14.9, 14.2. HRMS (ESI): calcd for C_15_H_16_N_2_O_5_S (M+Na)^+^ 359.0672, found 359.0675.

Ethyl 2-(3-(4-methoxyphenyl)-4,5-dioxo-2-thioxoimidazolidin-1-yl)acetate (**1e**) was obtained from ethyl ((4-methoxyphenyl)carbamothioyl)glycinate (0.11 g, 0.40 mmol) and oxalyl chloride (0.07 g, 0.52 mmol) as beige solid (0.10 g, 77%). ^1^H NMR (400 MHz, CDCl_3_) δ 7.26–7.22 (m, 2H), 7.04–6.99 (m, 2H), 4.77 (s, 2H, NCH_2_), 4.27 (q, *J* = 7.1 Hz, 2H, COOCH_2_CH_3_), 3.85 (s, 3H, OCH_3_), 1.31 (t, *J* = 7.1 Hz, 3H, COOCH_2_CH_3_). ^13^C NMR (101 MHz, CDCl_3_) δ 179.9, 165.9, 160.6, 154.8, 154.4, 129.2, 123.9, 114.9, 62.6, 55.7, 42.7, 14.2. HRMS (ESI): calcd for C_14_H_14_N_2_O_5_S (2M+Na)^+^ 667.1139, found 667.1138.

1-Allyl-2-thioxoimidazolidine-4,5-dione (**1f**) was obtained from 1-allylthiourea (2.42 g, 21 mmol) and oxalyl chloride (3.42 g, 27 mmol) as a yellowish solid (1.67 g, 47%). ^1^H NMR (400 MHz, CDCl_3_) δ 9.11 (br.s., 1H, NH), 5.83 (ddt, *J* = 16.4, 10.1, 6.0 Hz, 1H, CH), 5.34–5.27 (m, 2H, =CH_2_), 4.54 (d, *J* = 6.0 Hz, 2H, NCH_2_). ^13^C NMR (101 MHz, DMSO) δ 178.0, 155.7, 155.0, 129.3, 120.2, 43.7. HRMS (ESI): calcd for C_6_H_6_N_2_O_2_S (M+Na)^+^ 193.0042, found 193.0042.

### 3.4. General Procedure for the Synthesis of the 5-Aryliminoimidazolidine-2,4-Diones ***2a–l***

Triphenylphosphine (1.1 eq) and arylazide (1.1 eq) solutions in xylene (0.1 M) were stirred at 50℃ for 0.5–2 h (until nitrogen release became visually undetectable) and charged with imidazolidine-4,5-dione **1a–c** (1 eq). The reaction mixture was refluxed for 6 h and then left for 16 h with stirring without additional heating. The solvent was removed in vacuo, and the resulting oily mass was purified by flash chromatography with CHCl_3_ as eluent. Compounds **2a–j** were additionally recrystallized from isopropyl alcohol. Compounds **2k** and **2l** were isolated by column chromatography with a hexane-Et_2_O system as eluent (gradient elution of 80/20 to 55/45).

(*E*)-5-((4-Chlorophenyl)imino)-1,3-diphenylimidazolidine-2,4-dione (**2a**) was obtained from **1a** (0.10 g, 0.4 mmol), 1-azido-4-chlorobenzene (0.07 g, 0.44 mmol) and PPh_3_ (0.12 g, 0.44 mmol) as orange solid (0.12 g, 79%). ^1^H NMR (400 MHz, CDCl_3_) δ 7.57–7.51 (m, 4H, Ar), 7.50–7.40 (m, 6H, Ar), 7.28 (d, *J* = 8.5 Hz, 2H, Ar), 6.94 (d, *J* = 8.5 Hz, 2H, Ar). ^13^C NMR (101 MHz, CDCl_3_) δ 153.3, 152.1, 144.6, 131.4, 131.0, 130.4, 130.2, 129.6, 129.4, 129.3, 128.9, 128.9, 128.8, 127.2, 126.0, 125.9, 121.5. HRMS (ESI): calcd for C_21_H_14_ClN_3_O_2_ (M+H)^+^ 376.0847, found 376.0852.

(*E*)-5-((2-Chlorophenyl)imino)-1,3-diphenylimidazolidine-2,4-dione (**2b**) was obtained from **1a** (0.40 g, 1.5 mmol), 1-azido-2-chlorobenzene (0.25 g, 1.65 mmol) and PPh_3_ (0.43 g, 1.65 mmol) as yellow solid (0.34 g, 60%). ^1^H NMR (400 MHz, CDCl_3_) δ 7.66 (d, *J* = 7.9 Hz, 2H, Ar), 7.56 (t, *J* = 7.7 Hz, 2H, Ar), 7.47 (d, *J* = 4.2 Hz, 5H, Ar), 7.40 (d, *J* = 8.2 Hz, 2H, Ar), 7.21 (t, *J* = 7.6 Hz, 1H, Ar), 7.06 (t, *J* = 7.6 Hz, 1H, Ar), 6.95 (d, *J* = 8.0 Hz, 1H, Ar). ^13^C NMR (101 MHz, CDCl_3_) δ 153.2, 152.1, 144.0, 141.5, 131.3, 130.2, 129.6, 129.4, 129.3, 128.9, 127.2, 127.2, 125.9, 125.4, 120.6. HRMS (ESI): calcd for C_21_H_14_ClN_3_O_2_ (2M+Na)^+^ 773.1441, found 773.1443.

(*E*)-5-((4-Bromophenyl)imino)-1,3-diphenylimidazolidine-2,4-dione (**2c**) was obtained from **1a** (0.39 g, 1.45 mmol), 1-azido-4-bromobenzene (0.32 g, 1.60 mmol), and PPh_3_ (0.42 g, 1.60 mmol) as yellow solids (0.37 g, 60%). ^1^H NMR (400 MHz, CDCl_3_) δ 7.57–7.53 (m, 4H, Ar), 7.51–7.45 (m, 5H, Ar), 7.42 (d, *J* = 8.3 Hz, 3H, Ar), 6.87 (d, *J* = 8.2 Hz, 2H, Ar). ^13^C NMR (101 MHz, CDCl_3_) δ 153.2, 152.0, 145.1, 139.9, 131.7, 131.4, 130.2, 129.4, 129.3, 128.9, 128.9, 127.2, 125.9, 121.8, 118.1. HRMS (ESI): calcd for C_21_H_14_BrN_3_O_2_ (M+H)^+^ 420.0342, found 420.0344.

(*E*)-5-((2-Bromophenyl)imino)-1,3-diphenylimidazolidine-2,4-dione (**2d**) was obtained from **1a** (0.19 g, 0.71 mmol), 1-azido-2-bromobenzene (0.15 g, 0.78 mmol) and PPh_3_ (0.20 g, 0.78 mmol) as yellow solids (0.23 g, 76%). ^1^H NMR (400 MHz, CDCl_3_) δ 7.73 (d, *J* = 7.9 Hz, 2H, Ar), 7.63 (d, *J* = 8.0 Hz, 1H, Ar), 7.58 (t, *J* = 7.8 Hz, 2H, Ar), 7.53–7.45 (m, 5H, Ar), 7.43–7.39 (m, 1H, Ar), 7.29 (t, *J* = 7.6 Hz, 1H, Ar), 7.06–6.97 (m, 2H, Ar). ^13^C NMR (101 MHz, CDCl_3_) δ 153.0, 145.3, 132.7, 131.3, 130.2, 129.4, 129.3, 128.9, 127.8, 127.2, 125.9, 125.7, 120.4, 114.1. HRMS (ESI): calcd for C_21_H_14_BrN_3_O_2_ (M+Na)^+^ 442.0162, found 442.0167.

(*E*)-1,3-Diphenyl-5-(p-tolylimino)imidazolidine-2,4-dione (**2e**) was obtained from **1a** (0.27 g, 1.00 mmol), 1-azido-4-methylbenzene (0.15 g, 1.10 mmol) and PPh_3_ (0.29 g, 1.10 mmol) as a light orange solid (0.35 g, 84%). ^1^H NMR (400 MHz, CDCl_3_) δ 7.61–7.52 (m, 4H, Ar), 7.52–7.44 (m, 5H, Ar), 7.43–7.38 (m, 1H, Ar), 7.13 (d, *J* = 7.9 Hz, 2H, Ar), 6.92 (d, *J* = 7.9 Hz, 2H, Ar), 2.33 (s, 3H, CH_3_). ^13^C NMR (101 MHz, CDCl_3_) δ 153.2, 143.4, 139.4, 134.8, 130.4, 129.3, 129.2, 128.8, 128.8, 128.7, 127.3, 126.1, 120.2, 120.1, 120.1, 21.1. HRMS (ESI): calcd for C_22_H_17_N_3_O_2_ (M+H)^+^ 356,1394, found 356.1396.

(*E*)-5-((4-Methoxyphenyl)imino)-1,3-diphenylimidazolidine-2,4-dione (**2f**) was obtained from **1a** (0.53 g, 2.00 mmol), 1-azido-4-methoxybenzene (0.33 g, 2.20 mmol), and PPh_3_ (0.58 g, 2.20 mmol) as orange solid (0.48 g, 65%). ^1^H NMR (400 MHz, DMSO-d6) δ 7.58–7.54 (m, 4H, Ar), 7.52 (d, *J* = 7.6 Hz, 2H, Ar), 7.48–7.44 (m, 4H, Ar), 7.02 (d, *J* = 8.7 Hz, 2H, Ar), 6.84 (d, *J* = 8.8 Hz, 2H, Ar), 3.72 (s, 3H, OCH_3_). ^13^C NMR (101 MHz, DMSO-d6) δ 156.4, 153.9, 152.4, 140.7, 139.0, 132.5, 131.0, 129.0, 128.9, 128.5, 128.2, 127.9, 127.0, 122.3, 113.5, 55.2. HRMS (ESI): calcd for C_22_H_17_N_3_O_3_ (M+H)^+^ 372.1343, found 372.1347.

(*E*)-5-((4-Nitrophenyl)imino)-1,3-diphenylimidazolidine-2,4-dione (**2g**) was obtained from **1a** (0.53 g, 2.00 mmol), 1-azido-4-nitrobenzene (0.36 g, 2.20 mmol) and PPh_3_ (0.58 g, 2.20 mmol) as a light yellow solid (0.58 g, 75%). ^1^H NMR (400 MHz, CDCl_3_) δ 8.19 (s, 2H, Ar), 7.57 (m, 3H, Ar), 7.52–7.38 (m, 7H, Ar), 7.03 (s, 2H, Ar). ^13^C NMR (101 MHz, CDCl_3_) δ 153.4, 152.8, 152.0, 144.5, 140.3, 131.1, 130.0, 129.5, 129.4, 129.2, 129.1, 127.1, 125.8, 124.8, 120.2. HRMS (ESI): calcd for C_21_H_14_N_4_O_4_ (M+H)^+^ 409.0907, found 409.0911.

(*E*)-5-((4-bromophenyl)imino)-1,3-diphenyl-2-thioxoimidazolidin-4-one (**2h**) was obtained from **1c** (0.50 g, 1.77 mmol), 1-azido-4-bromobenzene (0.39 g, 1.94 mmol), and PPh_3_ (0.51 g, 1.94 mmol) as yellow solid (0.64 g, 82%). ^1^H NMR (400 MHz, CDCl_3_) δ 7.60–7.55 (m, 2H, Ar), 7.50 (q, *J* = 9.2, 8.3 Hz, 6H, Ar), 7.43–7.36 (m, 4H, Ar), 6.91 (d, *J* = 8.3 Hz, 2H, Ar). ^13^C NMR (101 MHz, CDCl_3_) δ 179.2, 153.1, 144.5, 133.8, 132.0, 131.7, 129.8, 129.7, 129.5, 129.4, 128.9, 128.3, 125.4, 122.4, 118.7. HRMS (ESI): calcd for C_22_H_17_N_3_O_2_S (2M+Na)^+^ 797.1975, found 797.1986.

(*E*)-5-((4-Methoxyphenyl)imino)-1,3-diphenyl-2-thioxoimidazolidin-4-one (**2i**) was obtained from **1c** (0.71 g, 2.50 mmol), 1-azido-4-methoxybenzene (0.41 g, 2.75 mmol), and PPh_3_ (0.72 g, 2.75 mmol) as a red solid (0.86 g, 88%). ^1^H NMR (400 MHz, CDCl_3_) δ 7.58–7.53 (m, 2H, Ar), 7.53–7.46 (m, 6H, Ar), 7.40 (d, *J* = 7.4 Hz, 2H, Ar), 7.17 (d, *J* = 6.5 Hz, 2H, Ar), 6.83 (d, *J* = 6.6 Hz, 2H, Ar), 3.78 (s, 3H, OCH_3_). ^13^C NMR (101 MHz, CDCl_3_) δ 178.9, 158.3, 153.1, 139.3, 137.6, 134.2, 132.3, 129.6, 129.3, 129.0, 128.4, 123.9, 113.7, 55.5. HRMS (ESI): calcd for (M+H)^+^ 388.1114, found 388.1122.

(*E*)-5-((4-Nitrophenyl)imino)-1,3-diphenyl-2-thioxoimidazolidin-4-one (**2j**) was obtained from **1c** (0.47 g, 1.66 mmol), 1-azido-4-nitrobenzene (0.30 g, 1.83 mmol) and PPh_3_ (0.48 g, 1.83 mmol) as a light yellow solid (0.48 g, 72%). ^1^H NMR (400 MHz, CDCl_3_) δ 8.19 (s, 2H, Ar), 7.64–7.46 (m, 8H, Ar), 7.41 (d, *J* = 7.4 Hz, 2H, Ar), 7.08 (s, 2H, Ar). ^13^C NMR (101 MHz, CDCl_3_) δ 179.2, 152.3, 144.6, 141.1, 133.3, 131.8, 129.8, 129.5, 129.4, 128.7, 128.1, 124.6, 120.4. HRMS (ESI): calcd for C_21_H_14_N_4_O_3_S (M+H)^+^ 403.0859, found 403.0864.

Ethyl (*E*)-2-(5-((4-bromophenyl)imino)-2,4-dioxo-3-phenylimidazolidin-1-yl)acetate (**2k**) was obtained from **1b** (0.46 g, 1.66 mmol), 1-azido-4-bromobenzene (0.36 g, 1.83 mmol), and PPh_3_ (0.48 g, 1.83 mmol) as orange solid (0.17 g, 24%). ^1^H NMR (400 MHz, CDCl_3_) δ 7.48–7.43 (m, 3H, Ar), 7.43–7.37 (m, 4H, Ar), 6.92–6.87 (m, 2H, Ar), 4.59 (s, 2H, NCH_2_), 4.29 (q, *J* = 7.2 Hz, 2H, COOCH_2_), 1.33 (t, *J* = 7.1 Hz, 3H, CH_3_). ^13^C NMR (101 MHz, CDCl_3_) δ 166.8, 153.5, 152.6, 144.6, 139.3, 131.8, 130.2, 129.3, 128.9, 125.9, 122.3, 118.5, 62.3, 40.7, 14.3. HRMS (ESI): calcd for C_19_H_16_BrN_3_O_4_ (M+Na)^+^ 452.0216, found 452.0222.

Ethyl (*E*)-2-(4-((4-bromophenyl)imino)-2,5-dioxo-3-phenylimidazolidin-1-yl)acetate (**2l**) was obtained from **1b** (0.46 g, 1.66 mmol), 1-azido-4-bromobenzene (0.36 g, 1.83 mmol), and PPh_3_ (0.48 g, 1.83 mmol) as yellow solids (0.52 g, 72%). ^1^H NMR (400 MHz, CDCl_3_) δ 7.54–7.48 (m, 4H, Ar), 7.46–7.39 (m, 3H, Ar), 6.84 (d, *J* = 8.5 Hz, 2H, Ar), 4.38 (s, 2H, NCH_2_), 4.25 (q, *J* = 7.1 Hz, 2H, COOCH_2_), 1.30 (t, *J* = 7.1 Hz, 3H, CH_3_). ^13^C NMR (101 MHz, CDCl_3_) δ 166.4, 153.9, 152.4, 144.9, 140.3, 131.8, 131.3, 129.4, 128.9, 127.2, 122.0, 118.3, 62.5, 39.7, 14.2. HRMS (ESI): calcd for C_19_H_16_BrN_3_O_4_ (M+Na)^+^ 452.0216, found 452.0220.

### 3.5. General Procedure for the Synthesis of the Spirocyclic Products ***4a–g*** and ***5a–n***

Diffusion mixing technique (a): A small vial (15 mL volume, diameter 1.3 cm) equipped with a magnetic bar was charged with a mixture of dipolarophile **1** or **2** (1 eq, 0.150 mmol) and hydrazonoyl chloride **3** (1.1 eq, 0.165 mol) in 4 mL of chloroform and then placed in a bigger vial (50 mL volume, diameter 3.5 cm) containing triethylamine (35.85 mmol, 5 mL). The outer vial was tightly closed with a lid, and the reaction mixture was stirred at room temperature for 24–48 h.

Triethylamine dropwise addition (b): A solution of TEA (1.2 eq) in DCM (3 mL) was slowly added for 30 min to a stirring solution of dipolarophile **1** or **2** (1 eq, 0.150 mmol) and hydrazonoyl chloride **3** (1.1 eq, 0.165 mol) in 4 mL of DCM. After the addition, the reaction mixture was stirred at room temperature for 24–48 h.

In both cases, after the reactions were completed (TCL-control), the solvent was removed in vacuo, and the residue was purified by column chromatography on silica gel with EtOAc/hexane or DCM as eluent.

3-(4-Chloro-3-nitrophenyl)-1,6,9-triphenyl-4-thia-1,2,6,9-tetraazaspiro [4.4]non-2-ene-7,8-dione (**4a**) was obtained from **1c** and **3a** as a pale yellow solid (96%^a^/88%^b^). ^1^H NMR (400 MHz, DMSO-d6) δ 8.12 (dd, *J* = 1.9, 0.7 Hz, 1H, Ar), 7.72–7.66 (m, 2H, Ar), 7.52 (dd, *J* = 8.8, 7.3 Hz, 2H, Ar), 7.42 (m, 8H, Ar), 7.27–7.20 (m, 5H, Ar). ^13^C NMR (101 MHz, DMSO-d6) δ 156.3, 148.1, 140.5, 139.4, 132.8, 132.3, 130.1, 130.1, 129.8, 129.5, 129.4, 127.7, 125.8, 124.3, 121.9, 117.4, 112.1. HRMS (ESI): calcd for C_28_H_18_ClN_5_O_4_S (M+H)^+^ 578.0660, found 578.0665.

1-(4-Nitrophenyl)-3,6,9-triphenyl-4-thia-1,2,6,9-tetraazaspiro [4.4]non-2-ene-7,8-dione (**4b**) was obtained from **1c** and **3b** as beige solid (44%^a^/91%^b^). ^1^H NMR (400 MHz, CDCl_3_) δ 8.33–8.27 (m, 2H, Ar), 7.55–7.51 (m, 2H, Ar), 7.46–7.42 (m, 2H, Ar), 7.42–7.33 (m, 9H, Ar), 7.25–7.20 (m, 4H, Ar). ^13^C NMR (101 MHz, CDCl_3_) δ 155.0, 151.7, 148.8, 145.2, 144.4, 131.4, 130.0, 130.0, 129.2, 126.6, 124.9, 124.7, 120.8, 120.8. HRMS (ESI): calcd for C_28_H_19_N_5_O_4_S (M+H)^+^ 522.1231, found 522.1226.

3-(4-Bromophenyl)-1,6,9-triphenyl-4-thia-1,2,6,9-tetraazaspiro [4.4]non-2-ene-7,8-dione (**4c**) was obtained from **1c** and **3c** as white solids (99%^a,b^). ^1^H NMR (400 MHz, CDCl_3_) δ 7.46–7.42 (m, 2H, Ar), 7.42–7.37 (m, 2H, Ar), 7.36–7.31 (m, 8H, Ar), 7.30–7.27 (m, 2H, Ar), 7.26–7.19 (m, 5H, Ar). ^13^C NMR (101 MHz, CDCl_3_) δ 155.78, 140.64, 140.00, 132.55, 132.00, 129.99, 129.65, 129.51, 128.98, 127.55, 127.52, 124.79, 124.43, 118.21, 112.00. HRMS (ESI): calcd for C_28_H_19_BrN_4_O_2_S (M+H)^+^ 555.0485, found 555.0487.

3-Methyl-1,6,9-triphenyl-4-thia-1,2,6,9-tetraazaspiro [4.4]non-2-ene-7,8-dione (**4d**) was obtained from **1c** and **3d** as white solids (88%^a^/74%^b^). ^1^H NMR (400 MHz, CDCl_3_) δ 7.39–7.32 (m, 8H, Ar), 7.26–7.20 (m, 6H, Ar), 7.14 (t, *J* = 7.3 Hz, 1H, Ar), 2.00 (s, 3H, CH_3_). ^13^C NMR (101 MHz, CDCl_3_) δ 155.8, 140.9, 139.2, 132.8, 129.9, 129.6, 129.4, 127.6, 124.1, 117.7, 112.0, 16.5. HRMS (ESI): calcd for C_23_H_18_N_4_O_2_S (2M+Na)^+^ 851.2193, found 851.2186.

Ethyl 2-(3-(4-bromophenyl)-9-(4-ethoxyphenyl)-7,8-dioxo-1-phenyl-4-thia-1,2,6,9-tetraazaspiro [4.4]non-2-en-6-yl)acetate (**4e**) was obtained from **1d** and **3c** as beige solids (77%^a^/49%^b^). ^1^H NMR (400 MHz, CDCl_3_) δ 7.48 (d, *J* = 8.5 Hz, 2H, Ar), 7.39–7.32 (m, 4H, Ar), 7.24 (d, *J* = 8.4 Hz, 2H, Ar), 7.18 (t, *J* = 7.3 Hz, 1H, Ar), 7.08 (d, *J* = 9.0 Hz, 2H, Ar), 6.79 (d, *J* = 9.0 Hz, 2H, Ar), 4.31 (d, *J* = 17.6 Hz, 1H, NCH_2_), 4.28–4.13 (m, 2H, COOCH_2_CH_3_), 4.01–3.90 (m, 3H, NCH_2_+OCH_2_CH_3_), 1.34 (t, *J* = 7.0 Hz, 3H, OCH_2_CH_3_), 1.24 (t, *J* = 7.1 Hz, 3H, COOCH_2_CH_3_). ^13^C NMR (101 MHz, CDCl_3_) δ 166.1, 159.6, 156.4, 155.4, 140.4, 140.0, 132.1, 129.9, 129.0, 128.9, 127.6, 125.2, 124.5, 124.2, 118.8, 115.2, 111.0, 63.7, 62.4, 41.9, 14.7, 14.1. HRMS (ESI): calcd for C_28_H_25_BrN_4_O_5_S (M+H)^+^ 609.0802, found 609.0803.

Ethyl 2-(9-(4-methoxyphenyl)-1-(4-nitrophenyl)-7,8-dioxo-3-phenyl-4-thia-1,2,6,9-tetraazaspiro [4.4]non-2-en-6-yl)acetate (**4f**) was obtained from **1e** and **3b** as beige solids (93%^a^/94%^b^). ^1^H NMR (400 MHz, CDCl_3_) δ 8.25 (d, *J* = 9.3 Hz, 2H, Ar), 7.58–7.52 (m, 2H, Ar), 7.47–7.39 (m, 5H, Ar), 7.03 (d, *J* = 9.0 Hz, 2H, Ar), 6.82 (d, *J* = 9.0 Hz, 2H, Ar), 4.39 (d, *J* = 17.5 Hz, 1H, NCH_2_), 4.20 (qq, *J* = 10.8, 7.1 Hz, 2H, COOCH_2_CH_3_), 4.06 (d, *J* = 17.5 Hz, 1H, NCH_2_), 3.74 (s, 3H, OCH_3_), 1.24 (t, *J* = 7.1 Hz, 3H, COOCH_2_CH_3_). ^13^C NMR (101 MHz, CDCl_3_) δ 165.8, 160.5, 145.7, 131.4, 129.4, 129.2, 129.0, 126.7, 125.7, 123.8, 117.0, 115.1, 110.6, 62.6, 55.6, 42.1, 14.2. HRMS (ESI): calcd for C_27_H_23_N_5_O_7_S (M+Na)^+^ 584.1210, found 584.1217.

6-Allyl-1-(4-nitrophenyl)-3-phenyl-4-thia-1,2,6,9-tetraazaspiro [4.4]non-2-ene-7,8-dione (**4g**) was obtained from **1f** and **3b** as a light yellow solid (61%^a^). ^1^H NMR (400 MHz, CDCl_3_) δ 8.53–8.39 (m, 4H, Ar), 7.96–7.91 (m, 2H, Ar), 7.62–7.53 (m, 3H, Ar), 7.51–7.44 (m, 1H, NH), 5.90 (ddt, *J* = 17.2, 10.2, 5.6 Hz, 1H, CH), 5.29–5.18 (m, 2H, =CH_2_), 4.06–4.00 (m, 2H, NCH_2_). ^13^C NMR (101 MHz, CDCl_3_) δ 168.9, 167.4, 160.2, 158.2, 146.7, 143.6, 133.4, 133.4, 132.4, 129.6, 128.8, 127.1, 124.9, 124.7, 117.1, 42.2. HRMS (ESI): calcd for C_19_H_15_N_5_O_4_S (2M+Na)^+^ 841.1582, found 841.1582.

4-(4-Bromophenyl)-1,3,6,8-tetraphenyl-1,2,4,6,8-pentaazaspiro [4.4]non-2-ene-7,9-dione (**5a**) was obtained from **2d** and **3e** as beige solids (71%^a^/62%^b^). ^1^H NMR (400 MHz, CDCl_3_) δ 7.53–7.50 (m, 2H, Ar), 7.47–7.42 (m, 3H, Ar), 7.42–7.38 (m, 3H, Ar), 7.38–7.35 (m, 4H, Ar), 7.35–7.26 (m, 7H, Ar), 7.15–7.11 (m, 2H, Ar), 7.07–7.02 (m, 1H, Ar), 6.98–6.93 (m, 2H, Ar). ^13^C NMR (101 MHz, CDCl_3_) δ 165.0, 151.8, 146.6, 141.4, 135.7, 133.9, 132.9, 130.6, 130.0, 129.7, 129.5, 129.5, 129.0, 128.7, 128.6, 127.9, 127.6, 126.3, 126.1, 124.9, 122.7, 121.9, 115.6, 97.5. HRMS (ESI): calcd for C_34_H_24_BrN_5_O_2_ (M+H)^+^ 614.1186, found 614.1193.

1,4-Bis(4-nitrophenyl)-6,8-diphenyl-3-(p-tolyl)-1,2,4,6,8-pentaazaspiro [4.4]non-2-ene-7,9-dione (**5b**) was obtained from **2g** and **3f** as orange solid (40%^a^/13%^b^). ^1^H NMR (400 MHz, CDCl_3_) δ 8.28–8.23 (m, 2H, Ar), 8.20–8.16 (m, 2H, Ar), 7.52–7.44 (m, 3H, Ar), 7.39–7.34 (m, 4H, Ar), 7.33–7.27 (m, 3H, Ar), 7.25–7.18 (m, 6H, Ar), 7.13 (d, *J* = 8.1 Hz, 2H, Ar), 2.35 (s, 3H, CH_3_). ^13^C NMR (101 MHz, CDCl_3_) δ 164.0, 151.3, 147.8, 146.5, 145.5, 142.1, 141.9, 141.8, 132.8, 130.1, 130.0, 129.9, 129.8, 129.6, 128.6, 128.0, 126.6, 126.1, 125.7, 125.2, 125.1, 122.2, 113.6, 95.5, 21.6. HRMS (ESI): calcd for C_35_H_25_N_7_O_6_ (M+Na)^+^ 662.1759, found 662.1765.

4-(4-Bromophenyl)-1-(4-nitrophenyl)-6,8-diphenyl-3-(p-tolyl)-1,2,4,6,8-pentaazaspiro [4.4]non-2-ene-7,9-dione (**5c**) was obtained from **2d** and **3f** as beige solids (90%^a^/85%^b^). ^1^H NMR (400 MHz, DMSO-d6) δ 8.34 (d, *J* = 9.4 Hz, 2H, Ar), 7.69 (d, *J* = 8.7 Hz, 2H, Ar), 7.55–7.50 (m, 3H, Ar), 7.49–7.43 (m, 4H, Ar), 7.39–7.35 (m, 3H, Ar), 7.33 (d, *J* = 8.2 Hz, 2H, Ar), 7.23–7.17 (m, 4H, Ar), 7.15 (d, *J* = 8.6 Hz, 2H, Ar), 2.29 (s, 3H,CH_3_). ^13^C NMR (101 MHz, DMSO-d6) δ 163.5, 148.2, 145.2, 140.7, 135.0, 133.1, 132.5, 129.9, 129.9, 129.8, 129.6, 129.5, 128.9, 128.8, 128.6, 127.9, 126.8, 126.5, 126.1, 122.1, 121.5, 113.1, 95.3, 21.0. HRMS (ESI): calcd for C_35_H_25_BrN_6_O_4_ (M+Na)^+^ 695.1013, found 695.1015.

4-(2-Bromophenyl)-1-(4-nitrophenyl)-6,8-diphenyl-3-(p-tolyl)-1,2,4,6,8-pentaazaspiro [4.4]non-2-ene-7,9-dione (**5d**) was obtained from **2c** and **3f** as a yellow solid (84%^a^/83%^b^). Mixture of isomers I and II (1:2.3 in CDCl_3_). Isomer I: ^1^H NMR (400 MHz, CDCl_3_) δ 8.20–8.15 (m, 2H, Ar), 7.68–7.65 (m, 1H, Ar), 7.53–7.49 (m, 2H, Ar), 7.47–7.41 (m, 5H, Ar), 7.29–7.24 (m, 2H, Ar), 7.23–7.17 (m, 4H, Ar), 7.17–7.11 (m, 6H, Ar), 2.34 (s, 3H, CH_3_). ^13^C NMR (101 MHz, CDCl_3_) δ 163.6, 151.6, 150.0, 145.8, 141.4, 141.3, 137.1, 134.2, 133.5, 133.2, 130.6, 130.5, 129.7, 129.6, 129.4, 129.2, 128.7, 127.8, 127.7, 126.0, 125.9, 125.1, 123.3, 122.9, 113.2, 96.2, 21.6. HRMS (ESI): calcd for C_35_H_25_BrN_6_O_4_ (M+Na)^+^ 695.1013, found 695.1016. Isomer II: ^1^H NMR (400 MHz, CDCl_3_) δ 8.24–8.19 (m, 2H, Ar), 7.60–7.55 (m, 2H, Ar), 7.45–7.39 (m, 6H, Ar), 7.34–7.27 (m, 5H, Ar), 7.26–7.21 (m, 3H, Ar), 7.10–7.04 (m, 4H, Ar), 2.33 (s, 3H, CH_3_). ^13^C NMR (101 MHz, CDCl_3_) δ 163.5, 151.5, 149.0, 145.6, 141.3, 141.1, 135.6, 134.9, 133.4, 130.8, 130.4, 129.8, 129.6, 129.5, 129.3, 128.9, 128.8, 128.0, 127.3, 126.1, 126.0, 125.1, 124.6, 124.1, 113.3, 95.5, 21.7. HRMS (ESI): calcd for C35H_25_BrN_6_O_4_ (M+Na)^+^ 695.1013, found 695.1022.

4-(2-Chlorophenyl)-1-(4-nitrophenyl)-6,8-diphenyl-3-(p-tolyl)-1,2,4,6,8-pentaazaspiro [4.4]non-2-ene-7,9-dione (**5e**) was obtained from **2a** and **3f** as an orange solid (87%^a^/81%^b^). Mixture of isomers I and II (1:1.75 in DMSO-d6). ^1^H NMR (400 MHz, DMSO-d6) δ 8.33 (d, *J* = 9.3 Hz, 2H^II^, Ar), 8.28 (d, *J* = 9.2 Hz, 2H^I^, Ar), 7.88 (d, *J* = 7.8 Hz, 1H^II^, Ar), 7.71–7.64 (m, 1H^I^+1H^II^, Ar), 7.56–7.45 (m, 5H^I^+7H^II^, Ar), 7.44–7.35 (m, 3H^I^+3H^II^, Ar), 7.35–7.30 (m, 5H^I^+4H^II^, Ar), 7.29–7.26 (m, 1H^I^, Ar), 7.23 (d, *J* = 8.0 Hz, 2H^I^, Ar), 7.19–7.12 (m, 3H^I^+2H^II^, Ar), 7.06–7.02 (m, 2H^II^, Ar), 2.29 (s, 3H^I^+3H^II^, CH_3_). ^13^C NMR (101 MHz, DMSO-d6) δ 163.1, 163.0, 150.9, 150.7, 149.4, 148.7, 145.2, 145.0, 141.2, 141.0, 140.6, 140.5, 134.4, 133.1, 132.9, 132.6, 131.9, 131.7, 131.7, 131.3, 131.1, 130.9, 130.0, 129.8, 129.5, 129.5, 129.4, 129.3, 129.3, 128.8, 128.7, 128.6, 127.9, 127.3, 126.9, 126.8, 126.7, 126.5, 126.3, 126.0, 125.1, 123.4, 122.7, 113.1, 113.1, 113.0, 95.8, 95.1, 40.2, 40.0, 39.7, 39.5, 39.3, 39.1, 38.9, 21.0, 20.8. HRMS (ESI): calcd for C_35_H_25_ClN_6_O_4_ (M+Na)^+^ 651.1518, found 651.1527.

1-(4-Nitrophenyl)-6,8-diphenyl-3,4-di-p-tolyl-1,2,4,6,8-pentaazaspiro [4.4]non-2-ene-7,9-dione (**5f**) was obtained from **2e** and **3f** as beige solid (71%^a^/88%^b^). ^1^H NMR (400 MHz, DMSO-d6) δ 8.32 (d, *J* = 9.0 Hz, 2H, Ar), 7.52 (d, *J* = 8.0 Hz, 3H, Ar), 7.46 (dd, *J* = 14.1, 6.4 Hz, 4H, Ar), 7.39–7.31 (m, 5H, Ar), 7.28 (d, *J* = 7.9 Hz, 2H, Ar), 7.18 (d, *J* = 7.9 Hz, 2H, Ar), 7.07 (d, *J* = 7.6 Hz, 4H, Ar), 2.32 (s, 3H, CH_3_), 2.28 (s, 3H, CH_3_). ^13^C NMR (101 MHz, DMSO-d6) δ 163.7, 150.8, 148.7, 145.4, 140.8, 140.5, 138.6, 132.8, 132.8, 130.5, 130.0, 129.7, 129.4, 129.4, 128.3, 127.8, 127.1, 126.8, 126.4, 125.6, 122.4, 112.9, 95.6, 20.9, 20.6. HRMS (ESI): calcd for C_36_H_28_N_6_O_4_ (M+Na)^+^ 631.2064, found 631.2072.

4-(4-Methoxyphenyl)-1-(4-nitrophenyl)-6,8-diphenyl-3-(p-tolyl)-1,2,4,6,8-pentaazaspiro [4.4]non-2-ene-7,9-dione (**5g**) was obtained from **2f** and **3f** as orange solids (52%^a^/38%^b^). ^1^H NMR (400 MHz, DMSO-d6) δ 8.31 (d, *J* = 9.5 Hz, 2H, Ar), 7.55–7.50 (m, 3H, Ar), 7.50–7.43 (m, 4H, Ar), 7.39–7.31 (m, 5H, Ar), 7.19 (d, *J* = 7.5 Hz, 2H, Ar), 7.13 (d, *J* = 8.4 Hz, 2H, Ar), 7.10–7.06 (m, 2H, Ar), 7.04 (d, *J* = 9.3 Hz, 2H, Ar), 3.76 (s, 3H, OCH_3_), 2.28 (s, 3H, CH_3_). ^13^C NMR (101 MHz, DMSO-d6) δ 163.8, 159.3, 150.9, 148.9, 145.4, 140.9, 140.4, 133.0, 130.0, 129.7, 129.5, 129.4, 129.4, 129.1, 128.2, 127.8, 127.6, 126.8, 126.4, 125.3, 122.3, 115.2, 112.9, 95.8, 55.6, 21.0. HRMS (ESI): calcd for C_36_H_28_N_6_O_5_ (M+H)^+^ 625.2194, found 625.2199.

4-(4-Bromophenyl)-1-(4-nitrophenyl)-3,6,8-triphenyl-1,2,4,6,8-pentaazaspiro [4.4]non-2-ene-7,9-dione (**5h**) was obtained from **2d** and **3b** as beige solids (63%^a^/50%^b^). ^1^H NMR (400 MHz, CDCl_3_) δ 8.28–8.22 (m, 2H, Ar), 7.50–7.46 (m, 3H, Ar), 7.46–7.41 (m, 4H, Ar), 7.40–7.33 (m, 5H, Ar), 7.30 (m, 5H, Ar), 7.16–7.11 (m, 2H, Ar), 6.99–6.93 (m, 2H, Ar). ^13^C NMR (101 MHz, CDCl_3_) δ 164.3, 151.5, 148.7, 145.7, 141.6, 134.8, 133.3, 133.2, 130.9, 130.2, 129.9, 129.7, 129.5, 129.0, 128.9, 128.2, 128.1, 126.1, 126.0, 125.4, 124.7, 122.8, 113.3, 96.0. HRMS (ESI): calcd for C_34_H_23_BrN_6_O_4_ (M+Na)^+^ 681.0856, found 681.0853.

4-(4-Methoxyphenyl)-1-(4-nitrophenyl)-3,6,8-triphenyl-1,2,4,6,8-pentaazaspiro [4.4]non-2-ene-7,9-dione (**5i**) was obtained from **2b** and **3b** as beige solids (60%^a^/50%^b^). ^1^H NMR (400 MHz, DMSO-d6) δ 8.34–8.30 (m, 3H, Ar), 7.54–7.51 (m, 3H, Ar), 7.50–7.48 (m, 2H, Ar), 7.47–7.43 (m, 5H, Ar), 7.40–7.36 (m, 4H, Ar), 7.14 (d, *J* = 8.6 Hz, 2H, Ar), 7.10–7.07 (m, 2H, Ar), 7.04 (d, *J* = 9.1 Hz, 2H, Ar), 3.76 (s, 3H, OCH_3_). ^13^C NMR (101 MHz, DMSO-d6) δ 163.9, 159.4, 150.9, 148.9, 145.4, 140.6, 133.0, 130.9, 130.0, 129.8, 129.5, 129.5, 129.1, 128.9, 128.2, 127.9, 127.5, 126.9, 126.4, 125.4, 125.3, 115.3, 113.0, 95.9, 55.6. HRMS (ESI): calcd for C_35_H_26_N_6_O_5_ (M+H)^+^ 611.2037, found 611.2041.

3-(4-Chlorophenyl)-1,6,8-triphenyl-4-(p-tolyl)-1,2,4,6,8-pentaazaspiro [4.4]non-2-ene-7,9-dione (**5j**) was obtained from **2e** and **3g** as beige solids (57%^a^/93%^b^). ^1^H NMR (400 MHz, CDCl_3_) δ 7.53 (d, *J* = 7.7 Hz, 2H, Ar), 7.44–7.40 (m, 2H, Ar), 7.40–7.34 (m, 4H, Ar), 7.34–7.27 (m, 6H, Ar), 7.24–7.21 (m, 2H, Ar), 7.13–7.09 (m, 4H, Ar), 7.05–7.01 (m, 1H, Ar), 6.97 (d, *J* = 8.3 Hz, 2H, Ar), 2.34 (s, 3H, CH_3_). ^13^C NMR (101 MHz, CDCl_3_) δ 165.1, 151.9, 146.2, 141.4, 138.6, 135.8, 134.1, 133.5, 130.7, 130.4, 129.7, 129.4, 129.4, 129.1, 128.9, 128.8, 127.4, 127.3, 126.3, 125.2, 124.9, 122.4, 115.3, 97.8, 21.2. HRMS (ESI): calcd for C_35_H_26_ClN_5_O_2_ (M+H)^+^ 584.1848, found 584.1849.

3-Methyl-4-(4-nitrophenyl)-1,6,8-triphenyl-1,2,4,6,8-pentaazaspiro [4.4]non-2-ene-7,9-dione (**5k**) was obtained from **2g** and **3d** as orange solid (88%^a^/59%^b^). ^1^H NMR (400 MHz, CDCl_3_): δ 8.27–8.22 (m, 2H, Ar), 7.46–7.37 (m, 7H, Ar), 7.36–7.31 (m, 3H, Ar), 7.23–7.19 (m, 4H, Ar), 7.15–7.11 (m, 2H, Ar), 7.05 (m, 1H, Ar), 2.03 (s, 3H, CH_3_). ^13^C NMR (101 MHz, CDCl_3_) δ 165.5, 151.5, 147.1, 144.1, 141.7, 140.6, 133.9, 130.4, 129.8, 129.6, 129.5, 129.2, 128.1, 127.7, 126.0, 125.3, 124.8, 122.9, 115.9, 96.9, 12.1. HRMS (ESI): calcd for C_29_H_22_N_6_O_4_ (M+H)^+^ 519.1775, found 519.1772.

4-(4-Bromophenyl)-3-methyl-1,6,8-triphenyl-1,2,4,6,8-pentaazaspiro [4.4]non-2-ene-7,9-dione (**5l**) was obtained from **2d** and **3d** as orange solid (73%^a^/57%^b^). ^1^H NMR (400 MHz, CDCl_3_) δ 7.54–7.50 (m, 2H, Ar), 7.49–7.45 (m, 2H, Ar), 7.45–7.36 (m, 5H, Ar), 7.35–7.28 (m, 3H, Ar), 7.23–7.19 (m, 2H, Ar), 7.15–7.10 (m, 2H, Ar), 7.03–6.98 (m, 1H, Ar), 6.96–6.90 (m, 2H, Ar), 1.93 (s, 3H, CH_3_). ^13^C NMR (101 MHz, CDCl_3_) δ 165.9, 151.6, 145.4, 142.0, 134.3, 133.3, 133.2, 130.6, 130.6, 129.7, 129.4, 129.0, 127.2, 126.2, 124.6, 123.4, 122.3, 115.3, 97.1, 11.6. HRMS (ESI): calcd for C_29_H_22_BrN_5_O_2_ (M+H)^+^ 552.1030, found 552.1023.

Ethyl 2-(4-(4-bromophenyl)-1-(4-nitrophenyl)-7,9-dioxo-3,8-diphenyl-1,2,4,6,8-pentaazaspiro [4.4]non-2-en-6-yl)acetate (**5m**) was obtained from **2k** and **3b** as orange solid (15%^b^). ^1^H NMR (400 MHz, CDCl_3_) δ 8.23 (d, *J* = 9.2 Hz, 2H, Ar), 7.51–7.47 (m, 3H, Ar), 7.46–7.43 (m, 2H, Ar), 7.42–7.40 (m, 2H, Ar), 7.37–7.33 (m, 3H, Ar), 7.29 (d, *J* = 9.2 Hz, 2H, Ar), 7.22 (d, *J* = 8.1 Hz, 2H, Ar), 7.09–7.02 (m, 2H, Ar), 4.32 (d, *J* = 17.6 Hz, 1H, NCH_2_), 4.21–4.07 (m, 3H, NCH_2_+COOCH_2_CH_3_), 1.19 (t, *J* = 7.1 Hz, 3H, COOCH_2_CH_3_). ^13^C NMR (101 MHz, CDCl_3_) δ 167.4, 164.0, 152.4, 148.8, 146.0, 141.8, 135.6, 133.1, 131.0, 130.1, 129.6, 129.3, 128.9, 128.7, 128.2, 125.9, 125.7, 125.5, 122.5, 113.7, 95.4, 62.3, 41.0, 14.1. HRMS (ESI): calcd for C_32_H_25_BrN_6_O_6_ (M+Na)^+^ 691.0911, found 691,0913.

Ethyl 2-(4-(4-bromophenyl)-1-(4-nitrophenyl)-7,9-dioxo-3,6-diphenyl-1,2,4,6,8-pentaazaspiro [4.4]non-2-en-8-yl)acetate (**5n**) was obtained from **2l** and **3b** as orange solid (76%^b^). ^1^H NMR (400 MHz, DMSO-d6) δ 8.24–8.19 (m, 2H, Ar), 7.60–7.56 (m, 2H, Ar), 7.46–7.41 (m, 2H, Ar), 7.41–7.37 (m, 5H, Ar), 7.37–7.31 (m, 3H, Ar), 7.30–7.26 (m, 2H, Ar), 7.03–6.97 (m, 2H, Ar), 4.56–4.45 (m, 2H, NCH_2_), 4.20 (qd, *J* = 7.0, 1.4 Hz, 2H, COOCH_2_CH_3_), 1.21 (t, *J* = 7.1 Hz, 3H, COOCH_2_CH_3_). ^13^C NMR (101 MHz, DMSO-d6) δ 167.0, 164.1, 151.1, 148.3, 144.9, 140.7, 134.3, 132.9, 132.3, 130.9, 129.8, 129.0, 128.8, 128.5, 127.9, 126.0, 125.7, 125.0, 121.4, 113.5, 95.3, 61.9, 14.0. HRMS (ESI): calcd for C_32_H_25_BrN_6_O_6_ (M+Na)^+^ 691.0911, found 691.0913.

### 3.6. Reagents for MTT Test

Sigma-Aldrich (Schnelldorf, Germany) provided 3-(4,5-dimethylthiazol-2-yl)-2,5-diphenyltetrazolium bromide (MTT). Trypan blue, phosphate-buffered saline (PBS), and dimethyl sulfoxide (DMSO) were purchased from PanEco (Moscow, Russia). Fetal bovine serum was obtained from HyClone (Logan, UT, USA), along with flasks and plates purchased from Nunc (Moscow, Russia).

### 3.7. Cell Lines and Cytotoxicity Evaluation

The human colon adenocarcinoma cell line (HCT116) was purchased from ATCC (Manassas, VA, USA). Cancer cells were routinely grown in RPMI 1640 culture medium, supplemented with 10% fetal bovine serum, glutamine, and 100 U/mL penicillin. HCT116 cell line was grown in flasks in RPMI 1640 fresh culture medium with supplements at 37 °C and 5% CO_2_. Cells were grown as monolayer cultures, and the cells in the exponential growth phase were trypsinized and suspended in RPMI 1640 medium supplemented.

### 3.8. Cytotoxicity

To evaluate the cytotoxicity of compounds *in vitro*, we placed cells in (4–7) × 10^3^ cells/mL concentrations in 96-well culture plates for 24 h. Cells were counted after treatment with Trypan blue solution (0.4%). They were then exposed to different concentrations of compounds in two-fold serial (50–100 µM) dilutions in pre-incubated cells at 37 °C for 72 h. In control wells with untreated cells, only (DMCO + PBS) were added. Cell viability was measured by the standard MTT test [31]. The absorbance was measured at 540 nm using a Multiskan™ FC microplate photometer and Skanlt software 6.1 RE for microplate readers, both from Thermo Scientific (Waltham, MA, USA).

### 3.9. Statistical Analysis

In vitro experiments were carried out in triplicate. Graphpad Prism version 9.0 was used to determine the IC50. The data for IC50 are presented as the mean ± standard deviation (SD).

## 4. Conclusions

A series of new spiro derivatives containing pyrazoline and imidazoledione fragments were synthesized by 1,3-dipolar cycloaddition of nitrile imines, which were generated in situ from hydrazonoyl chlorides, to the parabanic acid derivatives. It was shown that the cycloaddition reactions proceed regioselectively in all cases, regardless of the presence of electron-donating or electron-withdrawing substituents in the 1,3-dipole molecules. The study of the 1,3-dipolar cycloaddition reaction of nitrile imines by DFT calculation methods showed that 1,3-dipoles act as nucleophiles in the reaction with 3-phenyl-5-methylidenehydantoin, and the 1,3-dipolar cycloaddition reaction with normal electronic demands (NED) should have been realized.

The reactions of 2-thioxoimidazolidine-4,5-diones and 5-arylimino-1,3-diphenylimidazolidine-2,4,5-triones with nitrile imines were carried out using two alternative synthetic approaches, which differed in the method of adding a base generating a dipole from hydrazonoyl chloride (“classic” dropwise addition method and a diffusion mixing technique). It was found that the diffusion mixing technique typically gives the same or better yield of the target product compared to the addition of amine drop by drop.

The cytotoxicity of some of the obtained spiro derivatives was evaluated using the MTT test on the human colorectal carcinoma cell line HCT116. The tested compounds demonstrated moderate cytotoxic activity with IC_50_ values in the range of ~25–45 µM.

## Figures and Tables

**Table 1:** Synthesis of imidazolidine-4,5-diones **1a–f**.

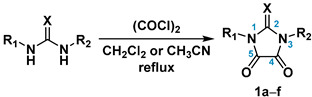				
Compound №	X	R_1_	R_2_	Yield ^a^, %
**1a** ^b^	O	Ph	Ph	99
**1b** ^b^	O	Ph	CH_2_COOEt	99
**1c** ^c^	S	Ph	Ph	98
**1d** ^c^	S	4-EtO-C_6_H_4_	CH_2_COOEt	93
**1e** ^c^	S	4-MeO-C_6_H_4_	CH_2_COOEt	77
**1f** ^c^	S	All	H	47

^a^ Isolated yield; ^b^ DCM, reflux 2 h; ^c^ CH_3_CN, reflux 6 h, purified by flash chromatography.

**Table 2:** Synthesis of imines **2a–j**.

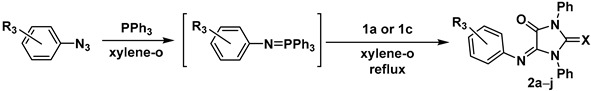			
Compound №	X	R_3_	Yield ^a^, %
**2a**	O	4-Cl	79
**2b**	O	2-Cl	60
**2c**	O	4-Br	60
**2d**	O	2-Br	76
**2e**	O	4-Me	84
**2f**	O	4-OMe	65
**2g**	O	4-NO_2_	75
**2h**	S	4-Br	82
**2i**	S	4-OMe	88
**2j**	S	4-NO_2_	72

^a^ Isolated yield.

**Table 3:** Reaction of 2-thioxoimidazolidine-4,5-diones **1c–f** and hydrazonoyl chlorides **3a–d**.

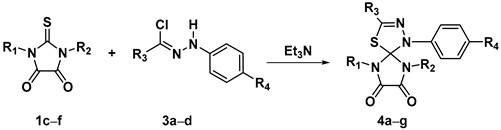						
Compound №	R_1_	R_2_	R_3_	R_4_	Diffusion Mixing	Dropwise Addition
					Yield ^a^, %	Yield ^a^, %
**4a**	Ph	Ph	3-NO_2_-4-Cl-C_6_H_3_	H	96	88
**4b**	Ph	Ph	Ph	4-NO_2_	44	91
**4c**	Ph	Ph	4-Br-C_6_H_4_	H	99	99
**4d**	Ph	Ph	Me	H	88	74
**4e**	4-EtO-C_6_H_4_	CH_2_COOEt	4-Br-C_6_H_4_	H	77	49
**4f**	4-MeO-C_6_H_4_	CH_2_COOEt	Ph	4-NO_2_	93	94
**4g**	All	H	Ph	4-NO_2_	61	- ^b^

^a^ Isolated yield; ^b^ Yield was not defined.

**Table 4:** The influence of reaction conditions on the formation of a by-product.

Entry	Solvent	T	Yield ^a^ of 4b, %	Yield ^a^ of 7, %
1	CH_3_OH	rt	60	-
2	CH_3_CN	rt	75	<1 ^b^
3	CHCl_3_	−17 °C	49	32
4	CHCl_3_	rt	44	35
5	(CH_3_)_2_CO	rt	34	45

^a^ Isolated yield, diffusion mixing technique; ^b^ Traces.

**Table 5:** Reaction of 5-arylimino-1,3-diphenylimidazolidine-2,4-diones **2b–g** and hydrazonoyl chlorides **3b–g**.

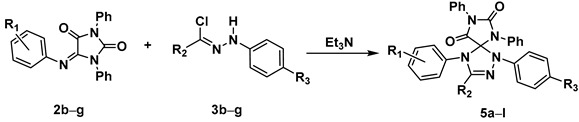					
Compound №	R_1_	R_2_	R_3_	Diffusion Mixing	Dropwise Addition
				Yield ^a^, %	Yield ^a^, %
**5a**	4-Br	Ph	H	71	62
**5b**	4-NO_2_	4-Me-C_6_H_4_	NO_2_	40	13
**5** **c**	4-Br	4-Me-C_6_H_4_	NO_2_	90	85
**5d**	2-Br	4-Me-C_6_H_4_	NO_2_	84	83
**5e**	2-Cl	4-Me-C_6_H_4_	NO_2_	87	81
**5f**	4-Me	4-Me-C_6_H_4_	NO_2_	71	88
**5g**	4-OMe	4-Me-C_6_H_4_	NO_2_	52	38
**5h**	4-Br	Ph	NO_2_	62	50
**5i**	4-OMe	Ph	NO_2_	60	50
**5j**	4-Me	4-Cl-C_6_H_4_	H	57	93
**5k**	4-NO_2_	Me	H	88	59
**5l**	4-Br	Me	H	73	57

^a^ Isolated yield.

**Table 6:** Cytotoxicity monitoring results (MTT test, HCT116 cell line) and literature data [12,30].

Compound №	IC_50_, µM	R_1_	R_2_	R_3_	R_4_	Structure Type
**4a**	24.75 ± 0.01	Ph	Ph	3-NO_2_-4-Cl-C_6_H_3_	H	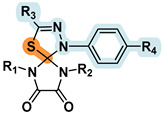
**4b**	35.04 ± 0.06	Ph	Ph	Ph	4-NO_2_	
**4e**	48.51 ± 0.03	4-EtO-C_6_H_4_	CH_2_COOEt	4-Br-C_6_H_4_	H	
**4f**	29.61 ± 0.08	4-MeO-C_6_H_4_	CH_2_COOEt	Ph	4-NO_2_	
**5c**	40.99 ± 0.01	4-Br	4-Me-C_6_H_4_	NO_2_	-	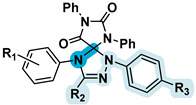
**5f**	43.41 ± 0.01	4-Me	4-Me-C_6_H_4_	NO_2_	-	
**5j**	35.07 ± 0.01	4-Me	4-Cl-C_6_H_4_	H	-	
**Reference**	**IC_50_, µM**		**Cell line**	**R_1_**	**R_2_**	**Structure type**
[12]	1.2–3.4		LNCaP	Ar/CH_3_CHPh	Cl/Br	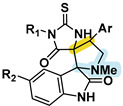
[30]	4–30		HEK293T	H/Me	H/Me	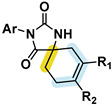

## Data Availability

The data are available from the authors upon request.

## Supplementary Materials

The following supporting information can be downloaded at: https://www.mdpi.com/article/10.3390/ijms25010018/s1.
