# Supplementary material for: [3+2]-Cycloaddition of Nitrile Imines to Parabanic Acid Derivatives—An Approach to Novel Spiroimidazolidinediones
Source: Int J Mol Sci. 2023 Dec 19;25(1):18. doi: 10.3390/ijms25010018 (PMC10778941; doi:10.3390/ijms25010018)
Supplement: Supplementary file 1 [file ijms-25-00018-s001.zip › ijms-2769980-supplementary.pdf]

# [3+2]-Cycloaddition of Nitrile Imines to Parabanic Acid Derivatives – an Approach to Novel Spiroimidazolidinediones

**Juliana V. Kuznetsova[1], Varvara T. Tkachenko[1], Lada M. Petrovskaya[1], Maria E. Filkina[1], Dmitry E. Shybanov[1], Yuri K. Grishin[1], Vitaly A. Roznyatovsky[1], Viktor A. Tafeenko[1], Anna S. Pestretsova[2,3], Vera A. Yakovleva[4], Vadim S. Pokrovsky[2,4,5], Maxim E. Kukushkin[1] and Elena K. Beloglazkina[1]***

[1]Department of Chemistry, M.V. Lomonosov Moscow State University, Leninskie Gory, 1-3, 119991 Moscow, Russia
[2] Blokhin National Medical Research Center of Oncology, Ministry of Health of the Russian Federation, 115478 Moscow, Russia
[3] Occupational Health Risks Lab, Peoples' Friendship University of Russia (RUDN University), 117198 Moscow, Russia
[4] Department of biochemistry, People's Friendship University of Russia (RUDN University), 117198 Moscow, Russia
[5] Research Institute of Molecular and Cellular Medicine, People's Friendship University of Russia (RUDN University), 117198 Moscow, Russia

* Correspondence: beloglazki@mail.ru

# Table of contents

$^{1}$H, $^{13}$C NMR and HRMS spectral data of imidazolidine-4,5-diones 1a-f ........ 4

1,3-Diphenylimidazolidine-2,4,5-trione (1a) ........ 4

Ethyl 2-(2,4,5-trioxo-3-phenylimidazolidin-1-yl)acetate (1b) ........ 5

1,3-Diphenyl-2-thioxoimidazolidine-4,5-dione (1c) ........ 6

Ethyl 2-(3-(4-ethoxyphenyl)-4,5-dioxo-2-thioxoimidazolidin-1-yl)acetate (1d) ........ 7

Ethyl 2-(3-(4-methoxyphenyl)-4,5-dioxo-2-thioxoimidazolidin-1-yl)acetate (1e) ........ 8

1-Allyl-2-thioxoimidazolidine-4,5-dione (1f) ........ 9

$^{1}$H, $^{13}$C NMR and HRMS spectral data of 5-aryliminoimidazolidine-4,5-diones 2a-m ........ 10

(*E*)-5-((4-Chlorophenyl)imino)-1,3-diphenylimidazolidine-2,4-dione (2a) ........ 10

(*E*)-5-((2-Chlorophenyl)imino)-1,3-diphenylimidazolidine-2,4-dione (2b) ........ 11

(*E*)-5-((4-Bromophenyl)imino)-1,3-diphenylimidazolidine-2,4-dione (2c) ........ 12

(*E*)-5-((2-Bromophenyl)imino)-1,3-diphenylimidazolidine-2,4-dione (2d) ........ 13

(*E*)-1,3-Diphenyl-5-(p-tolylimino)imidazolidine-2,4-dione (2e) ........ 14

(*E*)-5-((4-Methoxyphenyl)imino)-1,3-diphenylimidazolidine-2,4-dione (2f) ........ 15

(*E*)-5-((4-Nitrophenyl)imino)-1,3-diphenylimidazolidine-2,4-dione (2g) ........ 16

(*E*)-5-((4-bromophenyl)imino)-1,3-diphenyl-2-thioxoimidazolidin-4-one (2h) ........ 17

(*E*)-5-((4-Methoxyphenyl)imino)-1,3-diphenyl-2-thioxoimidazolidin-4-one (2i) ........ 19

(*E*)-5-((4-Nitrophenyl)imino)-1,3-diphenyl-2-thioxoimidazolidin-4-one (2j) ........ 20

Ethyl (*E*)-2-(5-((4-bromophenyl)imino)-2,4-dioxo-3-phenylimidazolidin-1-yl)acetate (2k) ..... 21

Ethyl (*E*)-2-(4-((4-bromophenyl)imino)-2,5-dioxo-3-phenylimidazolidin-1-yl)acetate (2l) ...... 22

$^{1}$H, $^{13}$C NMR and HRMS spectral data of 4-thia-1,2,6,9-tetraazaspiro[4.4]non-2-ene-7,8-diones 4a-g ........ 23

3-(4-Chloro-3-nitrophenyl)-1,6,9-triphenyl-4-thia-1,2,6,9-tetraazaspiro[4.4]non-2-ene-7,8-dione (4a) ........ 23

1-(4-Nitrophenyl)-3,6,9-triphenyl-4-thia-1,2,6,9-tetraazaspiro[4.4]non-2-ene-7,8-dione (4b)... 24

3-(4-Bromophenyl)-1,6,9-triphenyl-4-thia-1,2,6,9-tetraazaspiro[4.4]non-2-ene-7,8-dione (4c). 25

3-Methyl-1,6,9-triphenyl-4-thia-1,2,6,9-tetraazaspiro[4.4]non-2-ene-7,8-dione (4d) ........ 26

Ethyl 2-(3-(4-bromophenyl)-9-(4-ethoxyphenyl)-7,8-dioxo-1-phenyl-4-thia-1,2,6,9-tetraazaspiro[4.4]non-2-en-6-yl)acetate (4e) ........ 28

Ethyl 2-(9-(4-methoxyphenyl)-1-(4-nitrophenyl)-7,8-dioxo-3-phenyl-4-thia-1,2,6,9-tetraazaspiro[4.4]non-2-en-6-yl)acetate (4f) ........ 29

6-Allyl-1-(4-nitrophenyl)-3-phenyl-4-thia-1,2,6,9-tetraazaspiro[4.4]non-2-ene-7,8-dione (4g). 30

$^{1}$H, $^{13}$C NMR and HRMS spectral data of 1,2,4,6,8-pentaazaspiro[4.4]non-2-ene-7,9-diones 5a-n31

4-(4-Bromophenyl)-1,3,6,8-tetraphenyl-1,2,4,6,8-pentaazaspiro[4.4]non-2-ene-7,9-dione (5a) 31

1,4-Bis(4-nitrophenyl)-6,8-diphenyl-3-(p-tolyl)-1,2,4,6,8-pentaazaspiro[4.4]non-2-ene-7,9-dione (5b) ..... 32

4-(4-Bromophenyl)-1-(4-nitrophenyl)-6,8-diphenyl-3-(p-tolyl)-1,2,4,6,8-pentaazaspiro[4.4]non-2-ene-7,9-dione (5c) ..... 33

4-(2-Bromophenyl)-1-(4-nitrophenyl)-6,8-diphenyl-3-(p-tolyl)-1,2,4,6,8-pentaazaspiro[4.4]non-2-ene-7,9-dione (5d) ..... 34

4-(2-Chlorophenyl)-1-(4-nitrophenyl)-6,8-diphenyl-3-(p-tolyl)-1,2,4,6,8-pentaazaspiro[4.4]non-2-ene-7,9-dione (5e) ..... 36

1-(4-Nitrophenyl)-6,8-diphenyl-3,4-di-p-tolyl-1,2,4,6,8-pentaazaspiro[4.4]non-2-ene-7,9-dione (5f) ..... 37

4-(4-Methoxyphenyl)-1-(4-nitrophenyl)-6,8-diphenyl-3-(p-tolyl)-1,2,4,6,8-pentaazaspiro[4.4]non-2-ene-7,9-dione (5g) ..... 38

4-(4-Bromophenyl)-1-(4-nitrophenyl)-3,6,8-triphenyl-1,2,4,6,8-pentaazaspiro[4.4]non-2-ene-7,9-dione (5h) ..... 39

4-(4-Methoxyphenyl)-1-(4-nitrophenyl)-3,6,8-triphenyl-1,2,4,6,8-pentaazaspiro[4.4]non-2-ene-7,9-dione (5i) ..... 40

3-(4-Chlorophenyl)-1,6,8-triphenyl-4-(p-tolyl)-1,2,4,6,8-pentaazaspiro[4.4]non-2-ene-7,9-dione (5j) ..... 41

3-Methyl-4-(4-nitrophenyl)-1,6,8-triphenyl-1,2,4,6,8-pentaazaspiro[4.4]non-2-ene-7,9-dione (5k) ..... 42

4-(4-Bromophenyl)-3-methyl-1,6,8-triphenyl-1,2,4,6,8-pentaazaspiro[4.4]non-2-ene-7,9-dione (5l) ..... 43

Ethyl 2-(4-(4-bromophenyl)-1-(4-nitrophenyl)-7,9-dioxo-3,8-diphenyl-1,2,4,6,8-pentaazaspiro[4.4]non-2-en-6-yl)acetate (5m) ..... 45

Ethyl 2-(4-(4-bromophenyl)-1-(4-nitrophenyl)-7,9-dioxo-3,6-diphenyl-1,2,4,6,8-pentaazaspiro[4.4]non-2-en-8-yl)acetate (5n) ..... 46

$^1$H NMR spectral data of compounds 5 and 6 ..... 47

(Z)-3-Phenyl-2-(phenylimino)thiazolidine-4,5-dione (5) ..... 47

(Z)-3-(4-nitrophenyl)-N,5-diphenyl-1,3,4-thiadiazol-2(3H)-imine (6) ..... 49

IR spectral data of 1c, 2i, 2j and 5 ..... 50

1,3-Diphenyl-2-thioxoimidazolidine-4,5-dione (1c) ..... 50

5-((4-Methoxyphenyl)imino)-1,3-diphenyl-2-thioxoimidazolidin-4-one (2i) ..... 51

5-((4-Nitrophenyl)imino)-1,3-diphenyl-2-thioxoimidazolidin-4-one (2j) ..... 52

3-Phenyl-2-(phenylimino)thiazolidine-4,5-dione (5) ..... 53

Crystallographic data of 5c, 5g and 5i ..... 54

## $^{1}H$, $^{13}C$ NMR and HRMS spectral data of imidazolidine-4,5-diones 1a-f

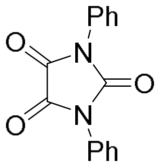


### 1,3-Diphenylimidazolidine-2,4,5-trione (1a)

$^{1}H$ NMR (400 MHz, $CDCl_3$) δ 7.57 – 7.52 (m, 4H, Ar), 7.51 – 7.45 (m, 6H, Ar).

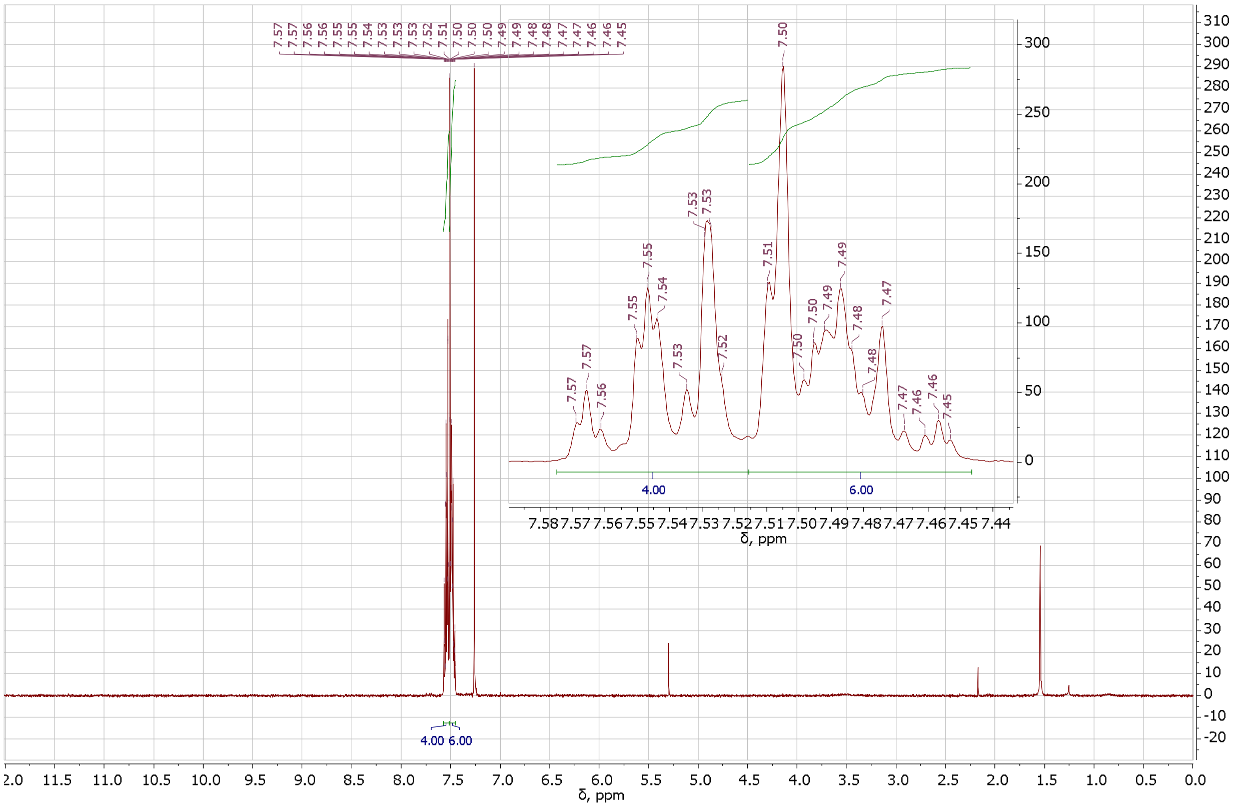

### Ethyl 2-(2,4,5-trioxo-3-phenylimidazolidin-1-yl)acetate (1b)

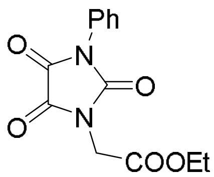


$^{1}$H NMR (400 MHz, $CDCl_3$) δ 7.55 – 7.49 (m, 2H, Ar), 7.48 – 7.41 (m, 3H, Ar), 4.49 (s, 2H, $NCH_2$), 4.28 (q, *J* = 7.1 Hz, 2H, $COOCH_2$), 1.32 (t, *J* = 7.1 Hz, 3H, $CH_3$).

$^{13}$C NMR (101 MHz, $CDCl_3$) δ 166.0, 155.7, 155.3, 152.2, 129.8, 129.6, 129.3, 125.8, 62.7, 40.0, 14.2.

HRMS (ESI): calcd for $C_{13}H_{12}N_2O_5$ $(M+Na)^+$ 299.0638, found 299.0637.

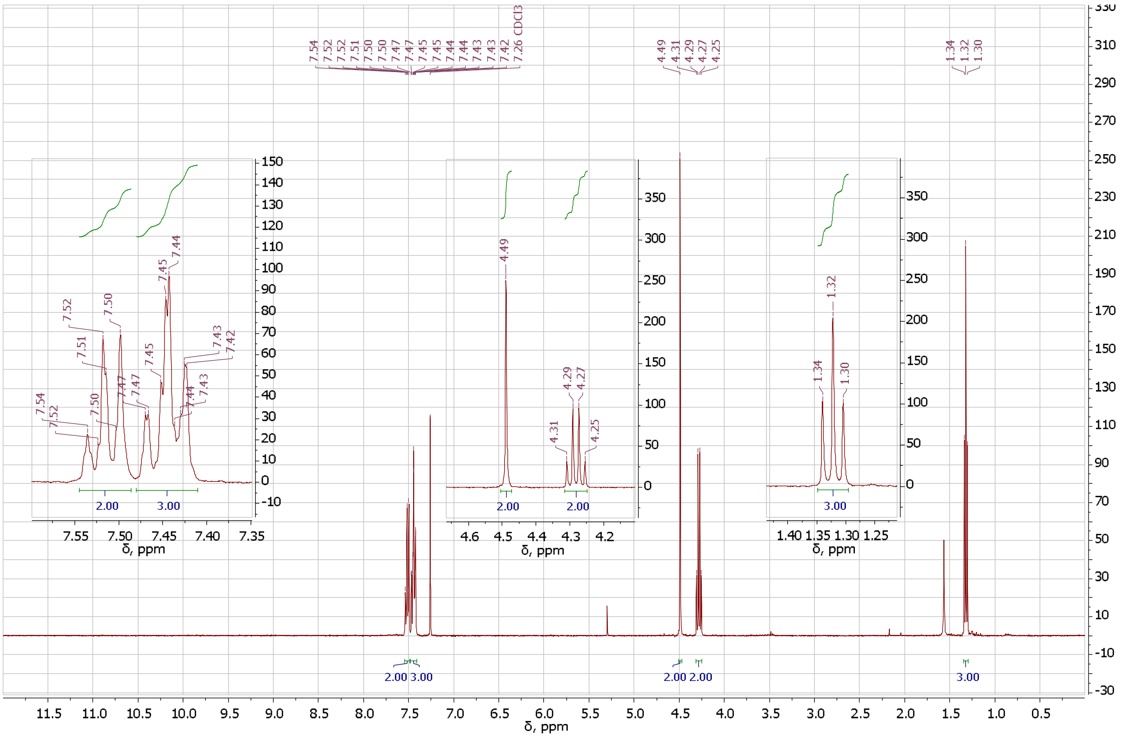


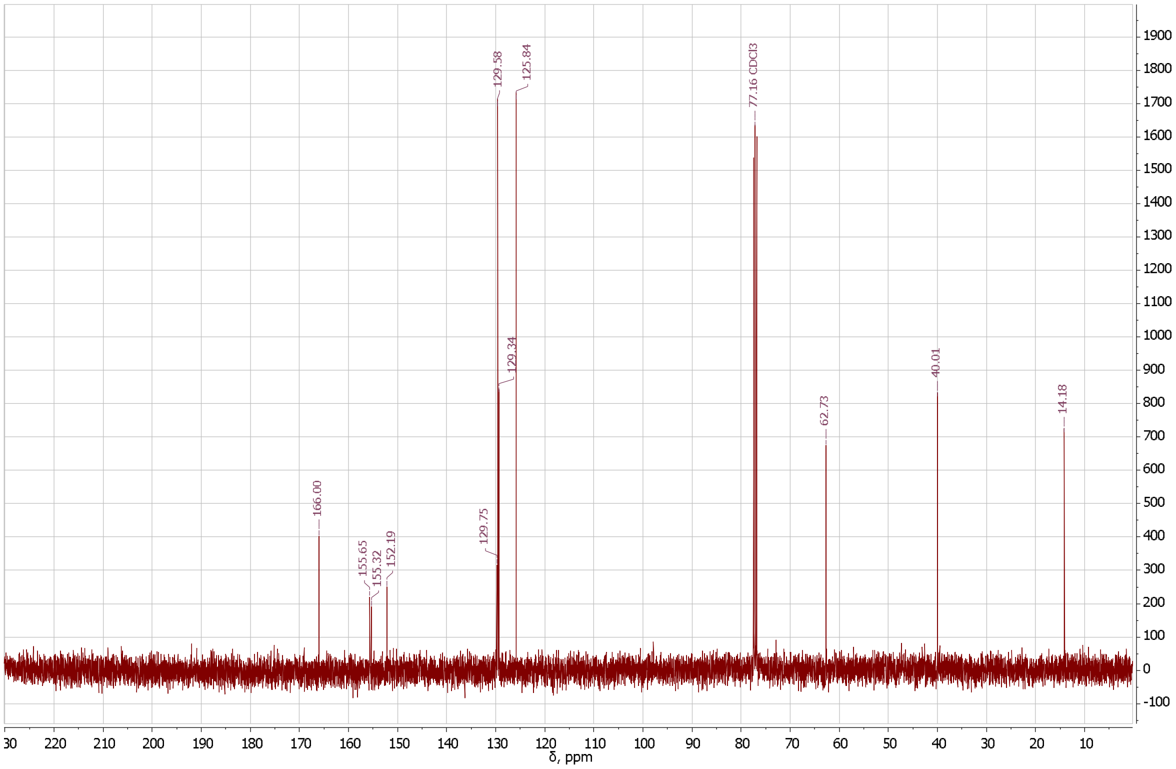

## 1,3-Diphenyl-2-thioxoimidazolidine-4,5-dione (1c)

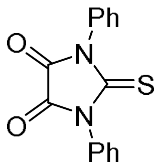

$^1$H NMR (400 MHz, $CDCl_3$) δ 7.59 – 7.50 (m, 6H, Ar), 7.42 – 7.37 (m, 4H, Ar).

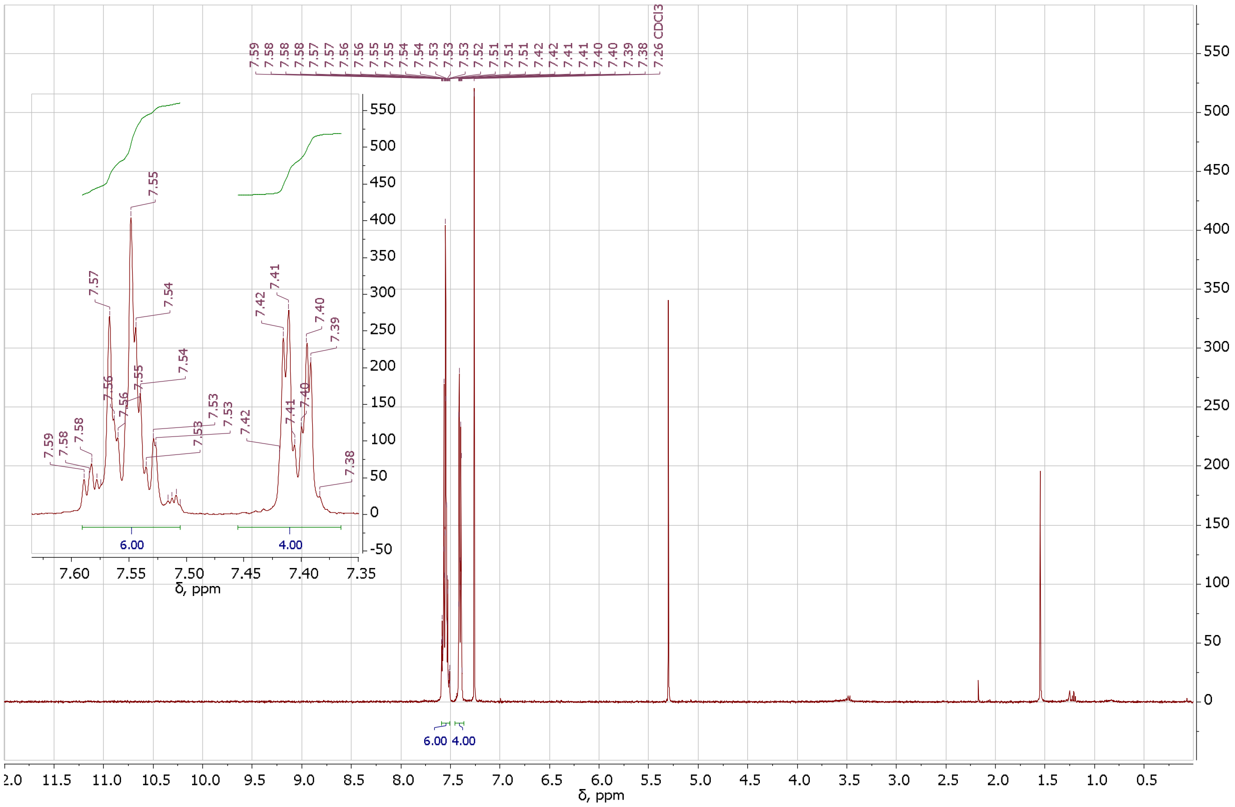

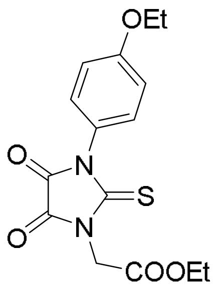


**Ethyl 2-(3-(4-ethoxyphenyl)-4,5-dioxo-2-thioxoimidazolidin-1-yl)acetate (1d)**

$^1$H NMR (400 MHz, $CDCl_3$) δ 7.25 – 7.20 (m, 2H, Ar), 7.02 – 6.97 (m, 2H, Ar), 4.76 (s, 2H, $NCH_2$), 4.27 (q, *J* = 7.1 Hz, 2H, $COOC\underline{H_2}CH_3$), 4.07 (q, *J* = 7.0 Hz, 2H, $OC\underline{H_2}CH_3$), 1.44 (t, *J* = 7.0 Hz, 3H, $OCH_2C\underline{H_3}$), 1.31 (t, *J* = 7.2 Hz, 3H, $COOCH_2C\underline{H_3}$).

$^{13}$C NMR (101 MHz, $CDCl_3$) δ 180.0, 165.9, 160.0, 154.8, 154.4, 129.2, 123.7, 115.3, 63.9, 62.5, 42.7, 14.9, 14.2.

HRMS (ESI): calcd for $C_{15}H_{16}N_2O_5S$ $(M+Na)^+$ 359.0672, found 359.0675.

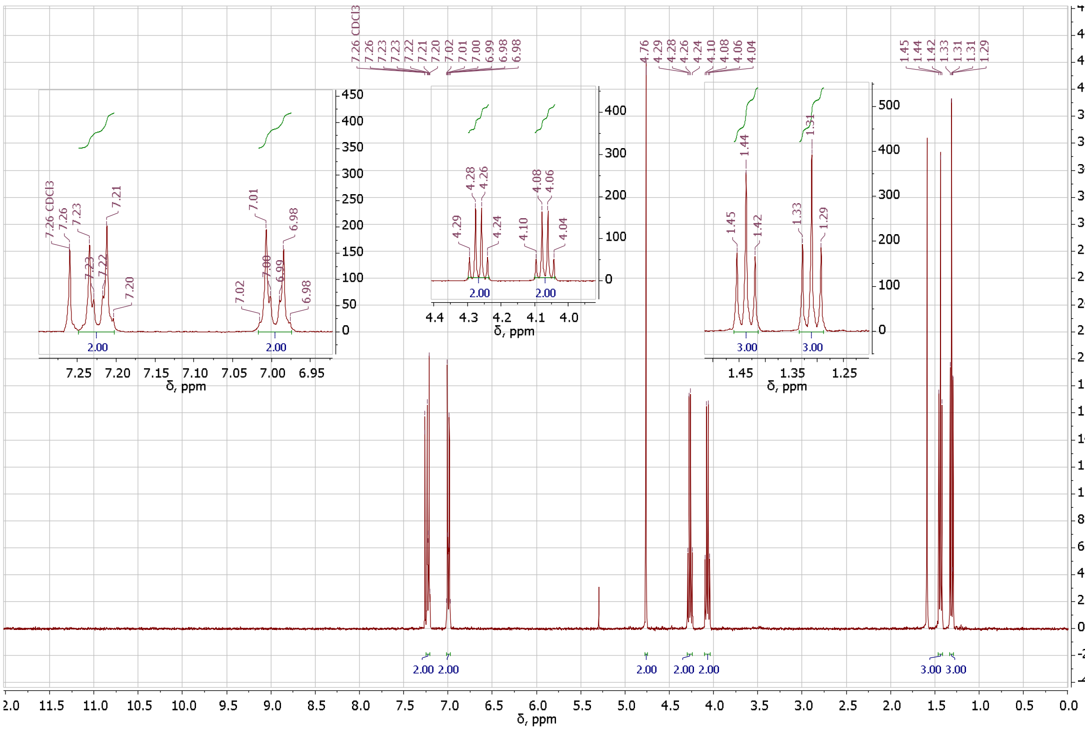

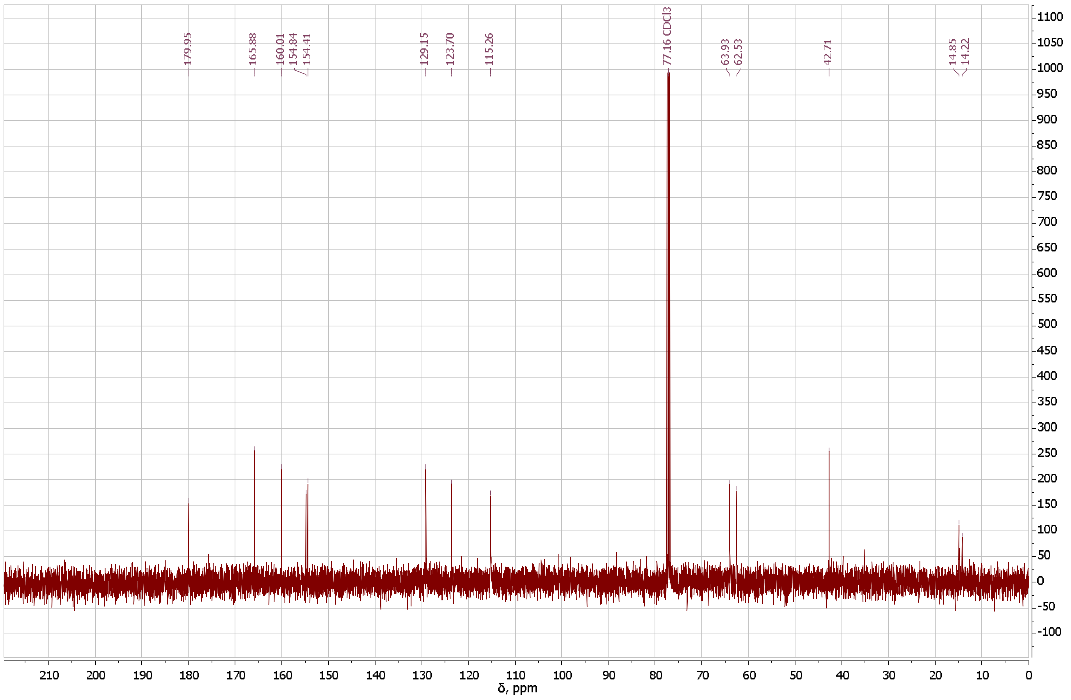


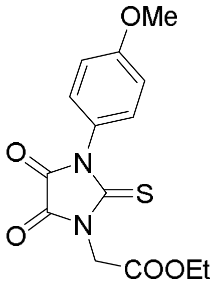


**Ethyl 2-(3-(4-methoxyphenyl)-4,5-dioxo-2-thioxoimidazolidin-1-yl)acetate (1e)**

$^{1}$H NMR (400 MHz, $CDCl_3$) δ 7.26 – 7.22 (m, 2H), 7.04 – 6.99 (m, 2H), 4.77 (s, 2H, $NCH_2$), 4.27 (q, $J$ = 7.1 Hz, 2H, COOC$\underline{H_2}$$CH_3$), 3.85 (s, 3H, $OCH_3$), 1.31 (t, $J$ = 7.1 Hz, 3H, COOC$H_2$C$\underline{H_3}$).

$^{13}$C NMR (101 MHz, $CDCl_3$) δ 179.9, 165.9, 160.6, 154.8, 154.4, 129.2, 123.9, 114.9, 62.6, 55.7, 42.7, 14.2.

HRMS (ESI): calcd for $C_{14}H_{14}N_2O_5S$ $(2M+Na)^+$ 667.1139, found 667.1138.

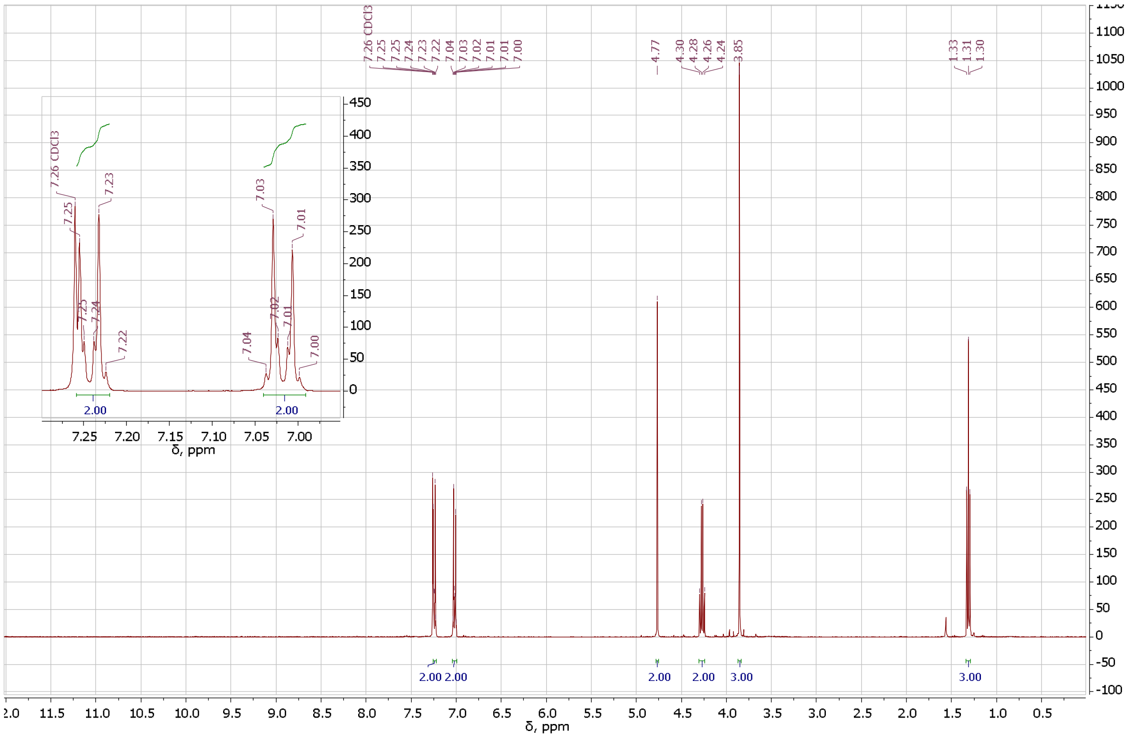

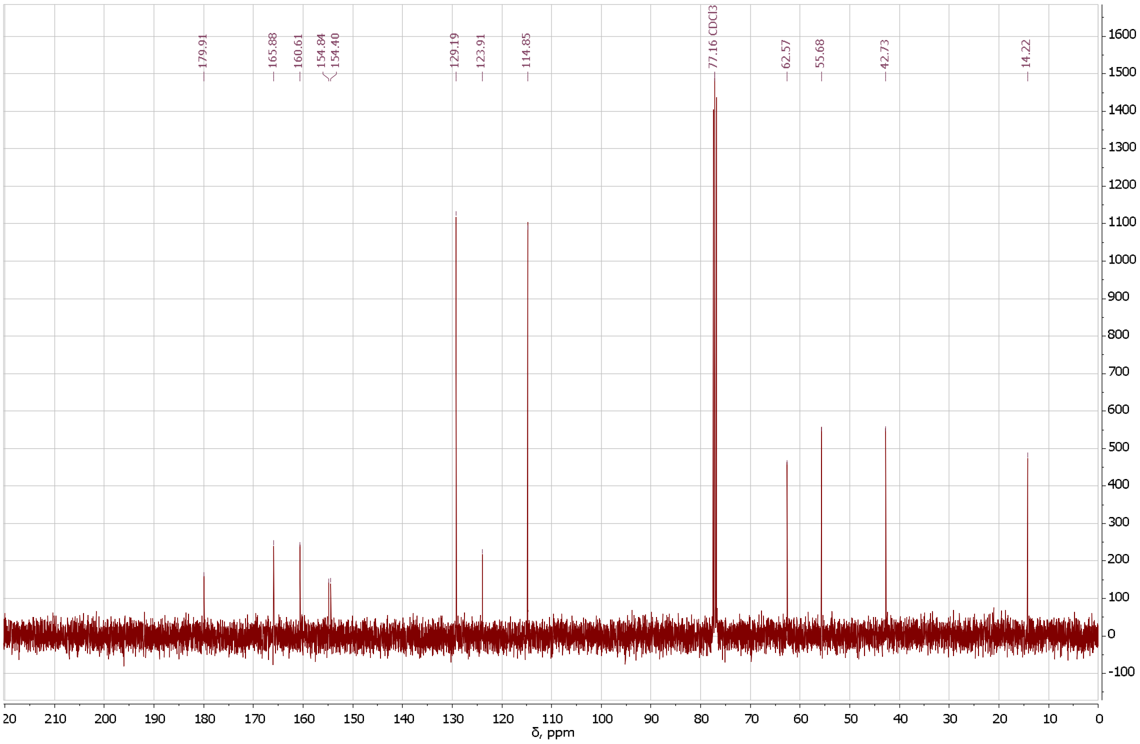


## 1-Allyl-2-thioxoimidazolidine-4,5-dione (1f)

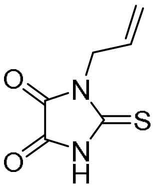


[1]H NMR (400 MHz, $CDCl_3$) δ 9.11 (br.s, 1H, NH), 5.83 (ddt, *J* = 16.4, 10.1, 6.0 Hz, 1H, CH), 5.34 – 5.27 (m, 2H, =$CH_2$), 4.54 (d, *J* = 6.0 Hz, 2H, $NCH_2$).

[13]C NMR (101 MHz, DMSO) δ 178.0, 155.7, 155.0, 129.3, 120.2, 43.7.

HRMS (ESI): calcd for $C_6H_6N_2O_2S$ $(M+Na)^+$ 193.0042, found 193.0042.

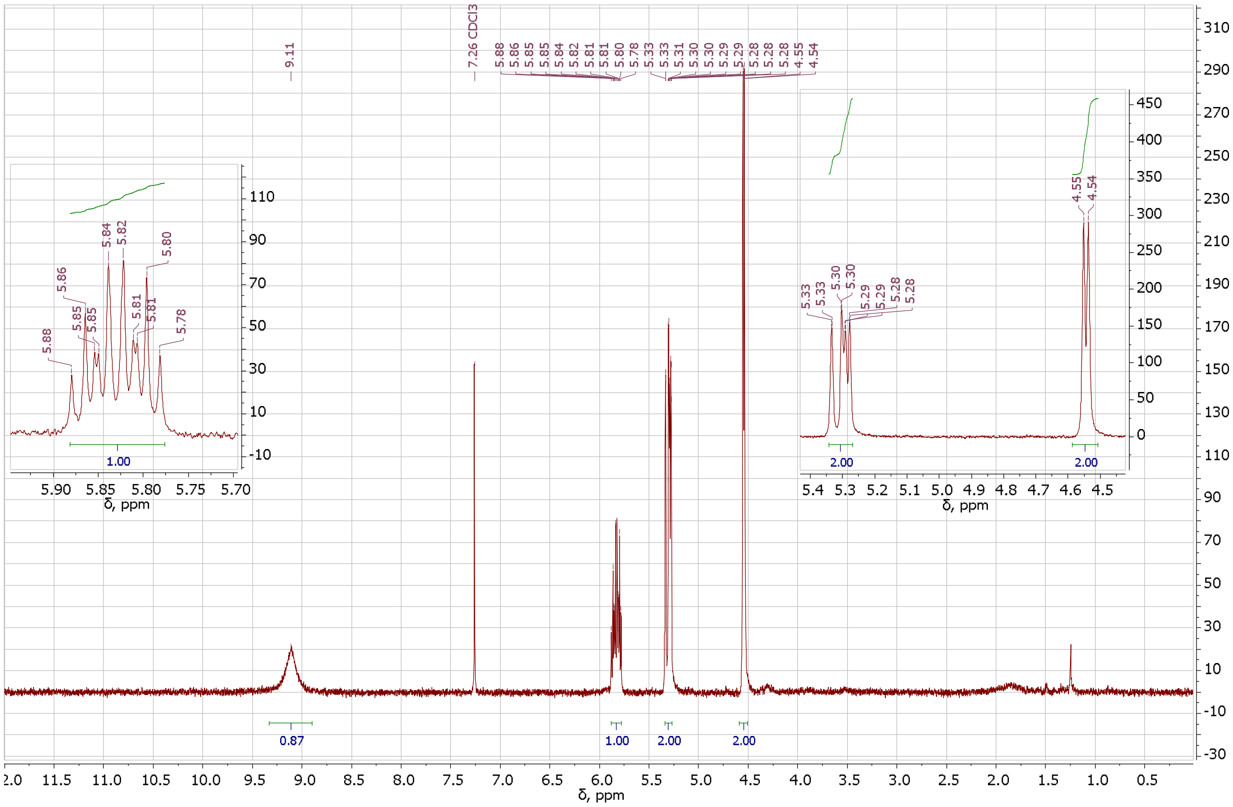

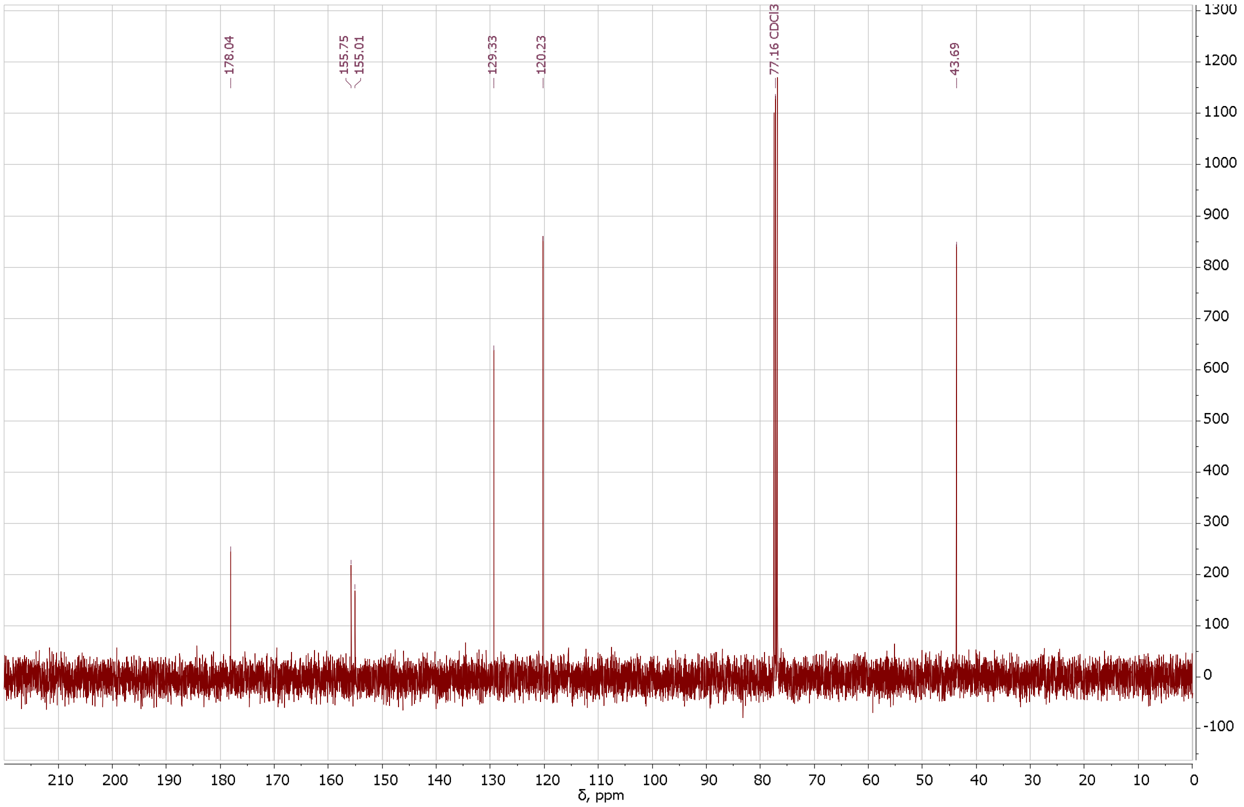


**¹H, ¹³C NMR and HRMS spectral data of 5-aryliminoimidazolidine-4,5-diones 2a-m**

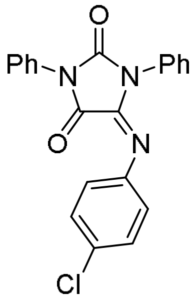


**(*E*)-5-((4-Chlorophenyl)imino)-1,3-diphenylimidazolidine-2,4-dione (2a)**

$^1$H NMR (400 MHz, $CDCl_3$) δ 7.57 – 7.51 (m, 4H, Ar), 7.50 – 7.40 (m, 6H, Ar), 7.28 (d, *J* = 8.5 Hz, 2H, Ar), 6.94 (d, *J* = 8.5 Hz, 2H, Ar).

$^{13}$C NMR (101 MHz, $CDCl_3$) δ 153.3, 152.1, 144.6, 131.4, 131.0, 130.4, 130.2, 129.6, 129.4, 129.3, 128.9, 128.9, 128.8, 127.2, 126.0, 125.9, 121.5.

HRMS (ESI): calcd for $C_{21}H_{14}ClN_3O_2$ $(M+H)^+$ 376.0847, found 376.0852.

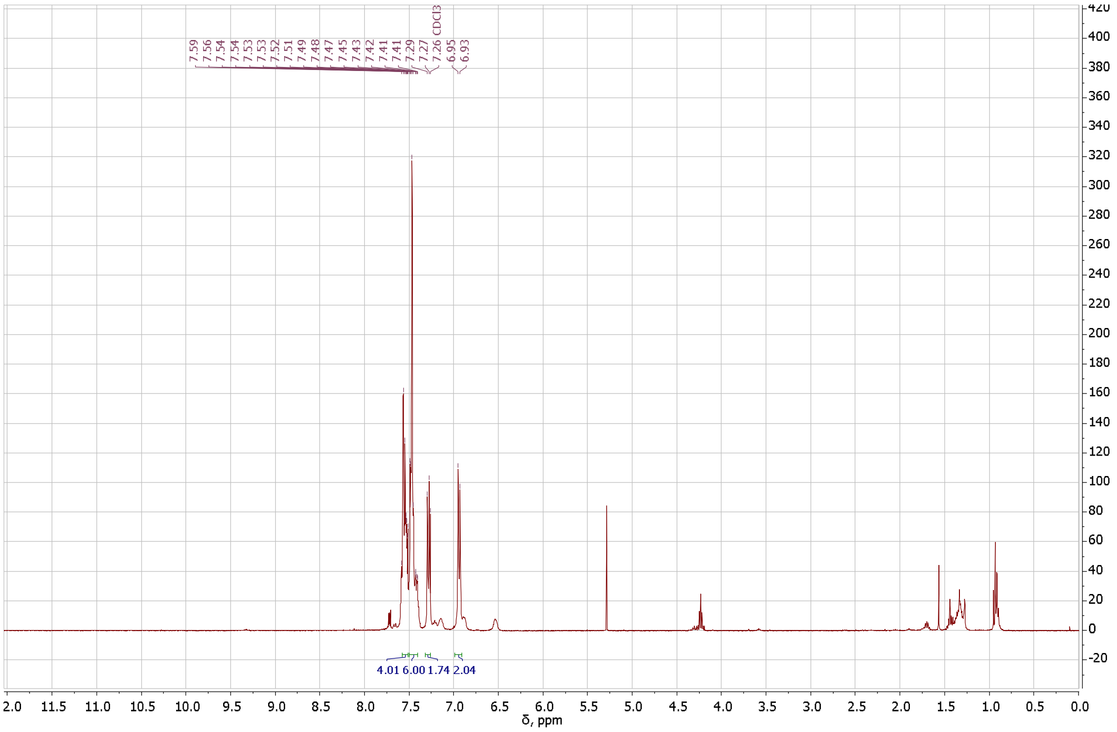


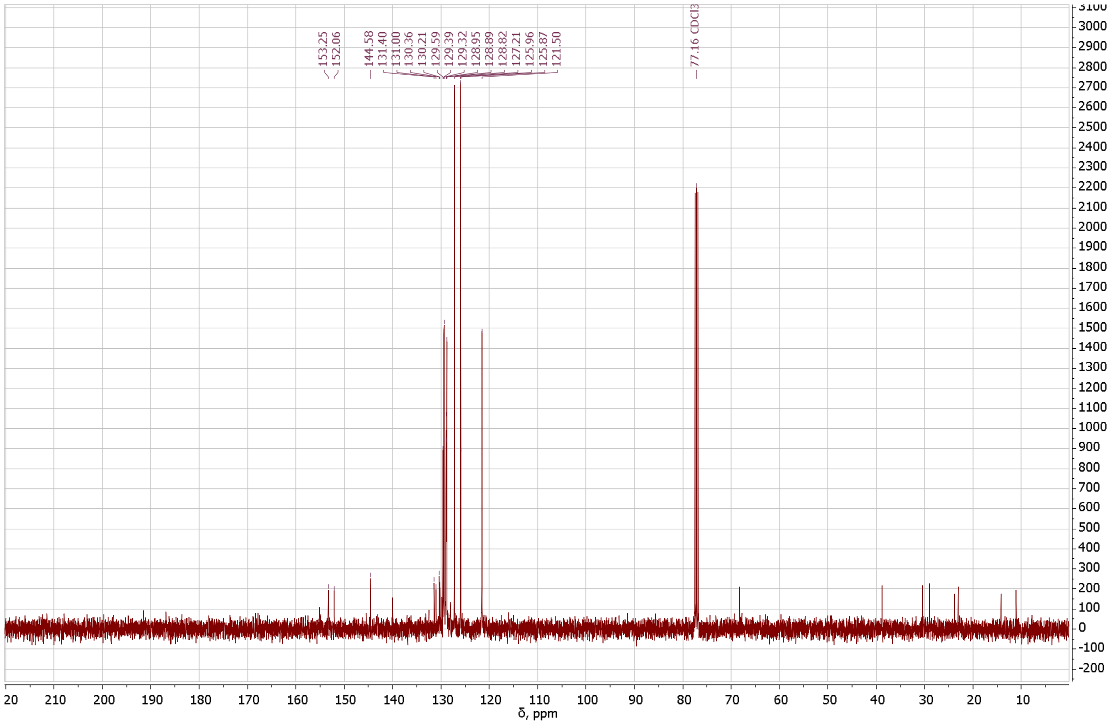


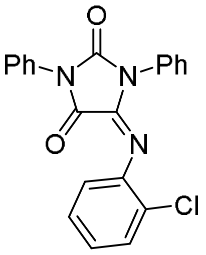


## (*E*)-5-((2-Chlorophenyl)imino)-1,3-diphenylimidazolidine-2,4-dione (2b)

1H NMR (400 MHz, $CDCl_3$) δ 7.66 (d, J = 7.9 Hz, 2H, Ar), 7.56 (t, J = 7.7 Hz, 2H, Ar), 7.47 (d, J = 4.2 Hz, 5H, Ar), 7.40 (d, J = 8.2 Hz, 2H, Ar), 7.21 (t, J = 7.6 Hz, 1H, Ar), 7.06 (t, J = 7.6 Hz, 1H, Ar), 6.95 (d, J = 8.0 Hz, 1H, Ar).

$^{13}C$ NMR (101 MHz, $CDCl_3$) δ 153.2, 152.1, 144.0, 141.5, 131.3, 130.2, 129.6, 129.4, 129.3, 128.9, 127.2, 127.2, 125.9, 125.4, 120.6.

HRMS (ESI): calcd for $C_{21}H_{14}ClN_3O_2$ $(2M+Na)^+$ 773.1441, found 773.1443.

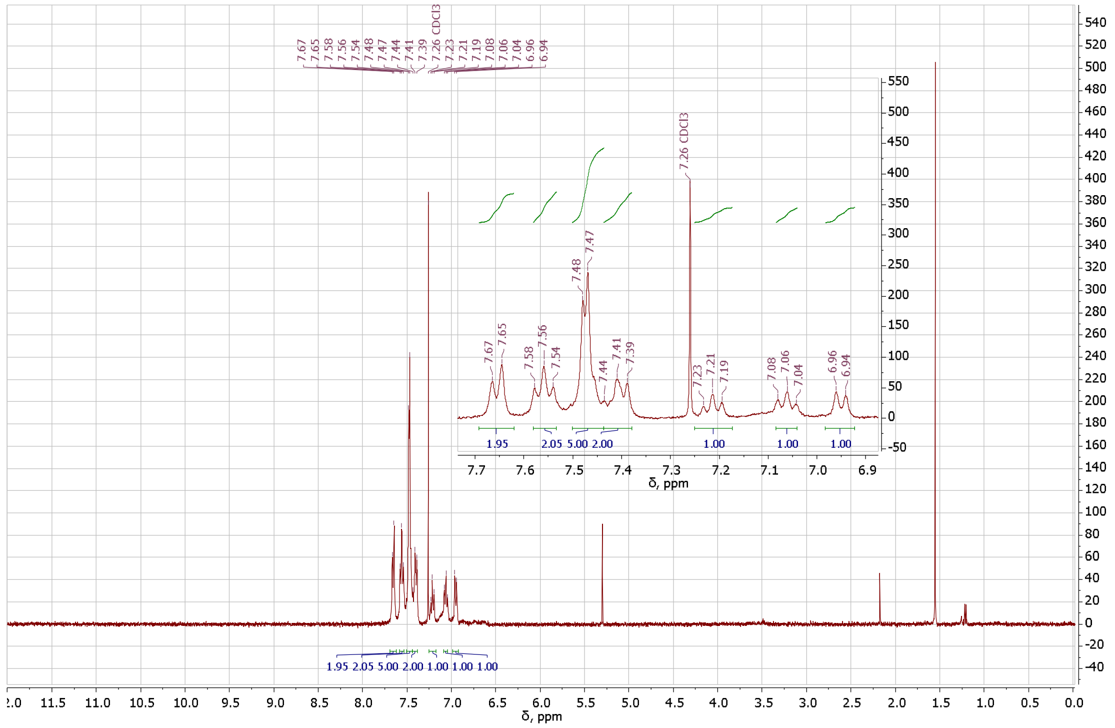


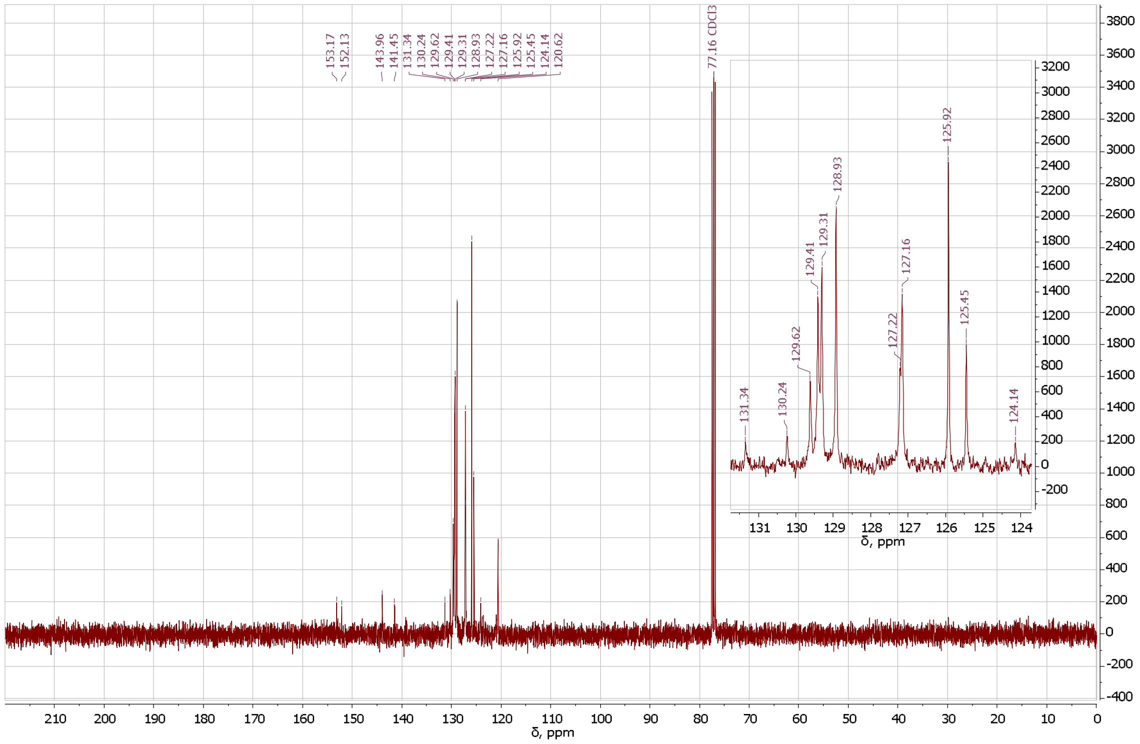


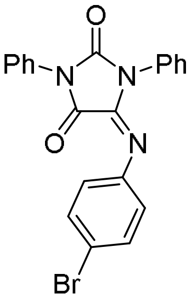


## (*E*)-5-((4-Bromophenyl)imino)-1,3-diphenylimidazolidine-2,4-dione (2c)

$^{1}$H NMR (400 MHz, $CDCl_3$) δ 7.57 – 7.53 (m, 4H, Ar), 7.51 – 7.45 (m, 5H, Ar), 7.42 (d, $J$ = 8.3 Hz, 3H, Ar), 6.87 (d, $J$ = 8.2 Hz, 2H, Ar).

$^{13}$C NMR (101 MHz, $CDCl_3$) δ 153.2, 152.0, 145.1, 139.9, 131.7, 131.4, 130.2, 129.4, 129.3, 128.9, 128.9, 127.2, 125.9, 121.8, 118.1.

HRMS (ESI): calcd for $C_{21}H_{14}BrN_3O_2$ $(M+H)^+$ 420.0342, found 420.0344.

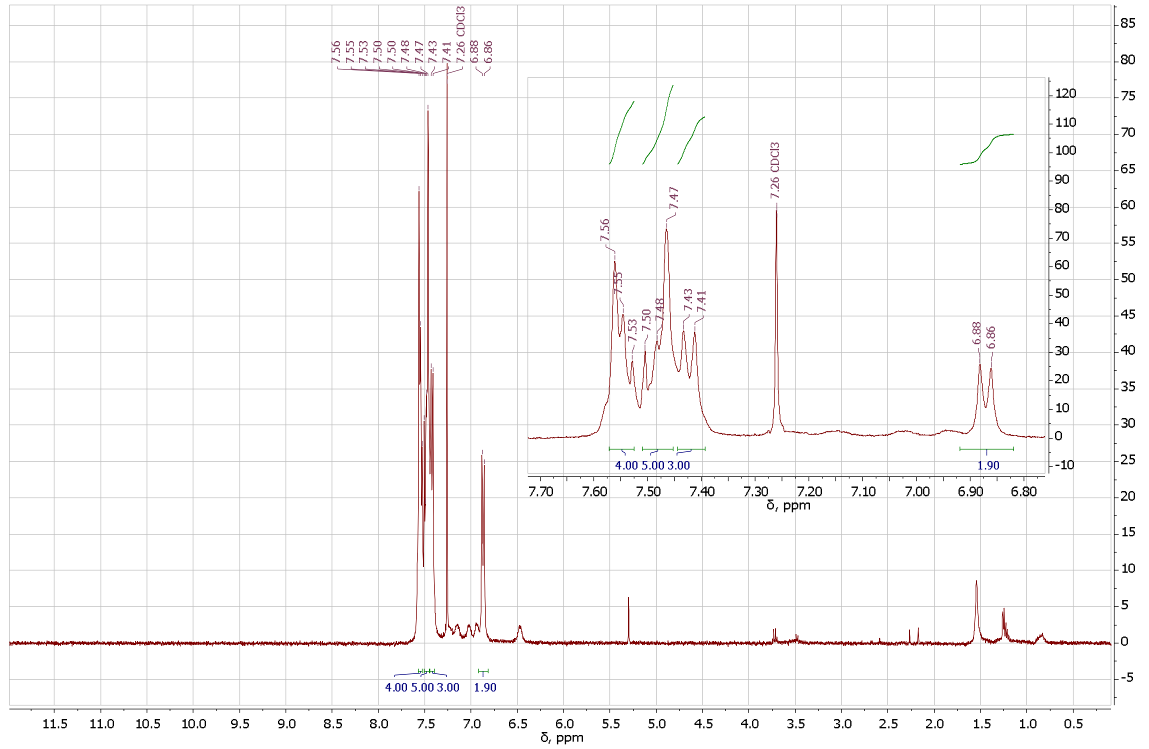


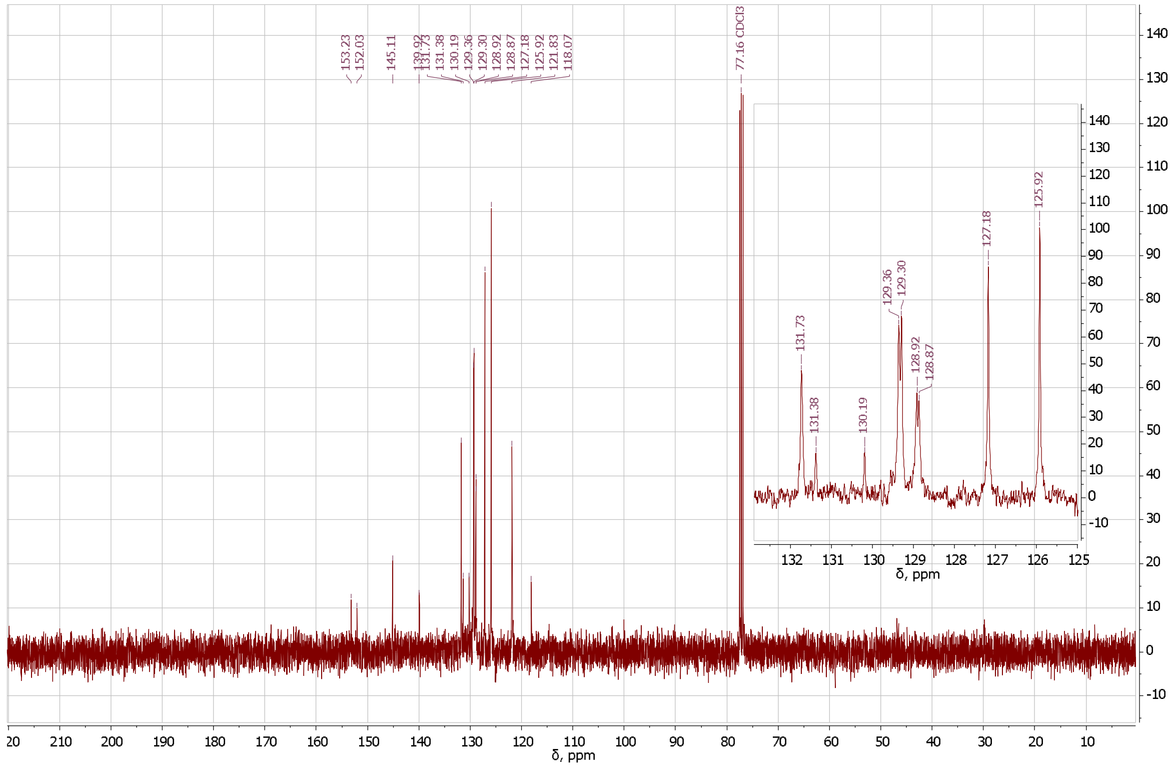


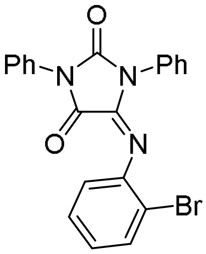


## (*E*)-5-((2-Bromophenyl)imino)-1,3-diphenylimidazolidine-2,4-dione (2d)

$^1$H NMR (400 MHz, $CDCl_3$) δ 7.73 (d, *J* = 7.9 Hz, 2H, Ar), 7.63 (d, *J* = 8.0 Hz, 1H, Ar), 7.58 (t, *J* = 7.8 Hz, 2H, Ar), 7.53 – 7.45 (m, 5H, Ar), 7.43 – 7.39 (m, 1H, Ar), 7.29 (t, *J* = 7.6 Hz, 1H, Ar), 7.06 – 6.97 (m, 2H, Ar).

$^{13}$C NMR (101 MHz, $CDCl_3$) δ 153.0, 145.3, 132.7, 131.3, 130.2, 129.4, 129.3, 128.9, 127.8, 127.2, 125.9, 125.7, 120.4, 114.1.

HRMS (ESI): calcd for $C_{21}H_{14}BrN_3O_2$ $(M+Na)^+$ 442.0162, found 442.0167.

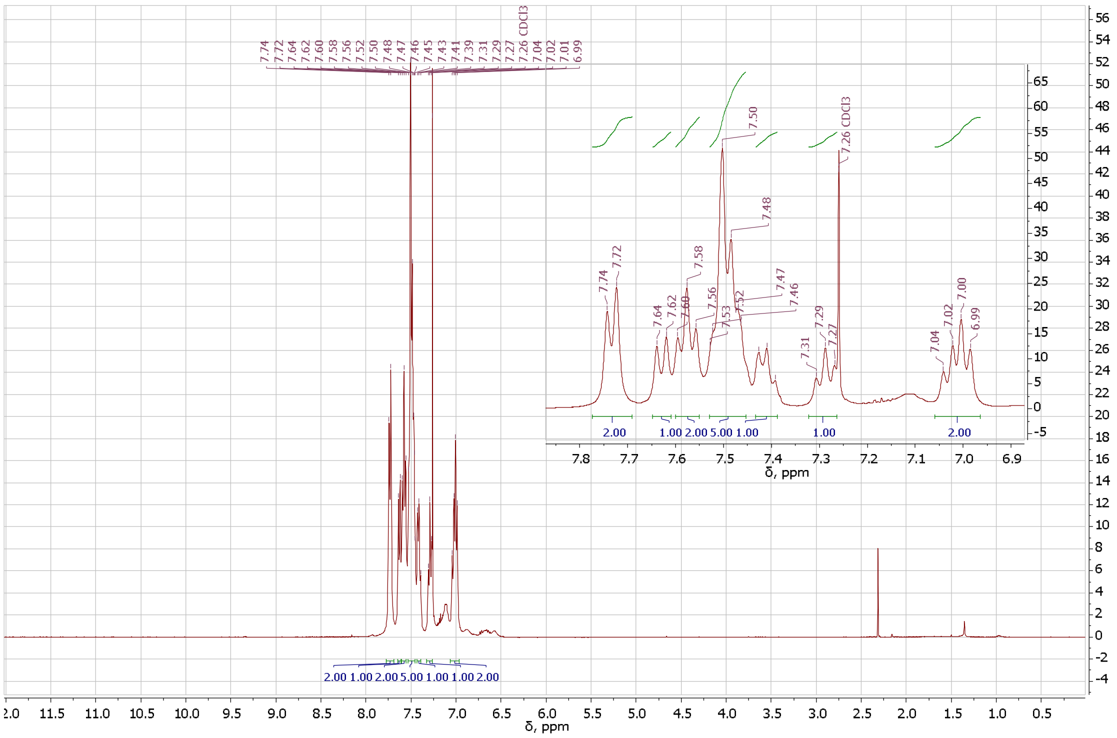


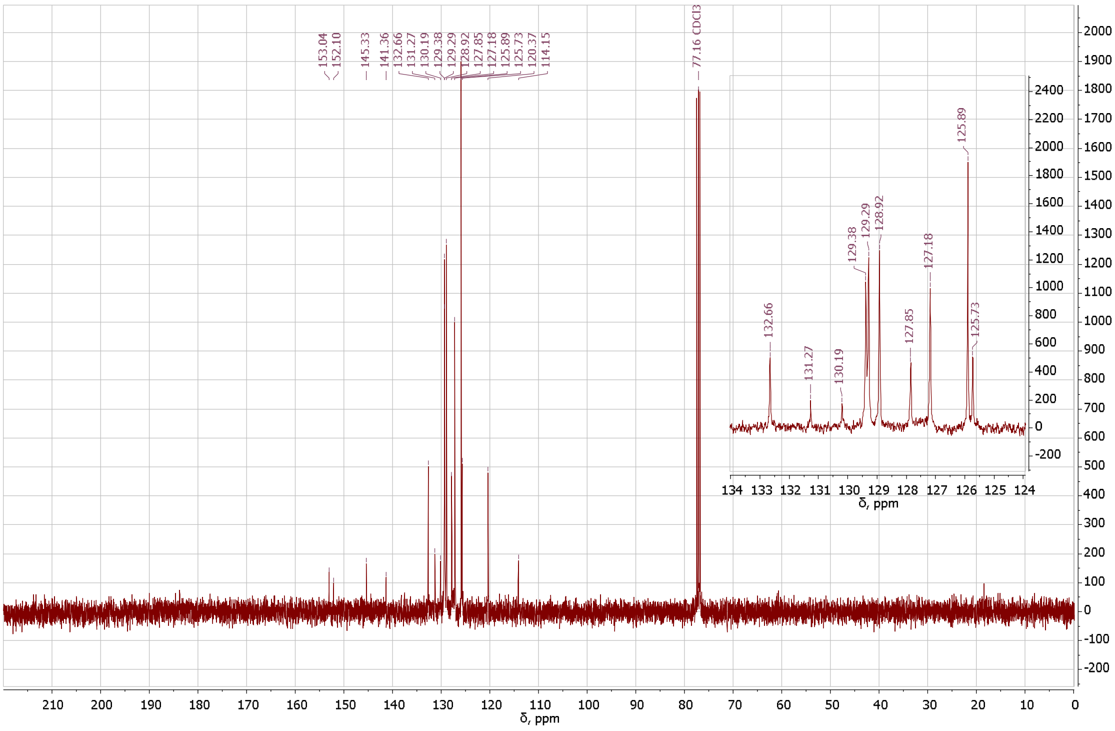


## (*E*)-1,3-Diphenyl-5-(p-tolylimino)imidazolidine-2,4-dione (2e)

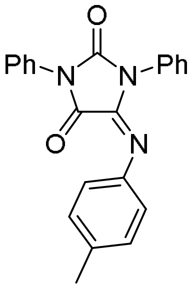


$^{1}$H NMR (400 MHz, $CDCl_3$) δ 7.61 – 7.52 (m, 4H, Ar), 7.52 – 7.44 (m, 5H, Ar), 7.43 – 7.38 (m, 1H, Ar), 7.13 (d, $J$ = 7.9 Hz, 2H, Ar), 6.92 (d, $J$ = 7.9 Hz, 2H, Ar), 2.33 (s, 3H, $CH_3$).

$^{13}$C NMR (101 MHz, $CDCl_3$) δ 153.2, 143.4, 139.4, 134.8, 130.4, 129.3, 129.2, 128.8, 128.8, 128.7, 127.3, 126.1, 120.2, 120.1, 120.1, 21.1.

HRMS (ESI): calcd for $C_{22}H_{17}N_3O_2$ $(M+H)^+$ 356,1394, found 356.1396.

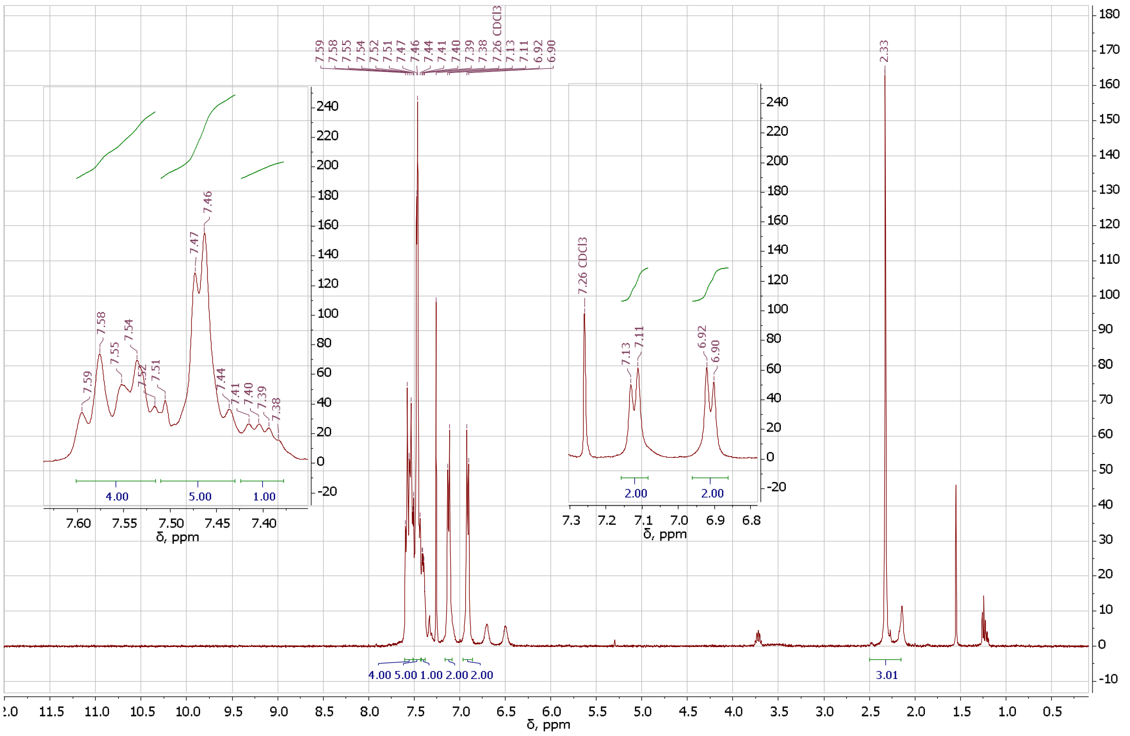


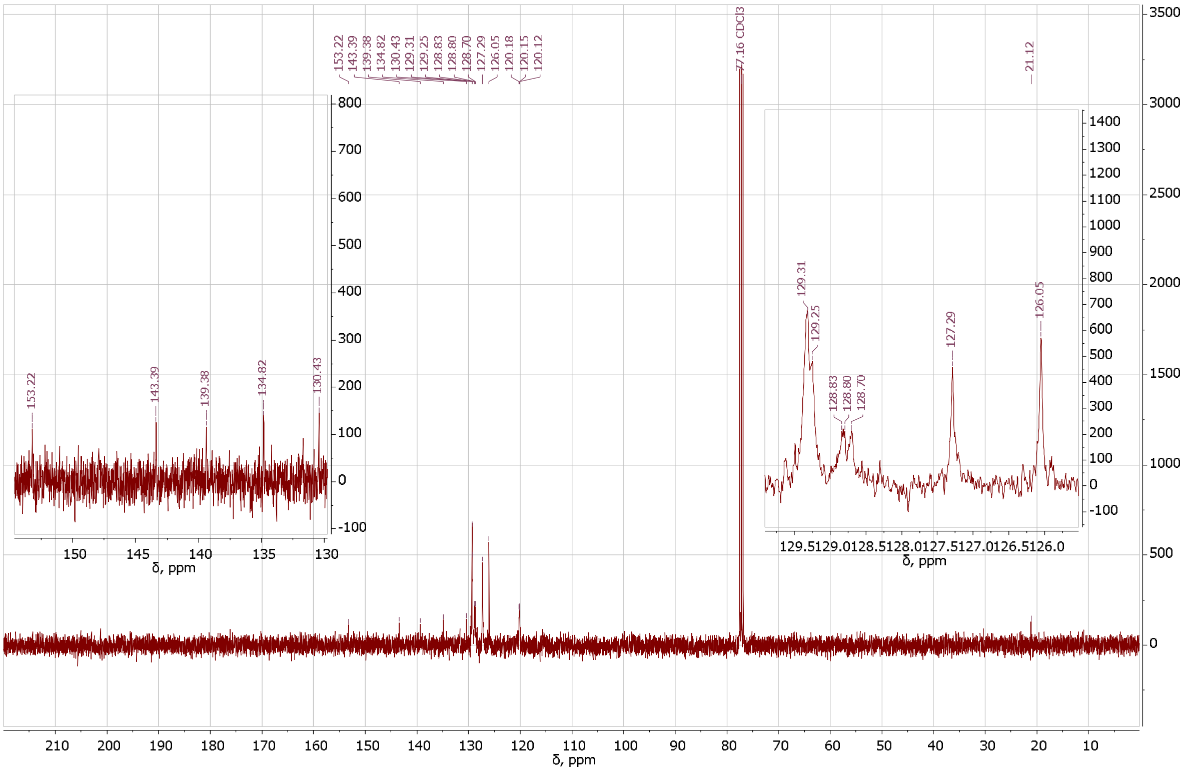


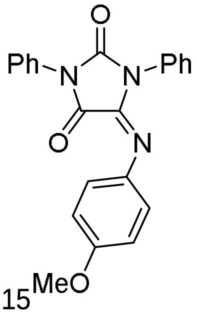


## (*E*)-5-((4-Methoxyphenyl)imino)-1,3-diphenylimidazolidine-2,4-dione (2f)

$^1$H NMR (400 MHz, DMSO-d6) δ 7.58 – 7.54 (m, 4H, Ar), 7.52 (d, *J* = 7.6 Hz, 2H, Ar), 7.48 – 7.44 (m, 4H, Ar), 7.02 (d, *J* = 8.7 Hz, 2H, Ar), 6.84 (d, *J* = 8.8 Hz, 2H, Ar), 3.72 (s, 3H, $OCH_3$).

$^{13}C$ NMR (101 MHz, DMSO-d6) δ 156.4, 153.9, 152.4, 140.7, 139.0, 132.5, 131.0, 129.0, 128.9, 128.5, 128.2, 127.9, 127.0, 122.3, 113.5, 55.2.

HRMS (ESI): calcd for $C_{22}H_{17}N_3O_3$ $(M+H)^+$ 372.1343, found 372.1347.

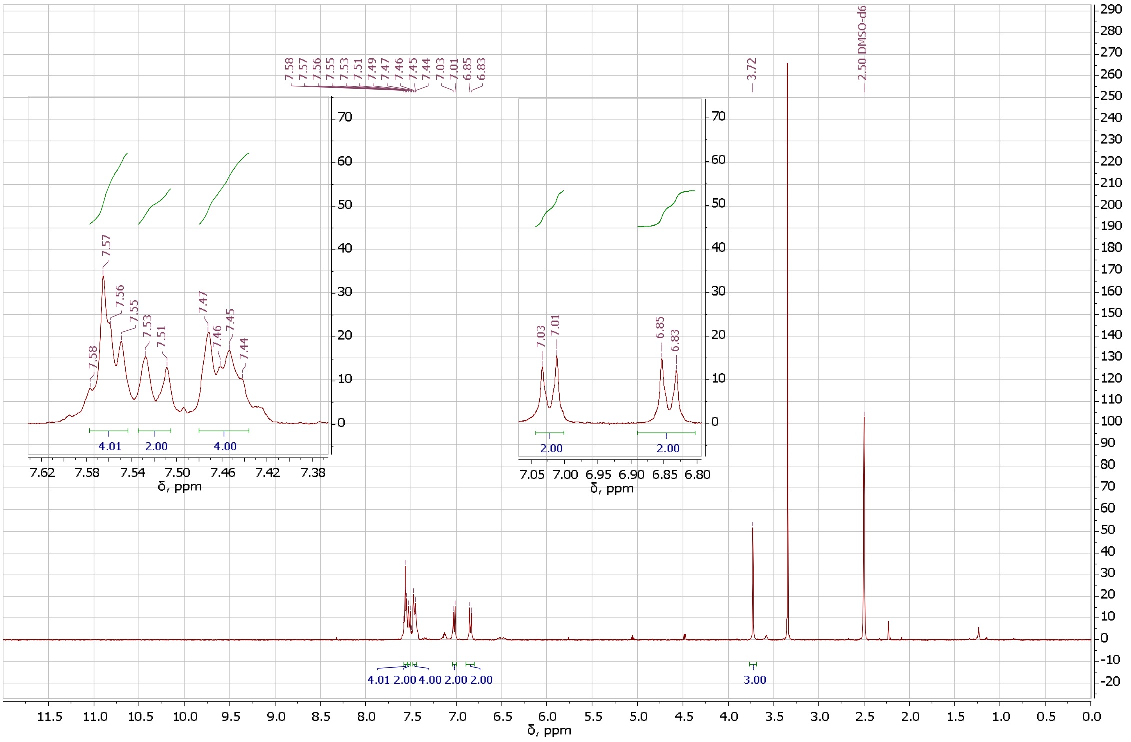


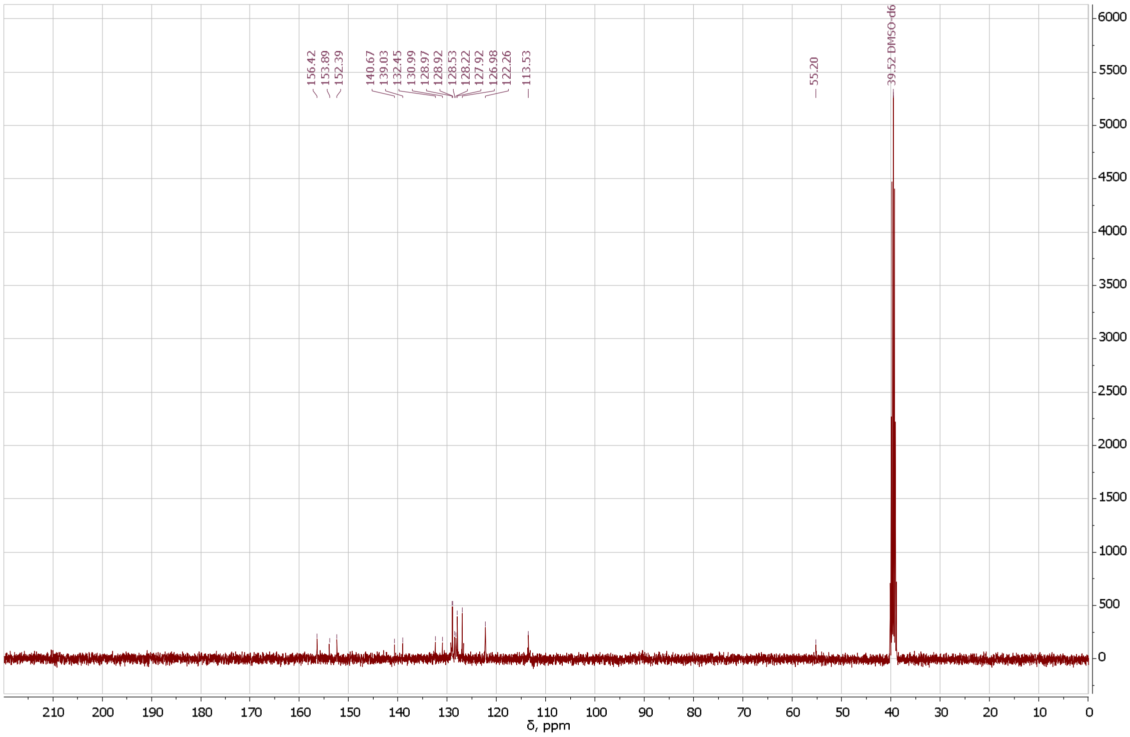


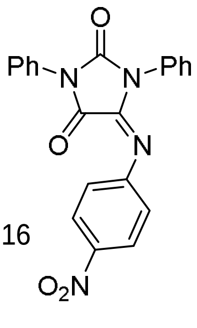


## (*E*)-5-((4-Nitrophenyl)imino)-1,3-diphenylimidazolidine-2,4-dione (2g)

$^1H$ NMR (400 MHz, $CDCl_3$) δ 8.19 (s, 2H, Ar), 7.57 (m, 3H, Ar), 7.52 – 7.38 (m, 7H, Ar), 7.03 (s, 2H, Ar).

$^{13}C$ NMR (101 MHz, $CDCl_3$) δ 153.4, 152.8, 152.0, 144.5, 140.3, 131.1, 130.0, 129.5, 129.4, 129.2, 129.1, 127.1, 125.8, 124.8, 120.2.

HRMS (ESI): calcd for $C_{21}H_{14}N_4O_4$ $(M+H)^+$ 409.0907, found 409.0911.

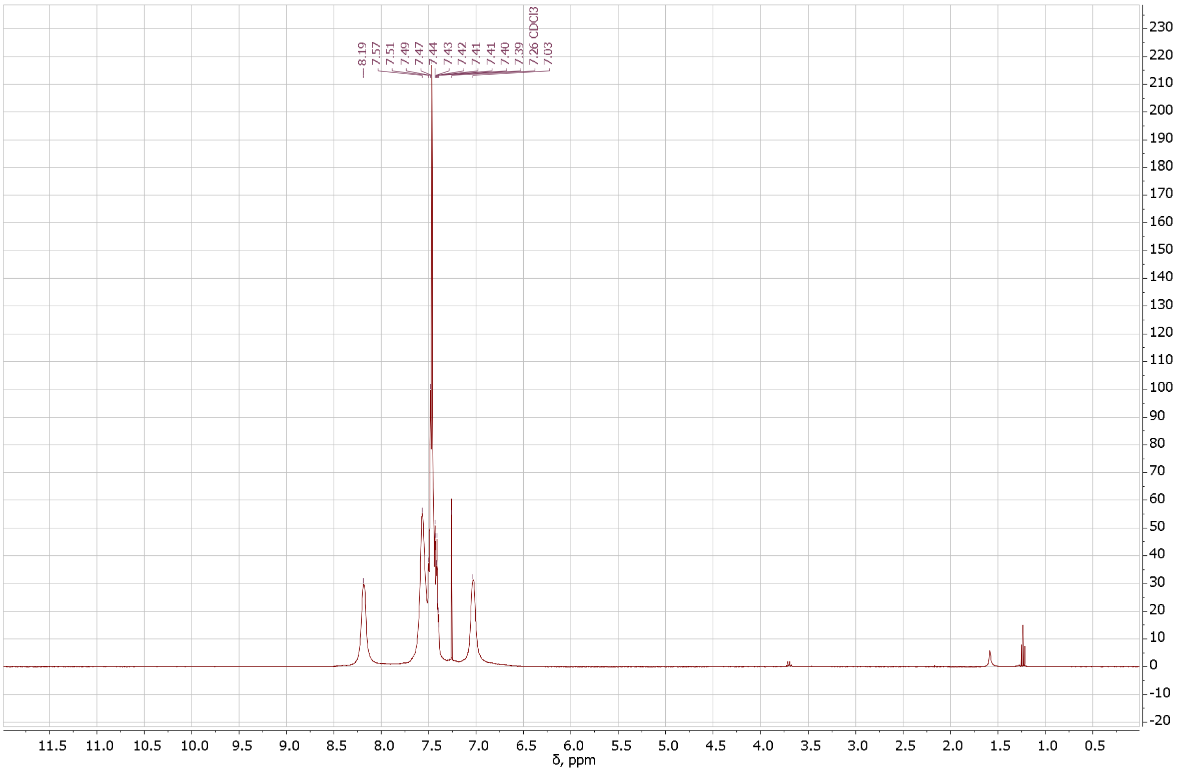


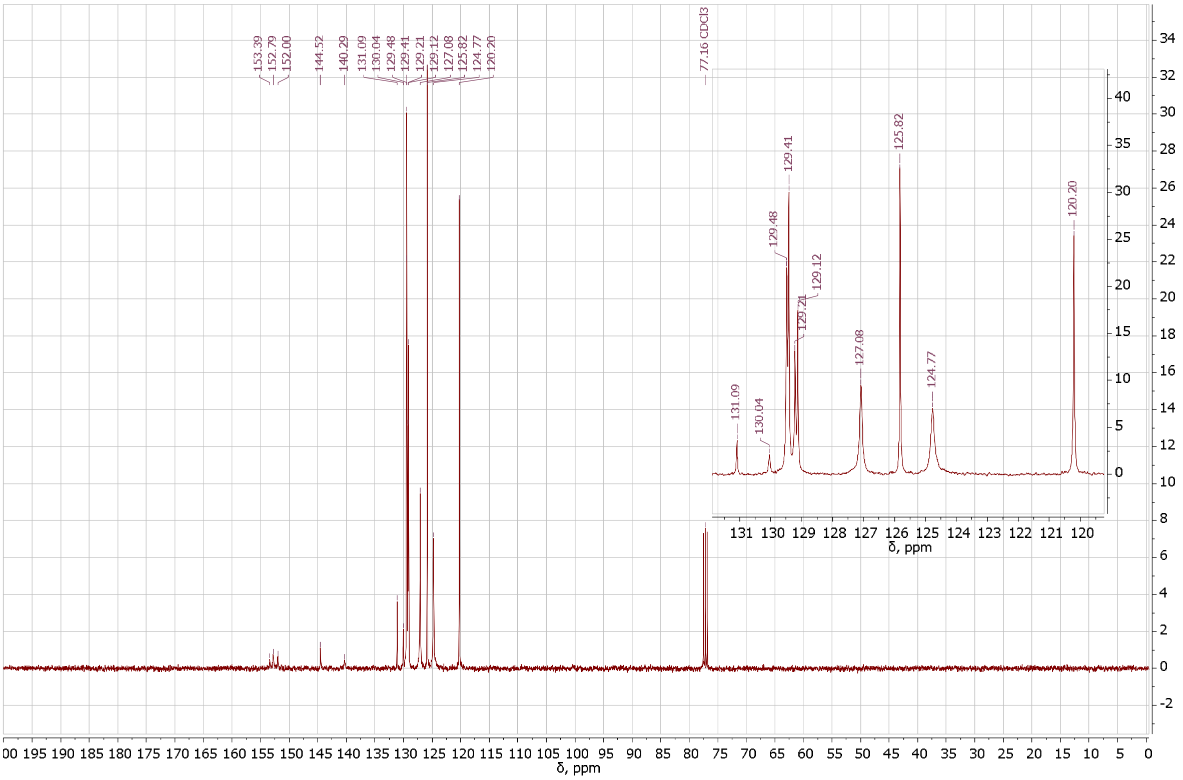


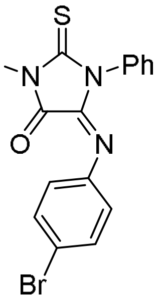


17

**(*E*)-5-((4-bromophenyl)imino)-1,3-diphenyl-2-thioxoimidazolidin-4-one (2h)**

$^1$H NMR (400 MHz, $CDCl_3$) δ 7.60 – 7.55 (m, 2H, Ar), 7.50 (q, *J* = 9.2, 8.3 Hz, 6H, Ar), 7.43 – 7.36 (m, 4H, Ar), 6.91 (d, *J* = 8.3 Hz, 2H, Ar).

$^{13}$C NMR (101 MHz, $CDCl_3$) δ 179.2, 153.1, 144.5, 133.8, 132.0, 131.7, 129.8, 129.7, 129.5, 129.4, 128.9, 128.3, 125.4, 122.4, 118.7.

HRMS (ESI): calcd for $C_{22}H_{17}N_3O_2S$ $(2M+Na)^+$ 797.1975, found 797.1986.

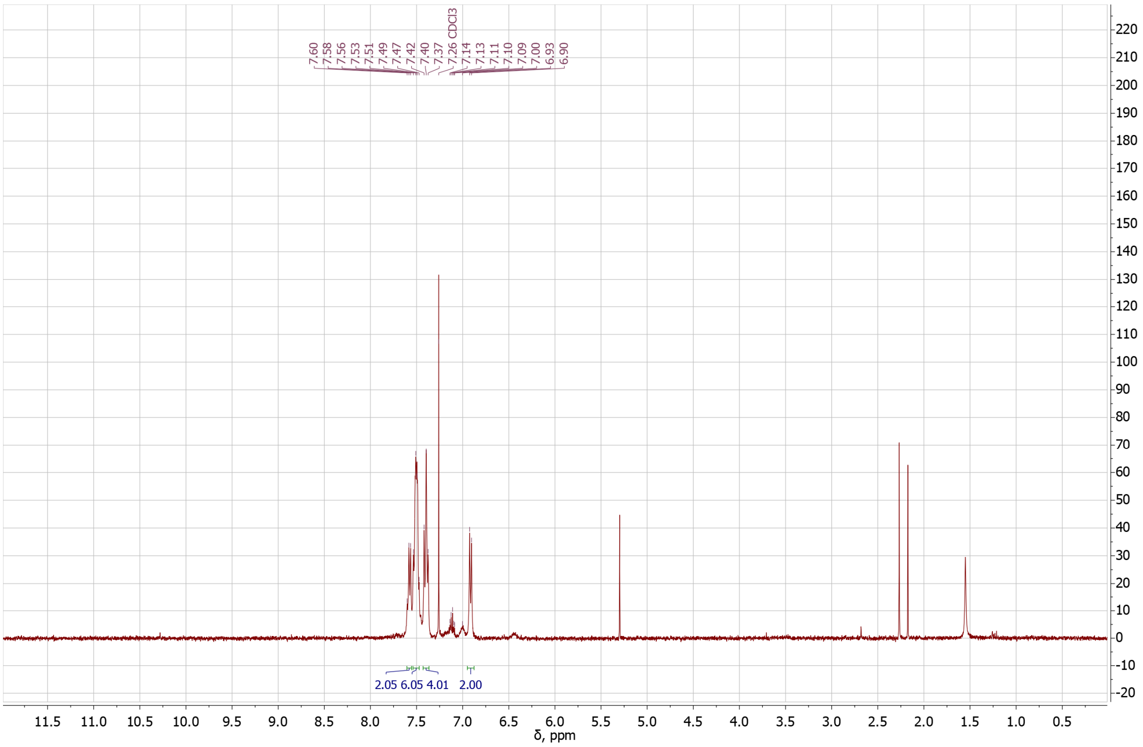


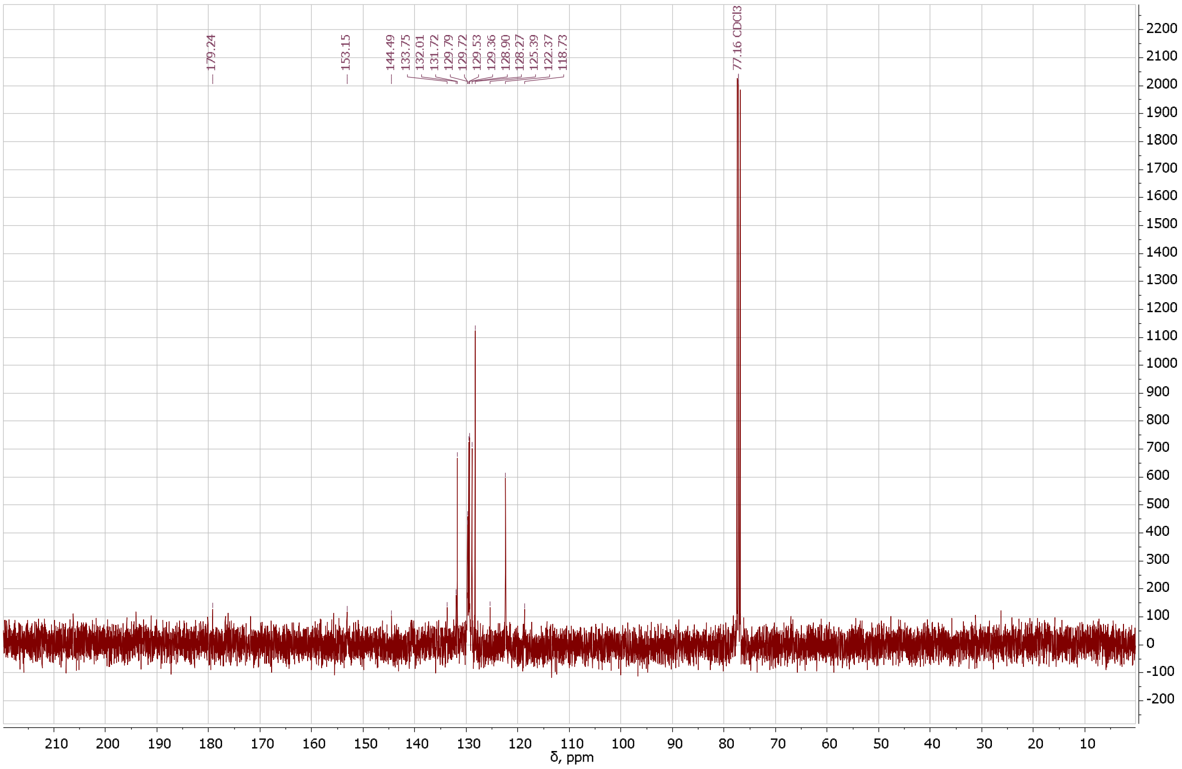

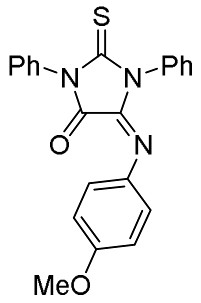


## (*E*)-5-((4-Methoxyphenyl)imino)-1,3-diphenyl-2-thioxoimidazolidin-4-one (2i)

[1]H NMR (400 MHz, $CDCl_3$) δ 7.58 – 7.53 (m, 2H, Ar), 7.53 – 7.46 (m, 6H, Ar), 7.40 (d, *J* = 7.4 Hz, 2H, Ar), 7.17 (d, *J* = 6.5 Hz, 2H, Ar), 6.83 (d, *J* = 6.6 Hz, 2H, Ar), 3.78 (s, 3H, $OCH_3$).

[13]C NMR (101 MHz, $CDCl_3$) δ 178.9, 158.3, 153.1, 139.3, 137.6, 134.2, 132.3, 129.6, 129.3, 129.0, 128.4, 123.9, 113.7, 55.5.

HRMS (ESI): calcd for $(M+H)^+$ 388.1114, found 388.1122.

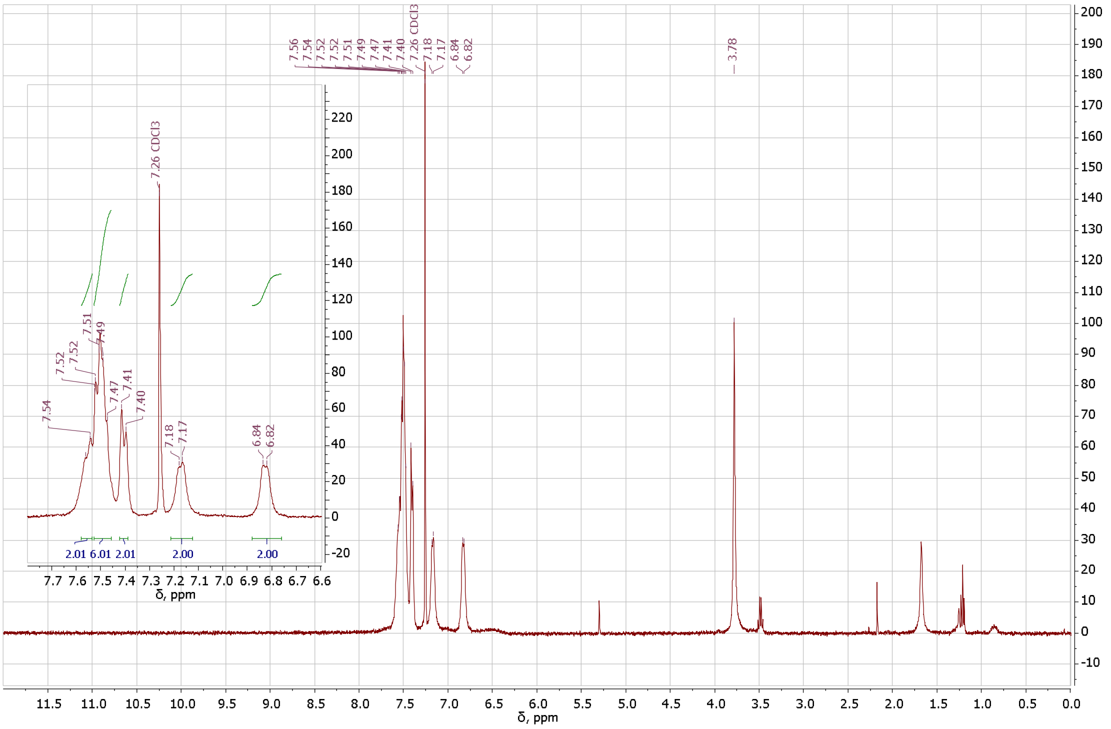

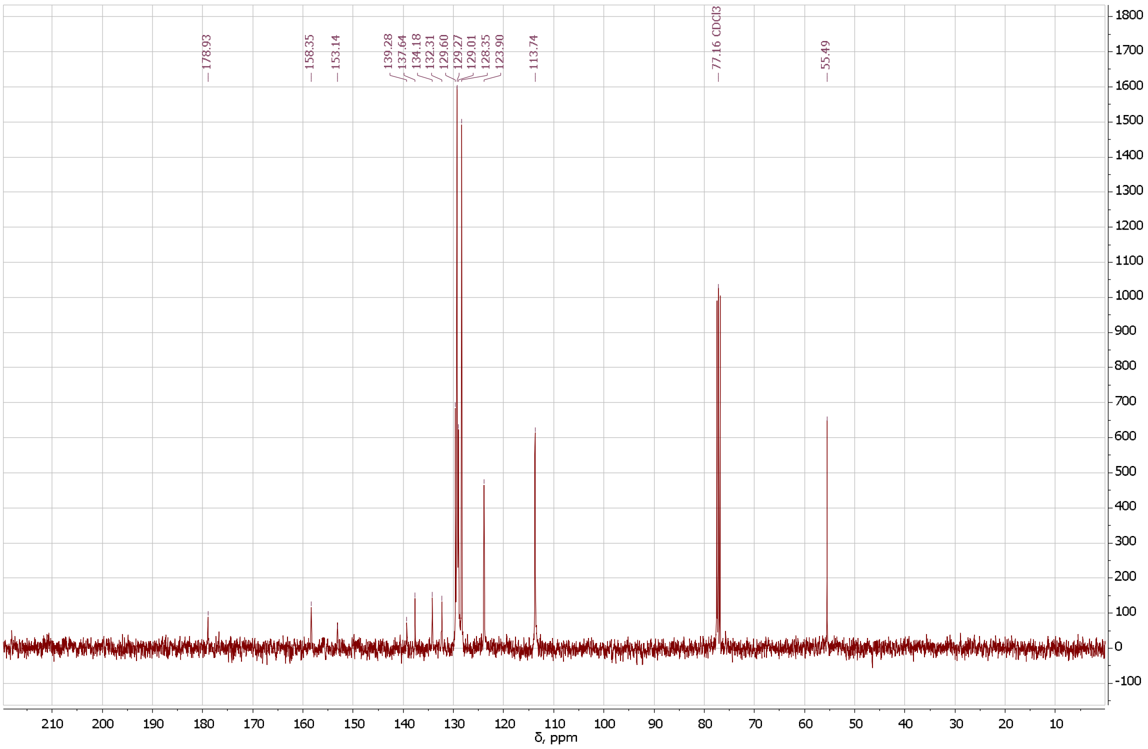


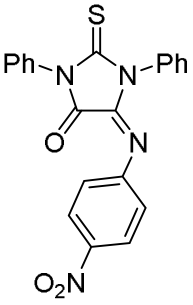

**(*E*)-5-((4-Nitrophenyl)imino)-1,3-diphenyl-2-thioxoimidazolidin-4-one (2j)**

$^{1}$H NMR (400 MHz, $CDCl_3$) δ 8.19 (s, 2H, Ar), 7.64 – 7.46 (m, 8H, Ar), 7.41 (d, *J* = 7.4 Hz, 2H, Ar), 7.08 (s, 2H, Ar).

$^{13}$C NMR (101 MHz, $CDCl_3$) δ 179.2, 152.3, 144.6, 141.1, 133.3, 131.8, 129.8, 129.5, 129.4, 128.7, 128.1, 124.6, 120.4.

HRMS (ESI): calcd for $C_{21}H_{14}N_4O_3S$ $(M+H)^+$ 403.0859, found 403.0864.

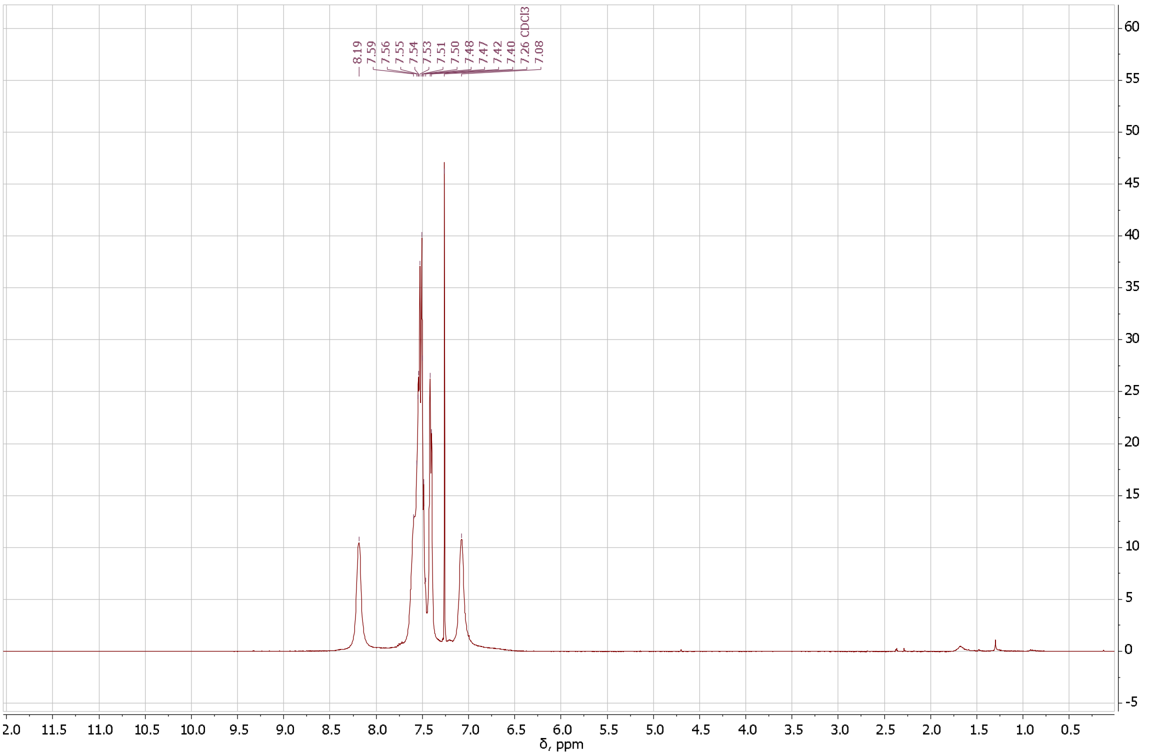

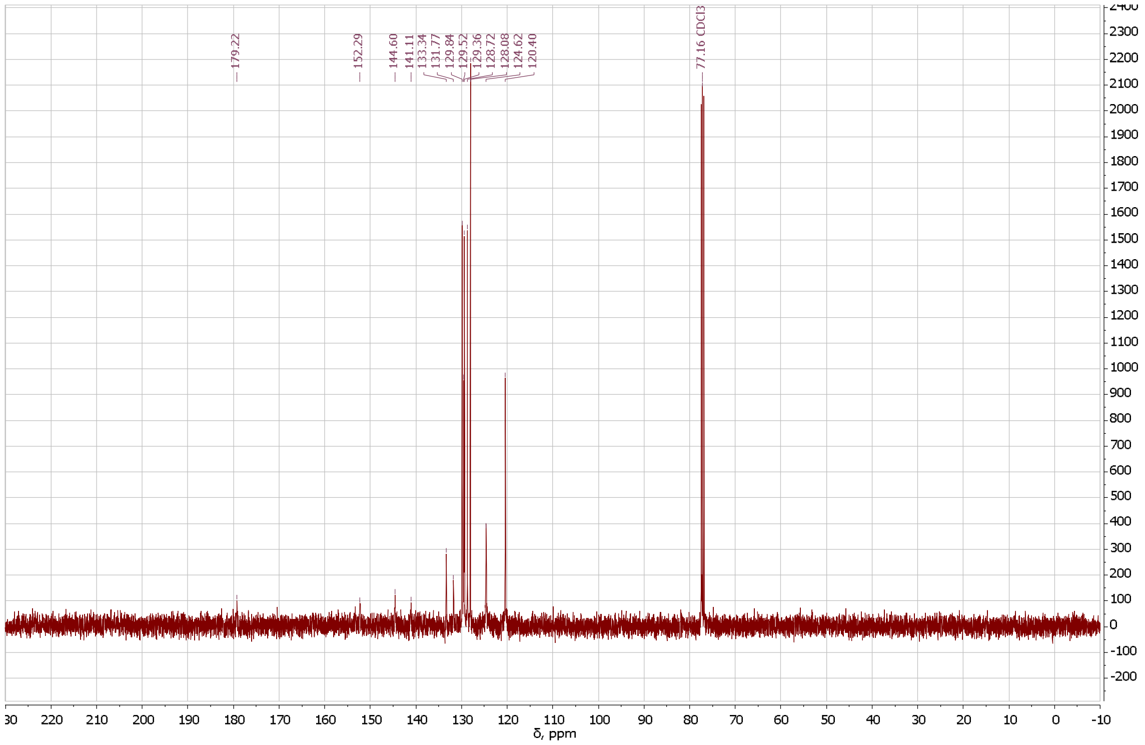


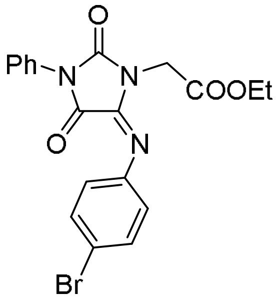


**Ethyl (*E*)-2-(5-((4-bromophenyl)imino)-2,4-dioxo-3-phenylimidazolidin-1-yl)acetate (2k)**

$^{1}$H NMR (400 MHz, $CDCl_3$) δ 7.48 – 7.43 (m, 3H, Ar), 7.43 – 7.37 (m, 4H, Ar), 6.92 – 6.87 (m, 2H, Ar), 4.59 (s, 2H, $NCH_2$), 4.29 (q, *J* = 7.2 Hz, 2H, $COOCH_2$), 1.33 (t, *J* = 7.1 Hz, 3H, $CH_3$).

$^{13}$C NMR (101 MHz, $CDCl_3$) δ 166.8, 153.5, 152.6, 144.6, 139.3, 131.8, 130.2, 129.3, 128.9, 125.9, 122.3, 118.5, 62.3, 40.7, 14.3.

HRMS (ESI): calcd for $C_{19}H_{16}BrN_3O_4$ $(M+Na)^+$ 452.0216, found 452.0222.

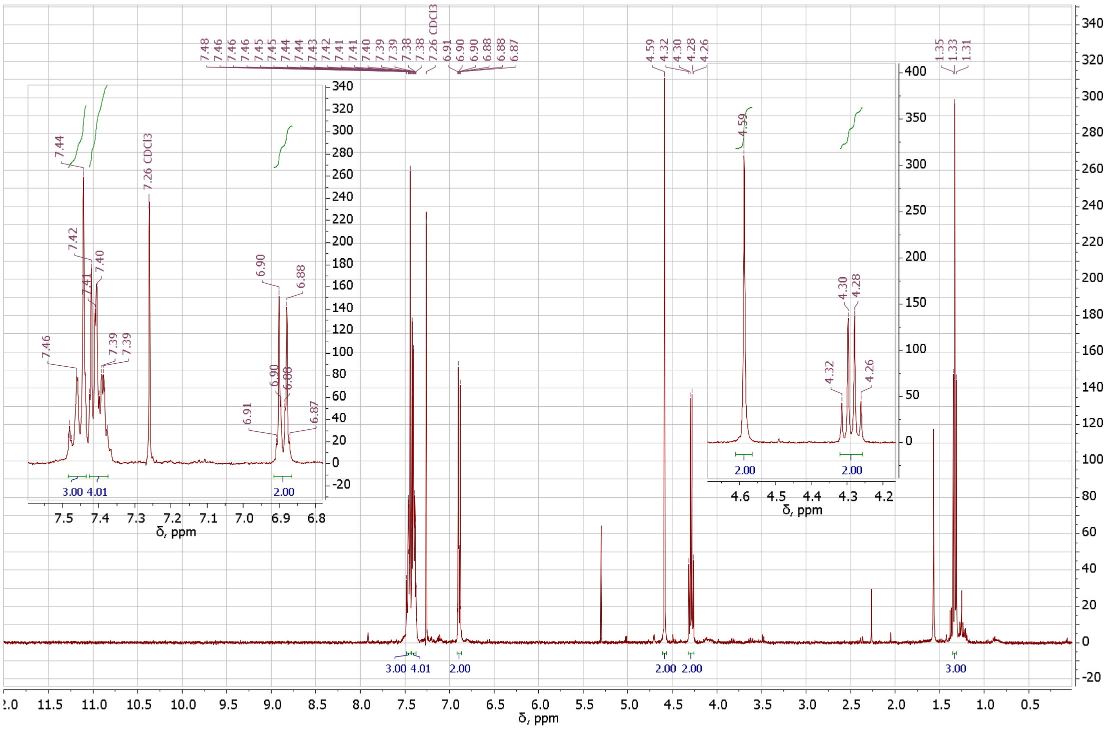


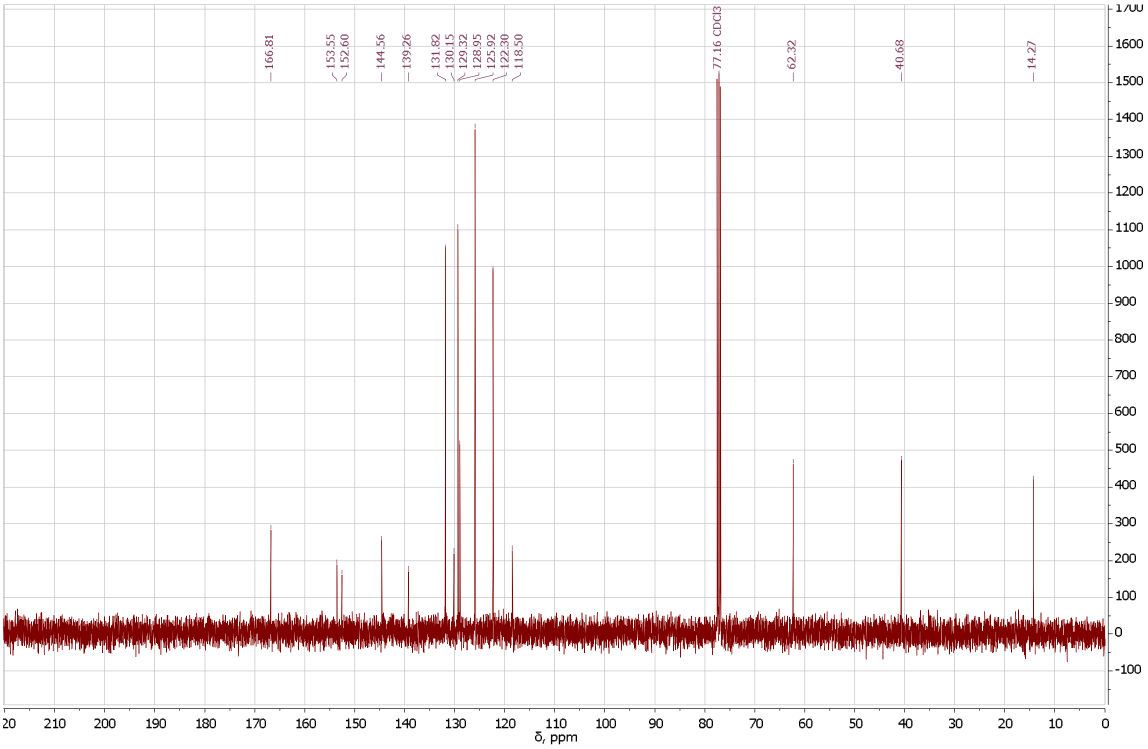


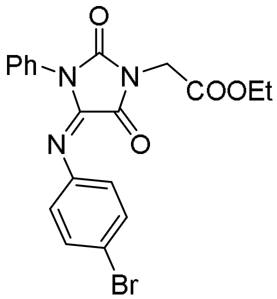


**Ethyl (*E*)-2-(4-((4-bromophenyl)imino)-2,5-dioxo-3-phenylimidazolidin-1-yl)acetate (2l)**

$^{1}$H NMR (400 MHz, $CDCl_3$) δ 7.54 – 7.48 (m, 4H, Ar), 7.46 – 7.39 (m, 3H, Ar), 6.84 (d, $J$ = 8.5 Hz, 2H, Ar), 4.38 (s, 2H, $NCH_2$), 4.25 (q, $J$ = 7.1 Hz, 2H, $COOCH_2$), 1.30 (t, $J$ = 7.1 Hz, 3H, $CH_3$).

$^{13}$C NMR (101 MHz, $CDCl_3$) δ 166.4, 153.9, 152.4, 144.9, 140.3, 131.8, 131.3, 129.4, 128.9, 127.2, 122.0, 118.3, 62.5, 39.7, 14.2.

HRMS (ESI): calcd for $C_{19}H_{16}BrN_3O_4$ $(M+Na)^+$ 452.0216, found 452.0220.

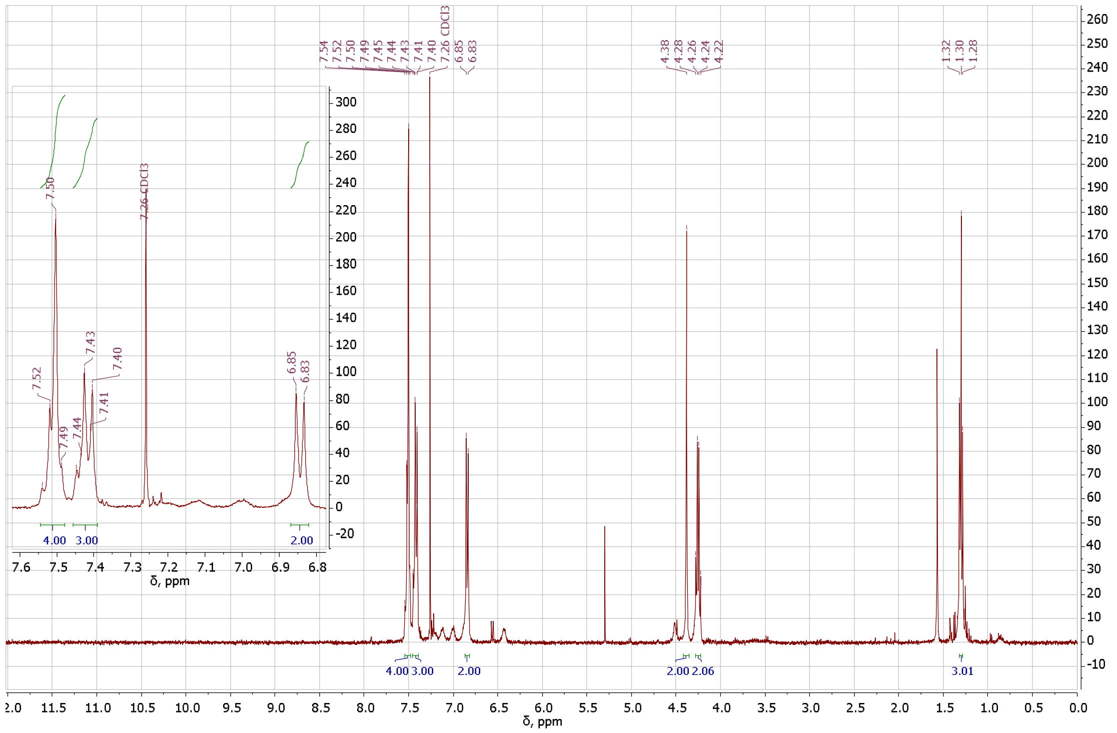


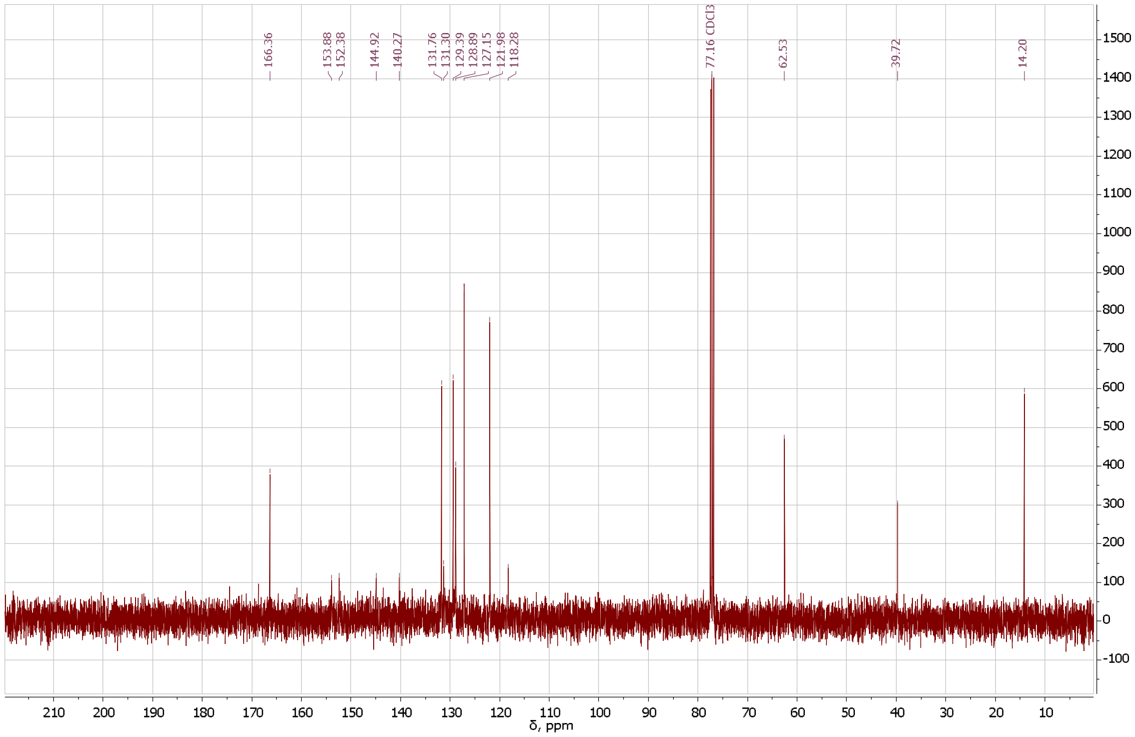


## $^1$H, $^{13}$C NMR and HRMS spectral data of 4-thia-1,2,6,9-tetraazaspiro[4.4]non-2-ene-7,8-diones 4a-g

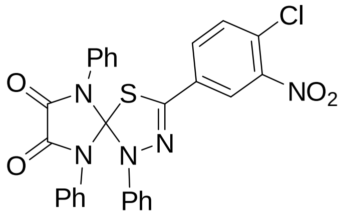


### 3-(4-Chloro-3-nitrophenyl)-1,6,9-triphenyl-4-thia-1,2,6,9-tetraazaspiro[4.4]non-2-ene-7,8-dione (4a)

$^1$H NMR (400 MHz, DMSO-d6) δ 8.12 (dd, $J$ = 1.9, 0.7 Hz, 1H, Ar), 7.72 – 7.66 (m, 2H, Ar), 7.52 (dd, $J$ = 8.8, 7.3 Hz, 2H, Ar), 7.42 (m, 8H,

Ar), 7.27 – 7.20 (m, 5H, Ar).

[13]C NMR (101 MHz, DMSO-d6) δ 156.3, 148.1, 140.5, 139.4, 132.8, 132.3, 130.1, 130.1, 129.8, 129.5, 129.4, 127.7, 125.8, 124.3, 121.9, 117.4, 112.1.

HRMS (ESI): calcd for $C_{28}H_{18}ClN_5O_4S$ $(M+H)^+$ 578.0660, found 578.0665.

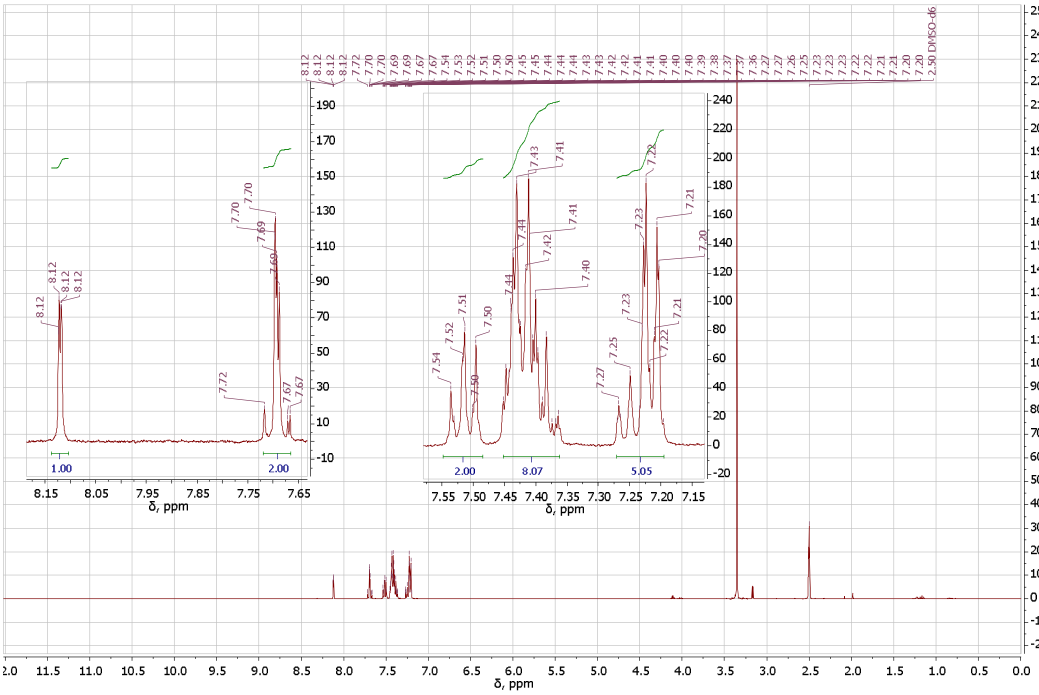


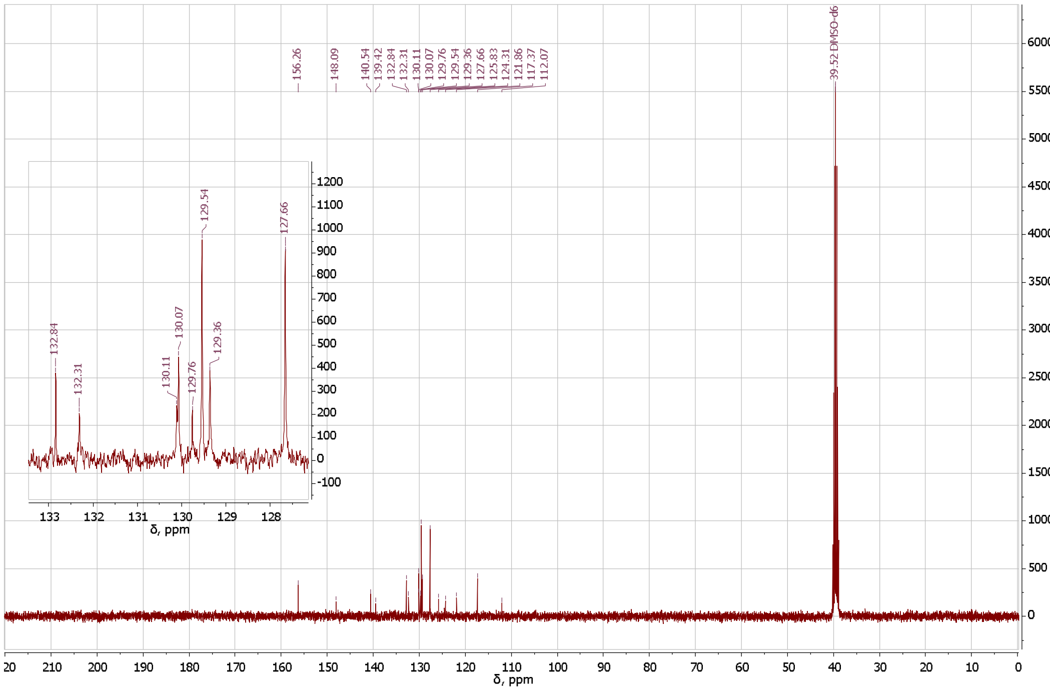


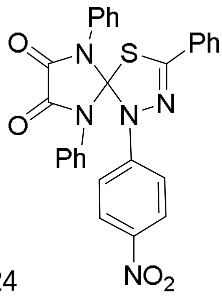


### 1-(4-Nitrophenyl)-3,6,9-triphenyl-4-thia-1,2,6,9-tetraazaspiro[4.4]non-2-ene-7,8-dione (4b)

[1]H NMR (400 MHz, $CDCl_3$) δ 8.33 – 8.27 (m, 2H, Ar), 7.55 – 7.51 (m, 2H, Ar), 7.46 – 7.42 (m, 2H, Ar), 7.42 – 7.33 (m, 9H, Ar), 7.25 – 7.20 (m, 4H, Ar).

$^{13}C$ NMR (101 MHz, $CDCl_3$) δ 155.0, 151.7, 148.8, 145.2, 144.4, 131.4, 130.0, 130.0, 129.2, 126.6, 124.9, 124.7, 120.8, 120.8.

HRMS (ESI): calcd for $C_{28}H_{19}N_5O_4S$ $(M+H)^+$ 522.1231, found 522.1226.

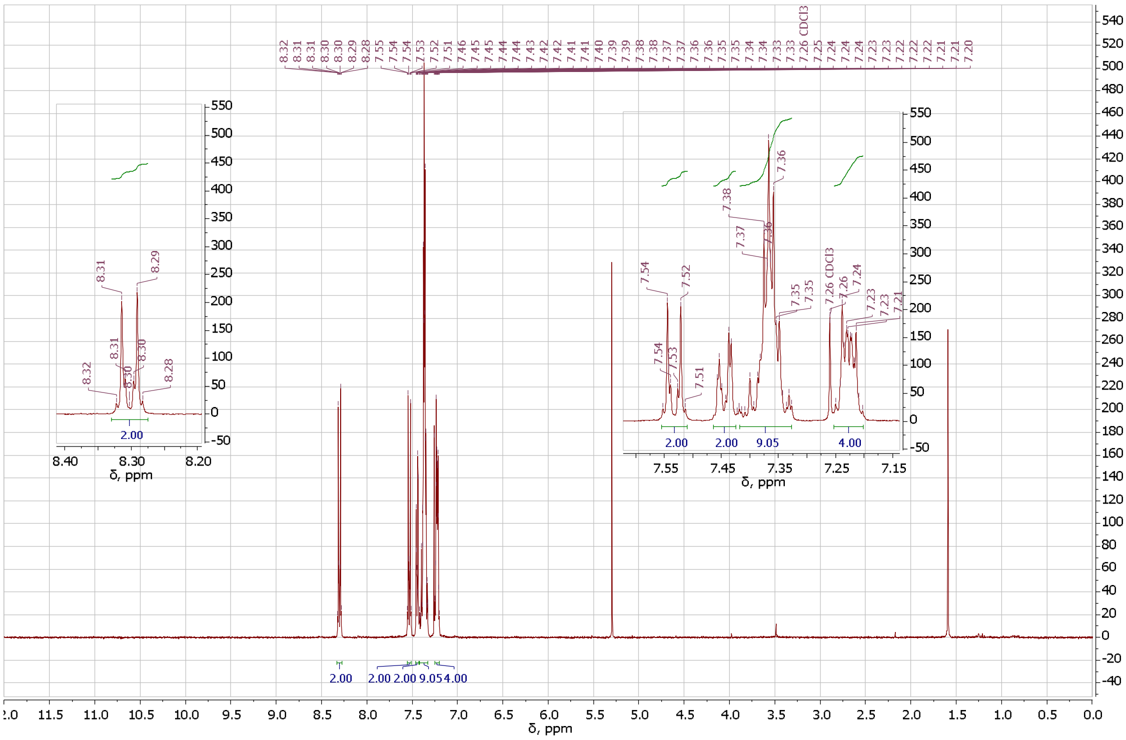


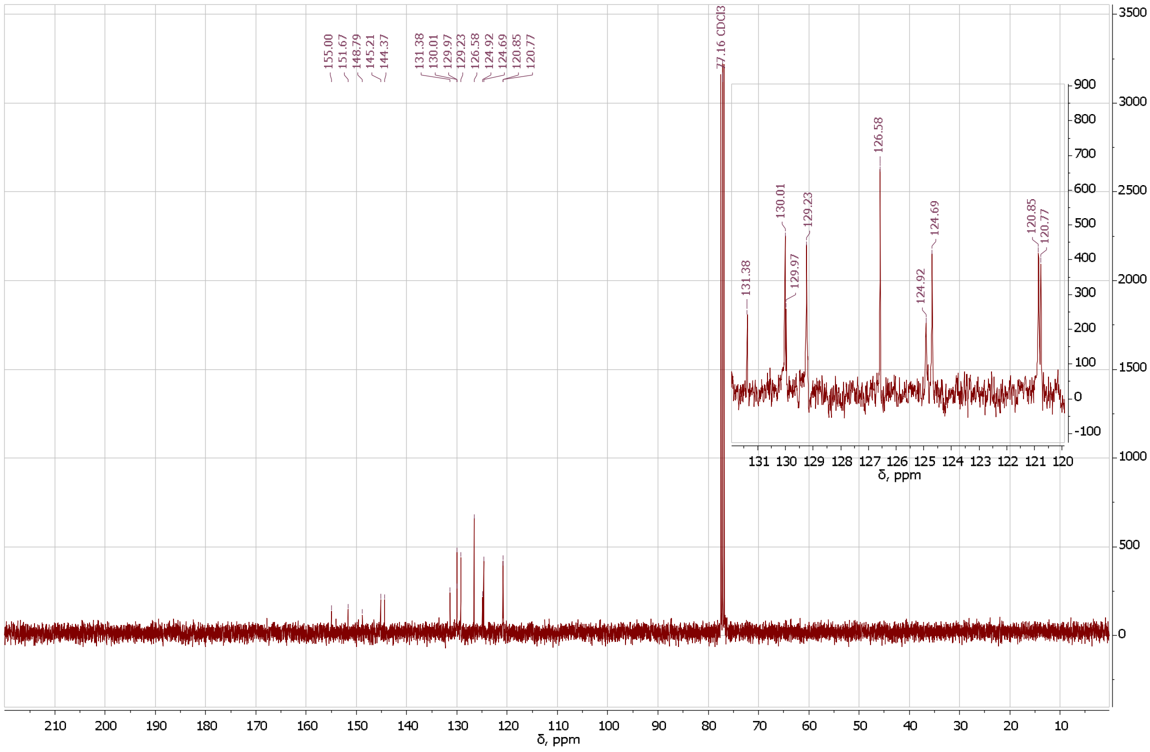


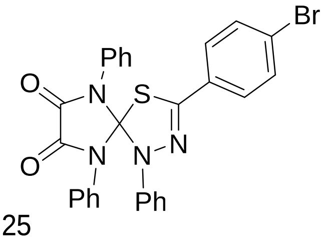


**3-(4-Bromophenyl)-1,6,9-triphenyl-4-thia-1,2,6,9-tetraazaspiro[4.4]non-2-ene-7,8-dione (4c)**

$^{1}$H NMR (400 MHz, $CDCl_3$) δ 7.46 – 7.42 (m, 2H, Ar), 7.42 – 7.37 (m, 2H, Ar), 7.36 – 7.31 (m, 8H, Ar), 7.30 – 7.27 (m, 2H, Ar), 7.26 – 7.19 (m, 5H, Ar).

$^{13}$C NMR (101 MHz, $CDCl_3$) δ 155.78, 140.64, 140.00, 132.55, 132.00, 129.99, 129.65, 129.51, 128.98, 127.55, 127.52, 124.79, 124.43, 118.21, 112.00.

HRMS (ESI): calcd for $C_{28}H_{19}BrN_4O_2S$ $(M+H)^+$ 555.0485, found 555.0487.

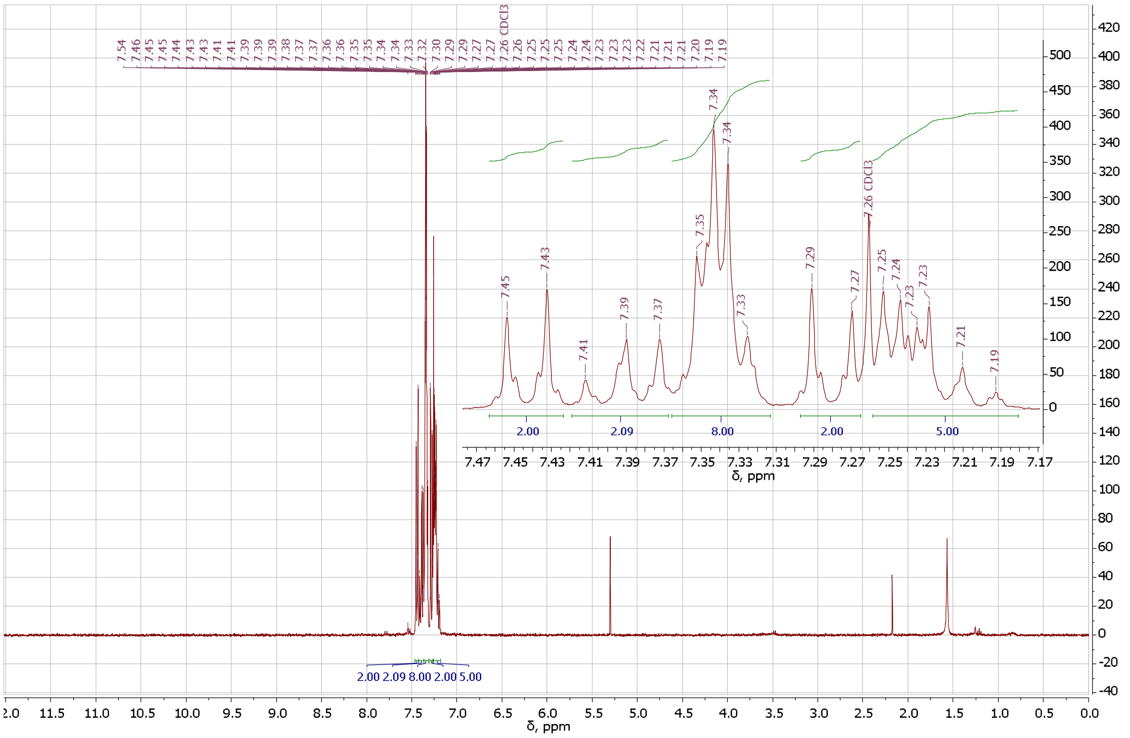


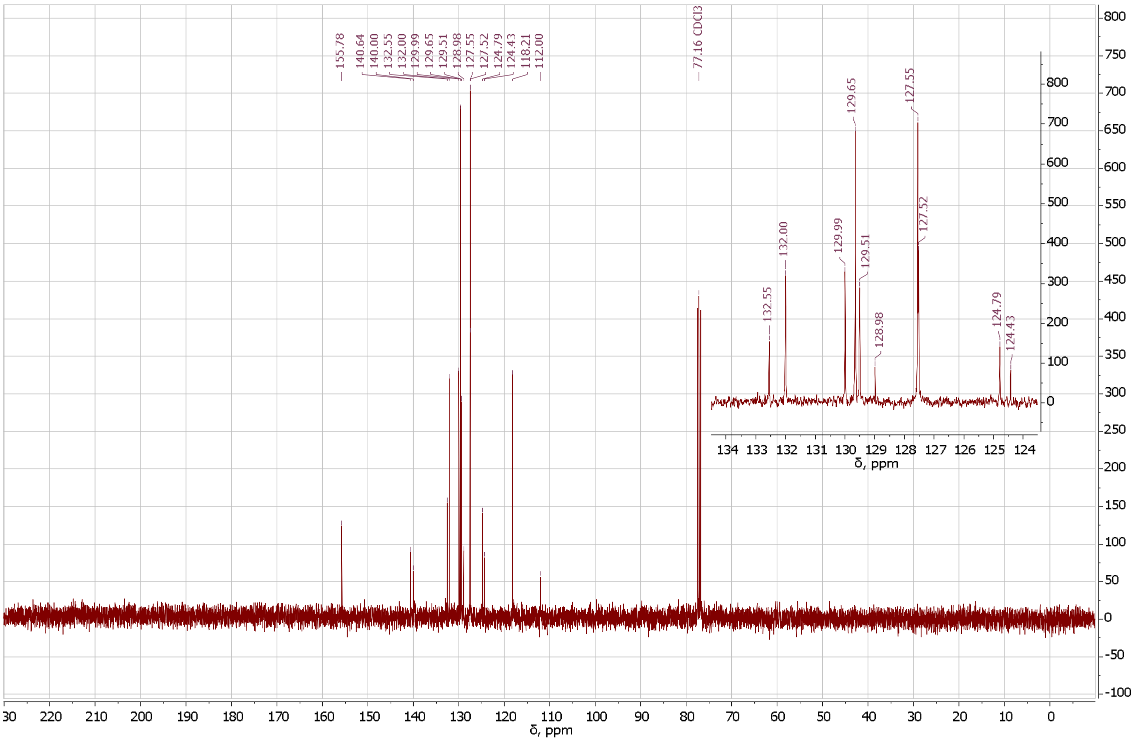


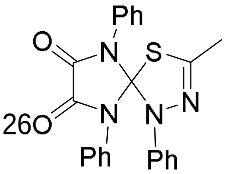


**3-Methyl-1,6,9-triphenyl-4-thia-1,2,6,9-tetraazaspiro[4.4]non-2-ene-7,8-dione (4d)**

[1]H NMR (400 MHz, $CDCl_3$) δ 7.39 – 7.32 (m, 8H, Ar), 7.26 – 7.20 (m, 6H, Ar), 7.14 (t, *J* = 7.3 Hz, 1H, Ar), 2.00 (s, 3H, $CH_3$).

[13]C NMR (101 MHz, $CDCl_3$) δ 155.8, 140.9, 139.2, 132.8, 129.9, 129.6, 129.4, 127.6, 124.1, 117.7, 112.0, 16.5.

HRMS (ESI): calcd for $C_{23}H_{18}N_4O_2S$ $(2M+Na)^+$ 851.2193, found 851.2186.

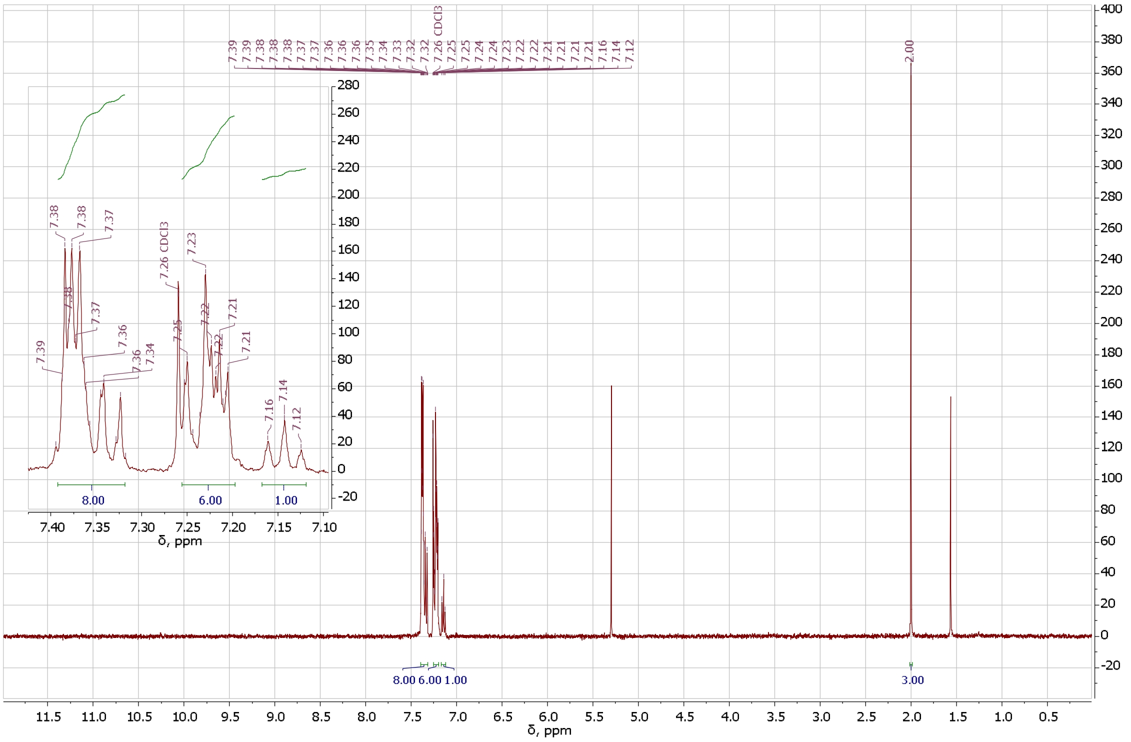

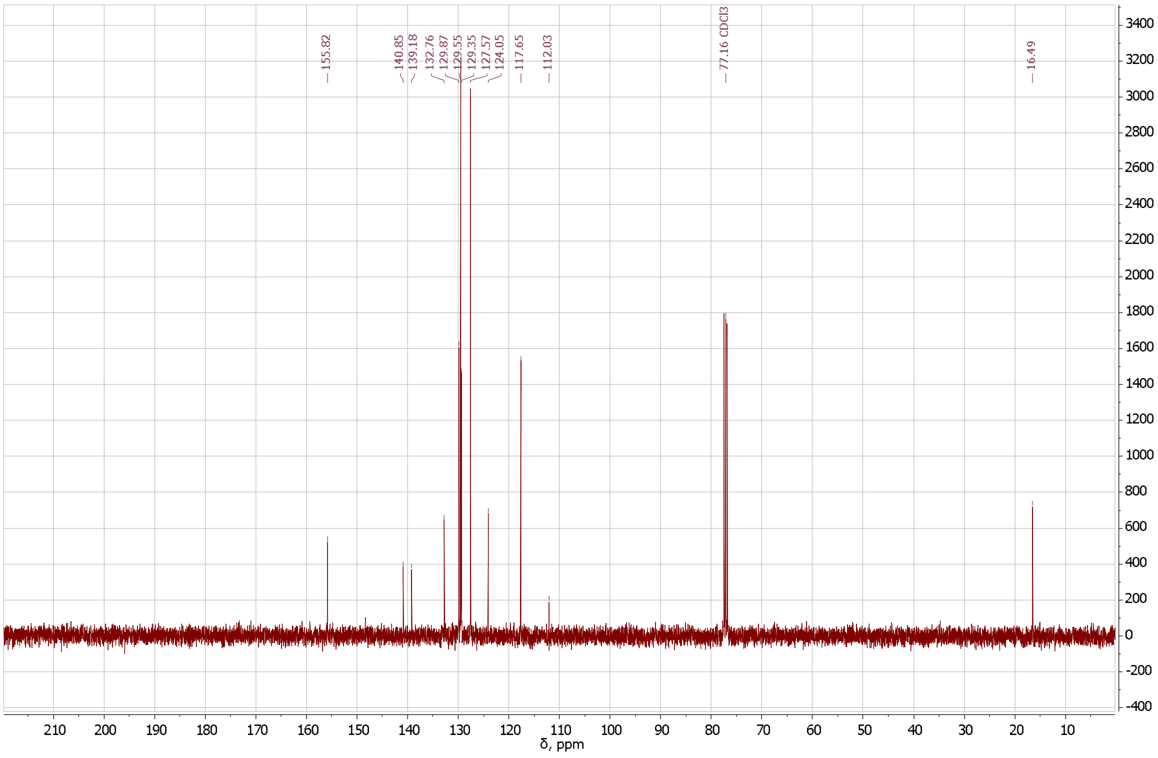

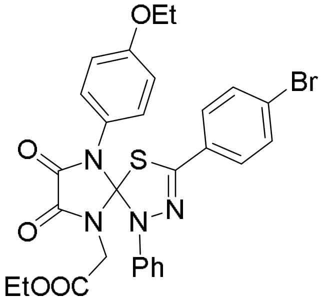


**Ethyl 2-(3-(4-bromophenyl)-9-(4-ethoxyphenyl)-7,8-dioxo-1-phenyl-4-thia-1,2,6,9-tetraazaspiro[4.4]non-2-en-6-yl)acetate (4e)**

$^1$H NMR (400 MHz, $CDCl_3$) δ 7.48 (d, *J* = 8.5 Hz, 2H, Ar), 7.39 – 7.32 (m, 4H, Ar), 7.24 (d, *J* = 8.4 Hz, 2H, Ar), 7.18 (t, *J* = 7.3 Hz, 1H, Ar), 7.08 (d, *J* = 9.0 Hz, 2H, Ar), 6.79 (d, *J* = 9.0 Hz, 2H, Ar), 4.31 (d, *J* = 17.6 Hz, 1H, $NC\underline{H_2}$), 4.28 – 4.13 (m, 2H, $COOC\underline{H_2}CH_3$), 4.01 – 3.90 (m, 3H, $NC\underline{H_2}$+$OC\underline{H_2}CH_3$), 1.34 (t, *J* = 7.0 Hz, 3H, $OCH_2C\underline{H_3}$), 1.24 (t, *J* = 7.1 Hz, 3H, $COOCH_2C\underline{H_3}$).

$^{13}$C NMR (101 MHz, $CDCl_3$) δ 166.1, 159.6, 156.4, 155.4, 140.4, 140.0, 132.1, 129.9, 129.0, 128.9, 127.6, 125.2, 124.5, 124.2, 118.8, 115.2, 111.0, 63.7, 62.4, 41.9, 14.7, 14.1.

HRMS (ESI): calcd for $C_{28}H_{25}BrN_4O_5S$ $(M+H)^+$ 609.0802, found 609.0803.

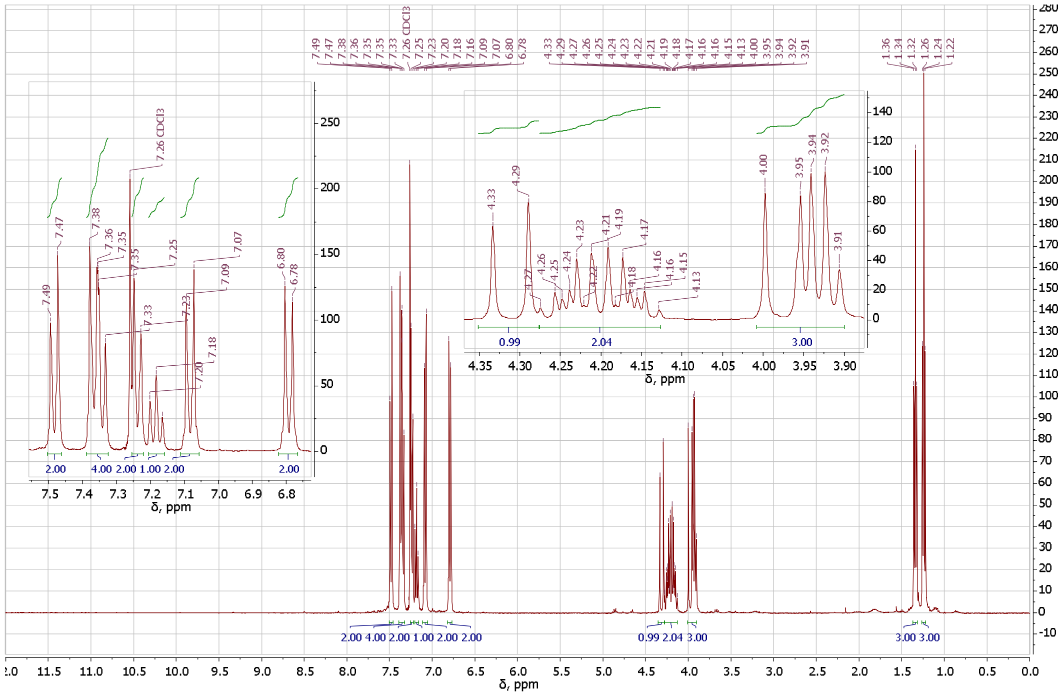

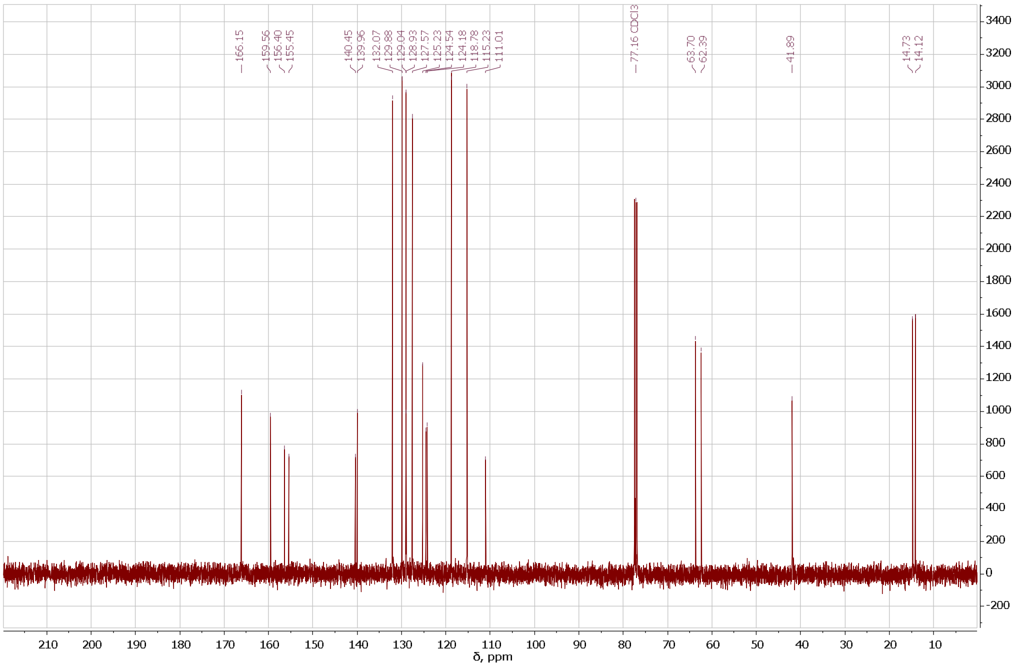


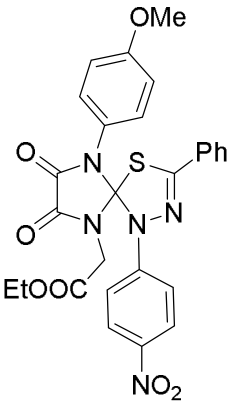


**Ethyl 2-(9-(4-methoxyphenyl)-1-(4-nitrophenyl)-7,8-dioxo-3-phenyl-4-thia-1,2,6,9-tetraazaspiro[4.4]non-2-en-6-yl)acetate (4f)**

$^1$H NMR (400 MHz, $CDCl_3$) δ 8.25 (d, *J* = 9.3 Hz, 2H, Ar), 7.58 – 7.52 (m, 2H, Ar), 7.47 – 7.39 (m, 5H, Ar), 7.03 (d, *J* = 9.0 Hz, 2H, Ar), 6.82 (d, *J* = 9.0 Hz, 2H, Ar), 4.39 (d, *J* = 17.5 Hz, 1H, $NC\underline{H}_2$), 4.20 (qq, *J* = 10.8, 7.1 Hz, 2H, $COOC\underline{H}_2CH_3$), 4.06 (d, *J* = 17.5 Hz, 1H, $NC\underline{H}_2$), 3.74 (s, 3H, $OCH_3$), 1.24 (t, *J* = 7.1 Hz, 3H, $COOCH_2C\underline{H}_3$).

$^{13}$C NMR (101 MHz, $CDCl_3$) δ 165.8, 160.5, 145.7, 131.4, 129.4, 129.2, 129.0, 126.7, 125.7, 123.8, 117.0, 115.1, 110.6, 62.6, 55.6, 42.1, 14.2.

HRMS (ESI): calcd for $C_{27}H_{23}N_5O_7S$ $(M+Na)^+$ 584.1210, found 584.1217.

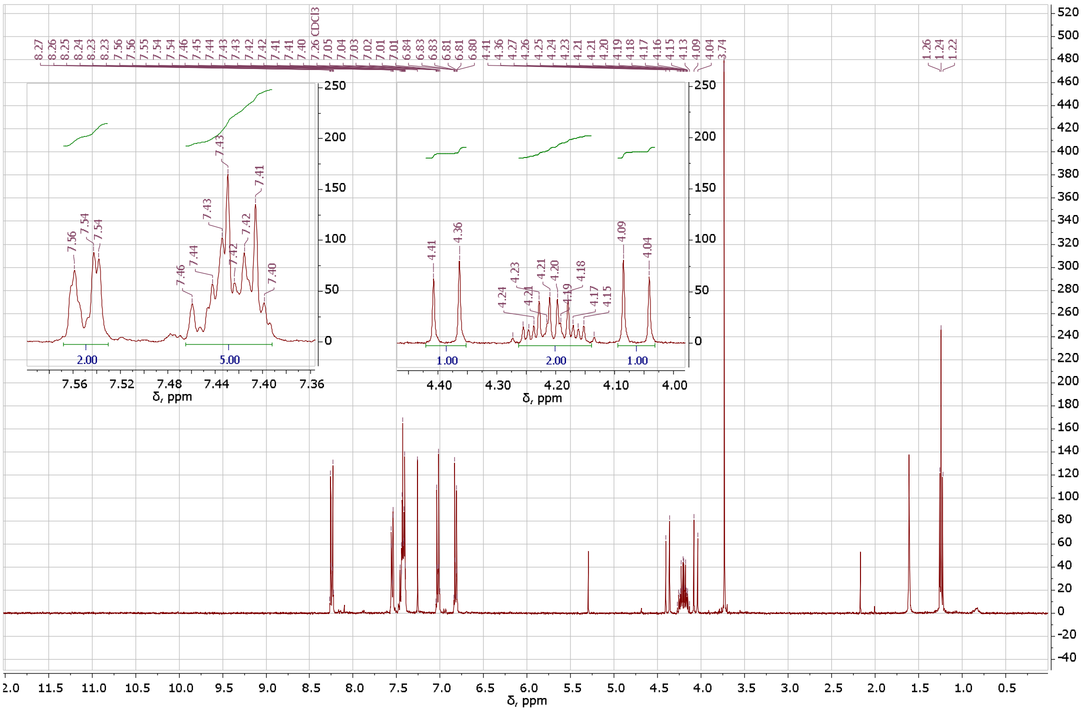

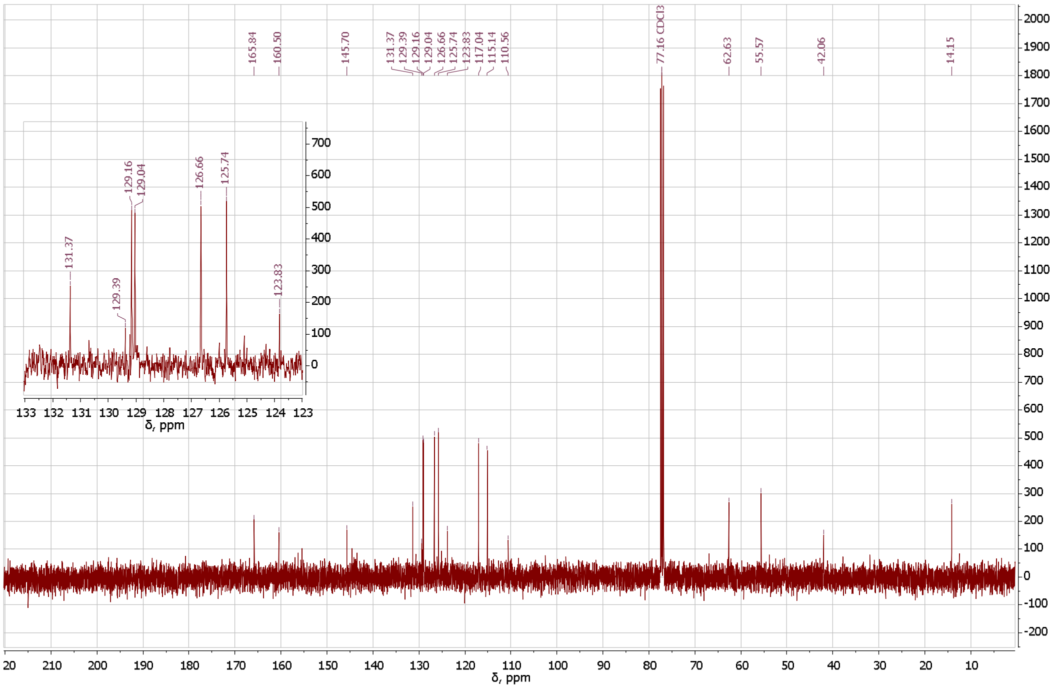


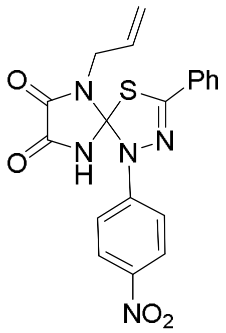


## 6-Allyl-1-(4-nitrophenyl)-3-phenyl-4-thia-1,2,6,9-tetraazaspiro[4.4]non-2-ene-7,8-dione (4g)

$^{1}$H NMR (400 MHz, $CDCl_3$) δ 8.53 – 8.39 (m, 4H, Ar), 7.96 – 7.91 (m, 2H, Ar), 7.62 – 7.53 (m, 3H, Ar), 7.51 – 7.44 (m, 1H, NH), 5.90 (ddt, *J* = 17.2, 10.2, 5.6 Hz, 1H, CH), 5.29 – 5.18 (m, 2H, $=CH_2$), 4.06 – 4.00 (m, 2H, $NCH_2$).

$^{13}$C NMR (101 MHz, $CDCl_3$) δ 168.9, 167.4, 160.2, 158.2, 146.7, 143.6, 133.4, 133.4, 132.4, 129.6, 128.8, 127.1, 124.9, 124.7, 117.1, 42.2.

HRMS (ESI): calcd for $C_{19}H_{15}N_5O_4S$ $(2M+Na)^+$ 841.1582, found 841.1582.

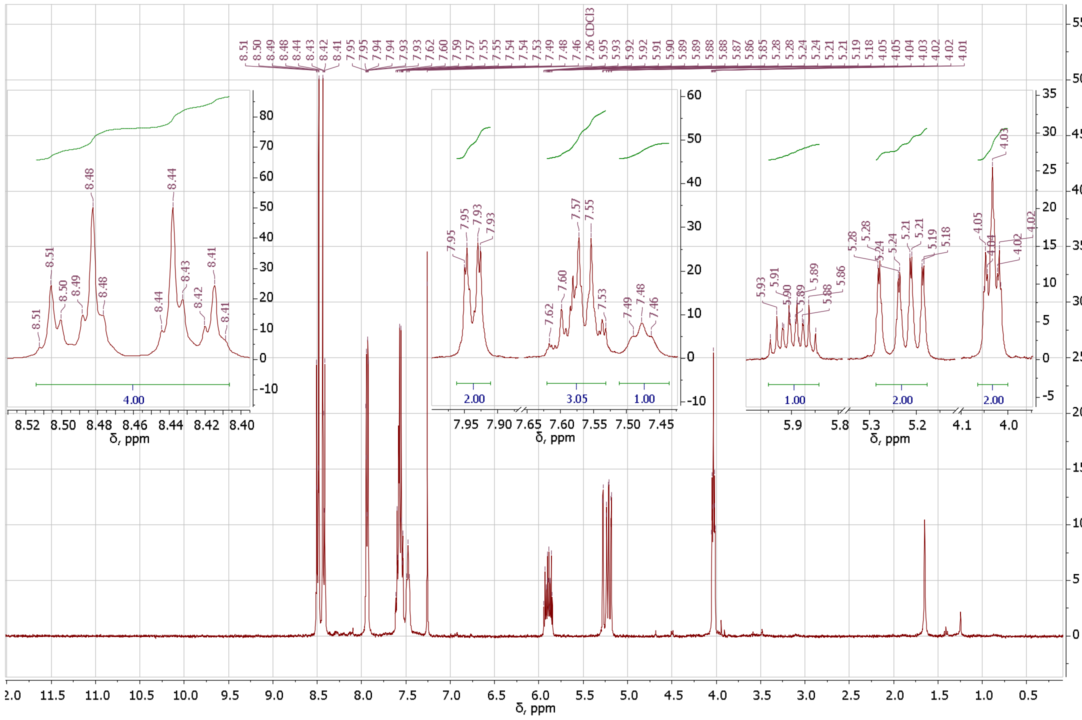

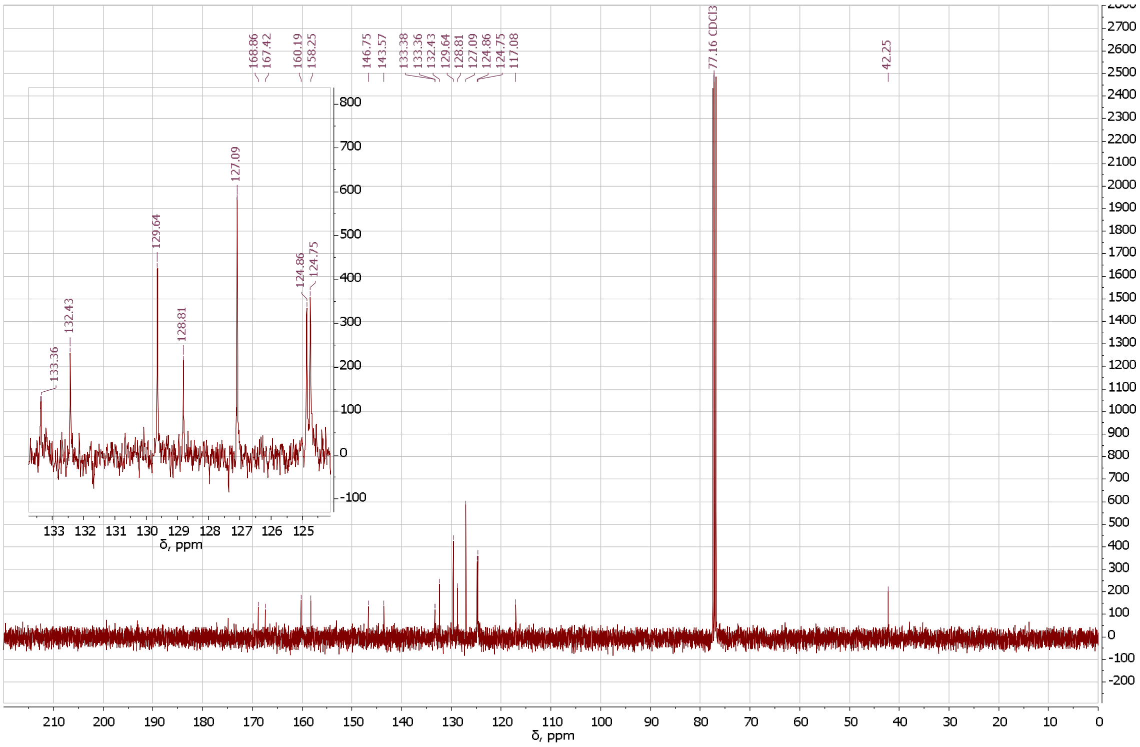


**$^1$H, $^{13}$C NMR and HRMS spectral data of 1,2,4,6,8-pentaazaspiro[4.4]non-2-ene-7,9-diones 5a-n**

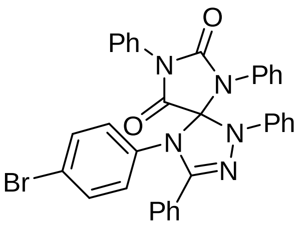


**4-(4-Bromophenyl)-1,3,6,8-tetraphenyl-1,2,4,6,8-pentaazaspiro[4.4]non-2-ene-7,9-dione (5a)**

$^1$H NMR (400 MHz, $CDCl_3$) δ 7.53 – 7.50 (m, 2H, Ar), 7.47 – 7.42 (m, 3H, Ar), 7.42 – 7.38 (m, 3H, Ar), 7.38 – 7.35 (m, 4H, Ar), 7.35 – 7.26 (m, 7H, Ar), 7.15 – 7.11 (m, 2H, Ar), 7.07 – 7.02 (m, 1H, Ar), 6.98 – 6.93 (m, 2H, Ar).

$^{13}$C NMR (101 MHz, $CDCl_3$) δ 165.0, 151.8, 146.6, 141.4, 135.7, 133.9, 132.9, 130.6, 130.0, 129.7, 129.5, 129.5, 129.0, 128.7, 128.6, 127.9, 127.6, 126.3, 126.1, 124.9, 122.7, 121.9, 115.6, 97.5.

HRMS (ESI): calcd for $C_{34}H_{24}BrN_5O_2$ $(M+H)^+$ 614.1186, found 614.1193.

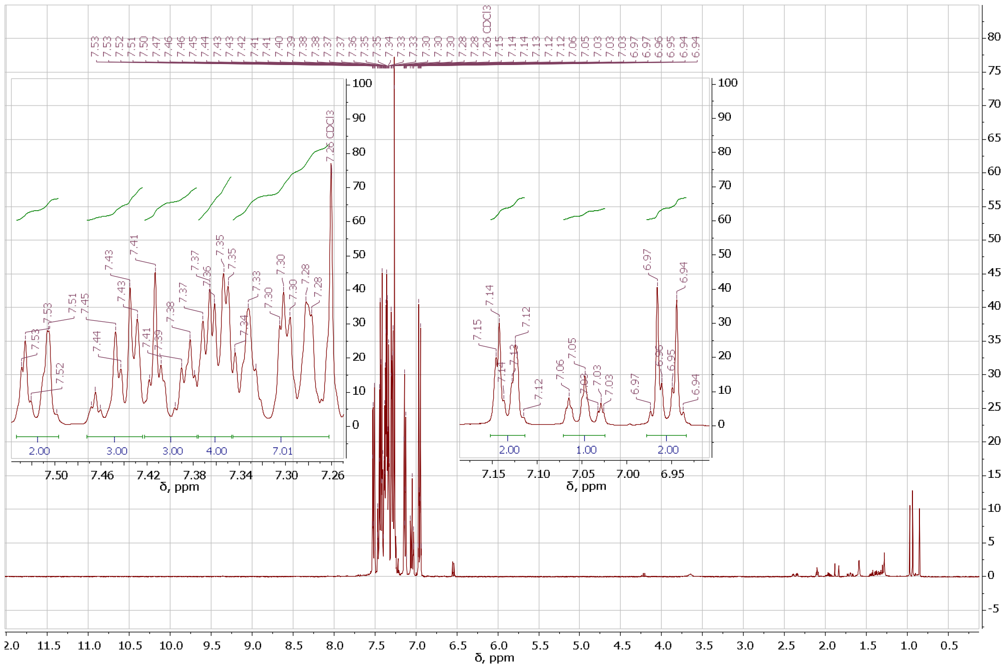


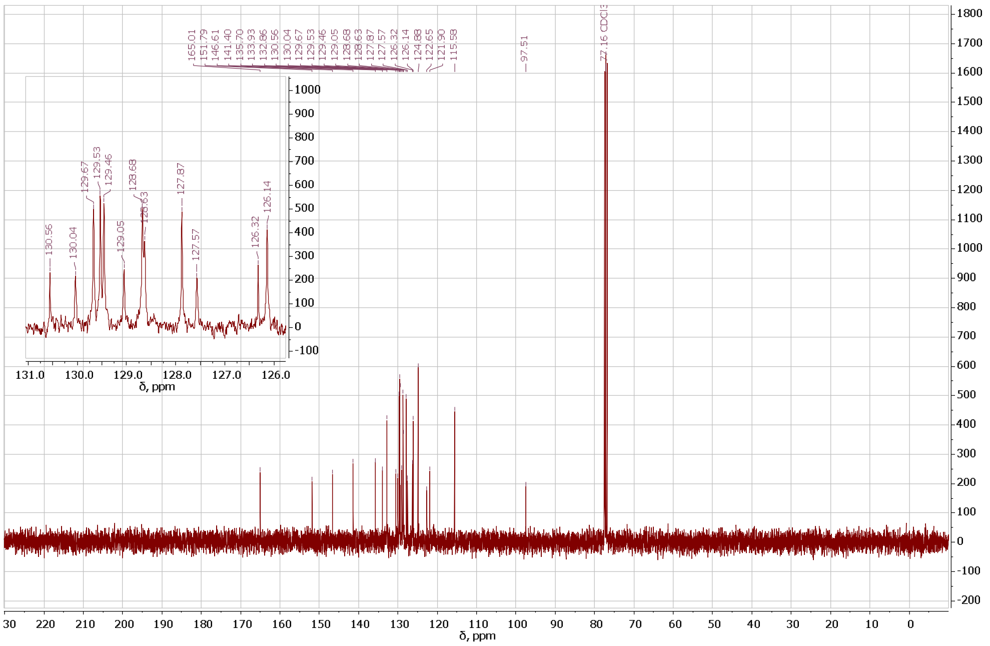


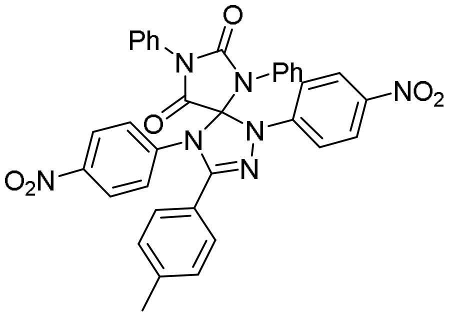


## 1,4-Bis(4-nitrophenyl)-6,8-diphenyl-3-(p-tolyl)-1,2,4,6,8-pentaazaspiro[4.4]non-2-ene-7,9-dione (5b)

$^{1}$H NMR (400 MHz, $CDCl_3$) δ 8.28 – 8.23 (m, 2H, Ar), 8.20 – 8.16 (m, 2H, Ar), 7.52 – 7.44 (m, 3H, Ar), 7.39 – 7.34 (m, 4H, Ar), 7.33 – 7.27 (m, 3H, Ar), 7.25 – 7.18 (m, 6H, Ar), 7.13 (d, *J* = 8.1 Hz, 2H, Ar), 2.35 (s, 3H, $CH_3$).

$^{13}$C NMR (101 MHz, $CDCl_3$) δ 164.0, 151.3, 147.8, 146.5, 145.5, 142.1, 141.9, 141.8, 132.8, 130.1, 130.0, 129.9, 129.8, 129.6, 128.6, 128.0, 126.6, 126.1, 125.7, 125.2, 125.1, 122.2, 113.6, 95.5, 21.6.

HRMS (ESI): calcd for $C_{35}H_{25}N_7O_6$ $(M+Na)^+$ 662.1759, found 662.1765.

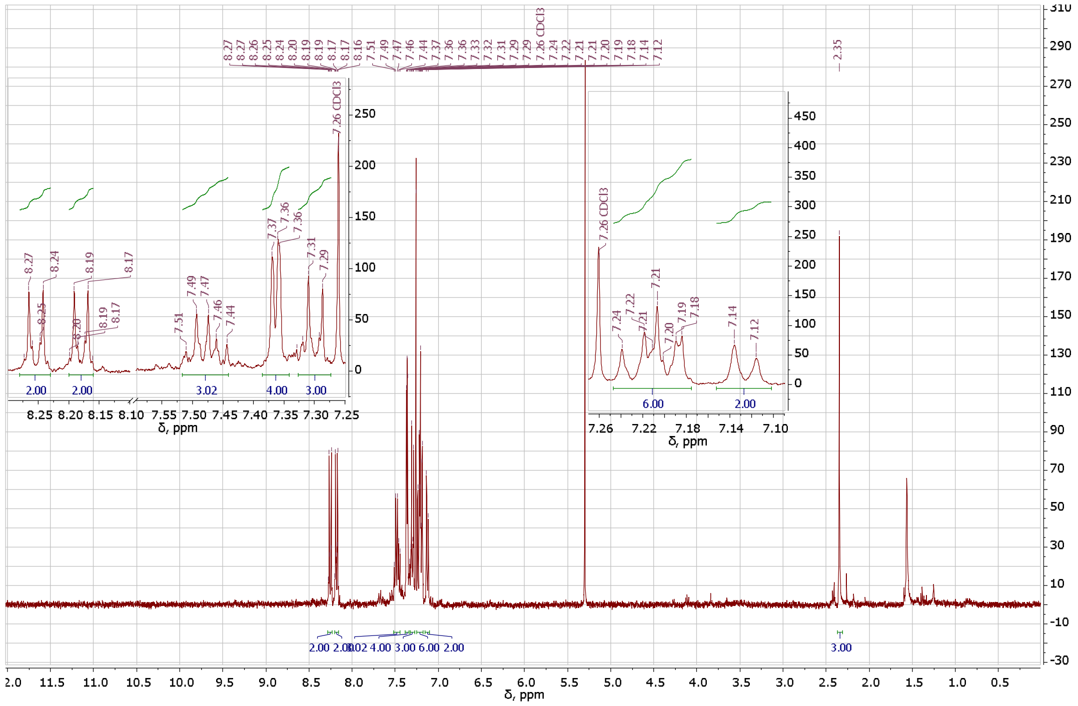


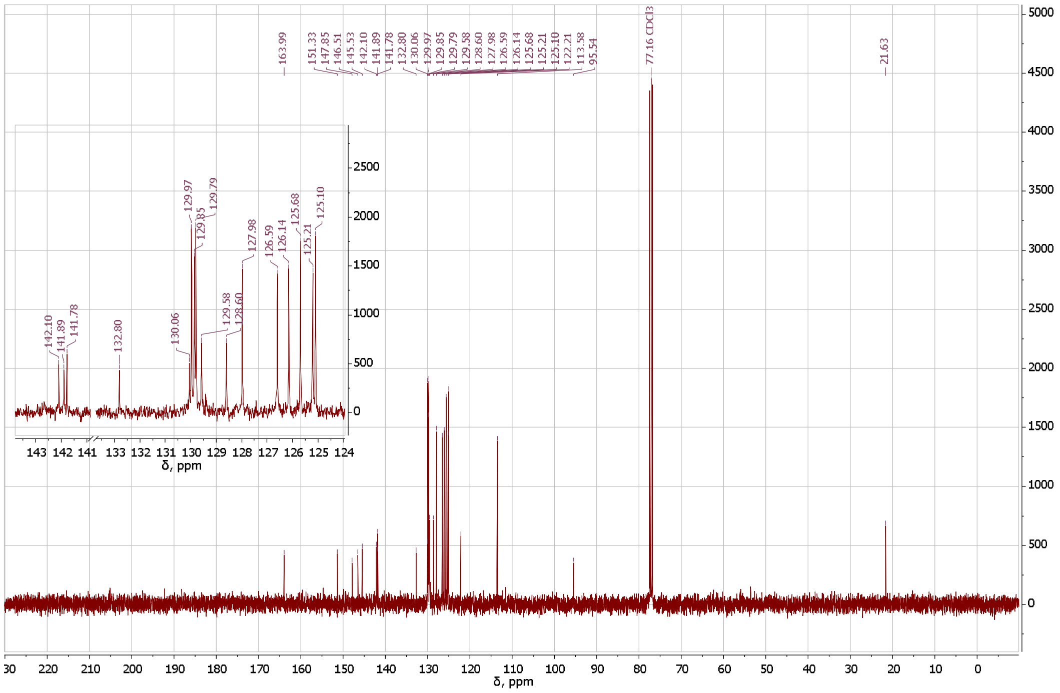


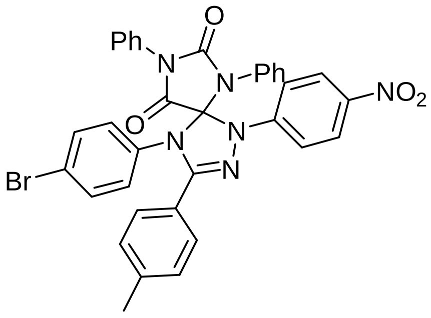


## 4-(4-Bromophenyl)-1-(4-nitrophenyl)-6,8-diphenyl-3-(p-tolyl)-1,2,4,6,8-pentaazaspiro[4.4]non-2-ene-7,9-dione (5c)

$^{1}$H NMR (400 MHz, DMSO-d6) δ 8.34 (d, *J* = 9.4 Hz, 2H, Ar), 7.69 (d, *J* = 8.7 Hz, 2H, Ar), 7.55 – 7.50 (m, 3H, Ar), 7.49 – 7.43 (m, 4H, Ar), 7.39 – 7.35 (m, 3H, Ar), 7.33 (d, *J* = 8.2 Hz, 2H, Ar), 7.23 – 7.17 (m, 4H, Ar), 7.15 (d, *J* = 8.6 Hz, 2H, Ar), 2.29 (s, 3H, $CH_3$).

$^{13}$C NMR (101 MHz, DMSO-d6) δ 163.5, 148.2, 145.2, 140.7, 135.0, 133.1, 132.5, 129.9, 129.9, 129.8, 129.6, 129.5, 128.9, 128.8, 128.6, 127.9, 126.8, 126.5, 126.1, 122.1, 121.5, 113.1, 95.3, 21.0.

HRMS (ESI): calcd for $C_{35}H_{25}BrN_6O_4$ $(M+Na)^+$ 695.1013, found 695.1015.

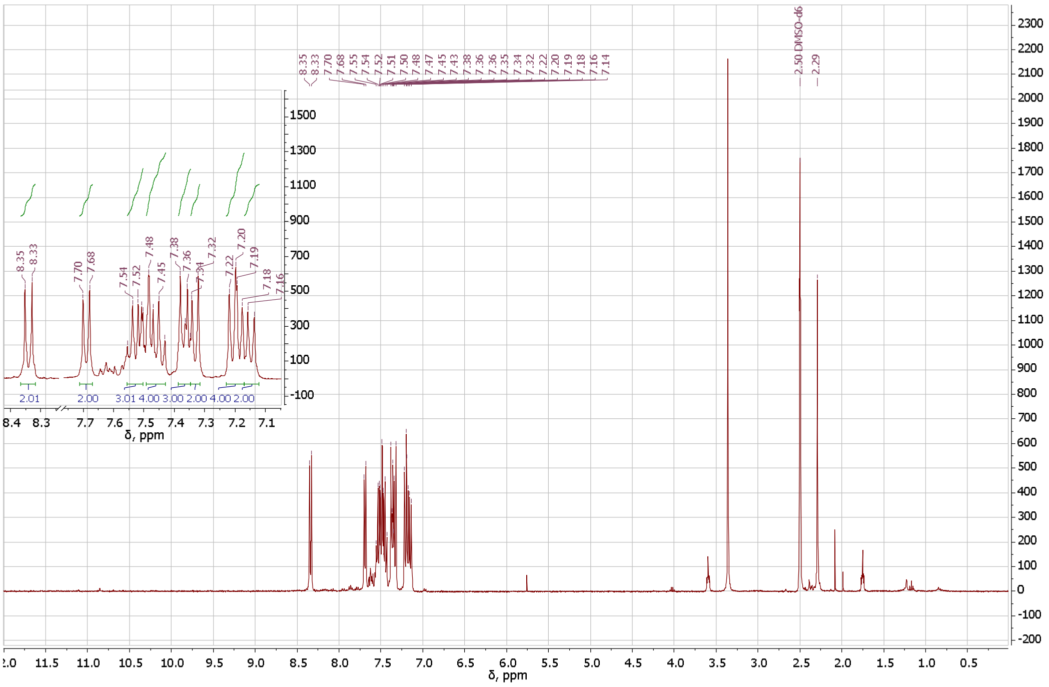

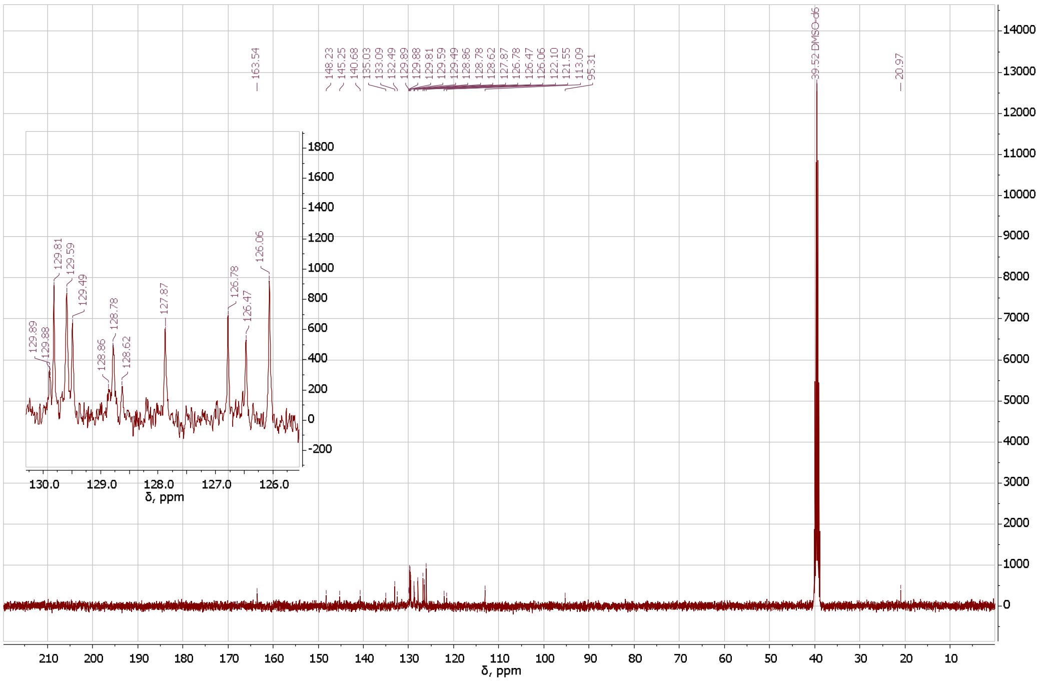

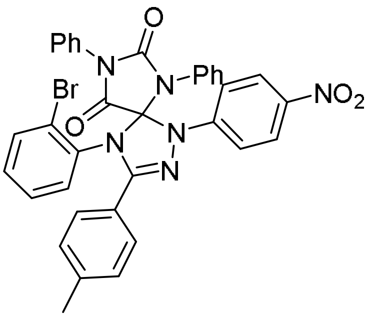


### 4-(2-Bromophenyl)-1-(4-nitrophenyl)-6,8-diphenyl-3-(p-tolyl)-1,2,4,6,8-pentaazaspiro[4.4]non-2-ene-7,9-dione (5d)

Isomer I:

$^1$H NMR (400 MHz, $CDCl_3$) δ 8.20 – 8.15 (m, 2H, Ar), 7.68 – 7.65 (m, 1H, Ar), 7.53 – 7.49 (m, 2H, Ar), 7.47 – 7.41 (m, 5H, Ar), 7.29 – 7.24 (m, 2H, Ar), 7.23 – 7.17 (m, 4H, Ar), 7.17 – 7.11 (m, 6H, Ar), 2.34 (s, 3H, $CH_3$).

$^{13}$C NMR (101 MHz, $CDCl_3$) δ 163.6, 151.6, 150.0, 145.8, 141.4, 141.3, 137.1, 134.2, 133.5, 133.2, 130.6, 130.5, 129.7, 129.6, 129.4, 129.2, 128.7, 127.8, 127.7, 126.0, 125.9, 125.1, 123.3, 122.9, 113.2, 96.2, 21.6.

HRMS (ESI): calcd for $C_{35}H_{25}BrN_6O_4$ $(M+Na)^+$ 695.1013, found 695.1016.

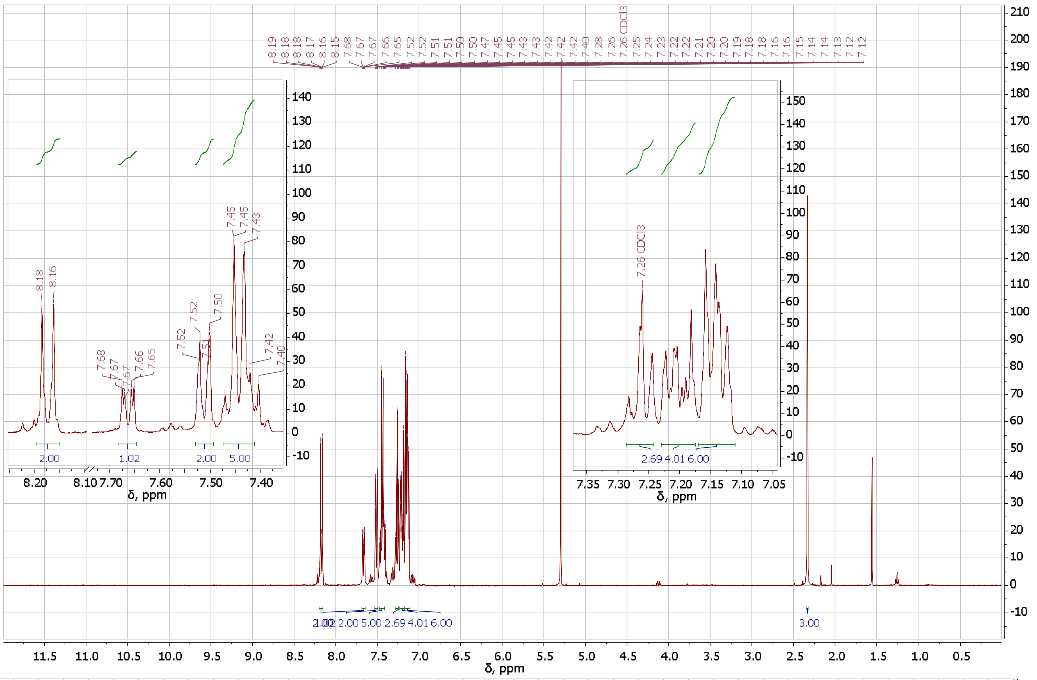


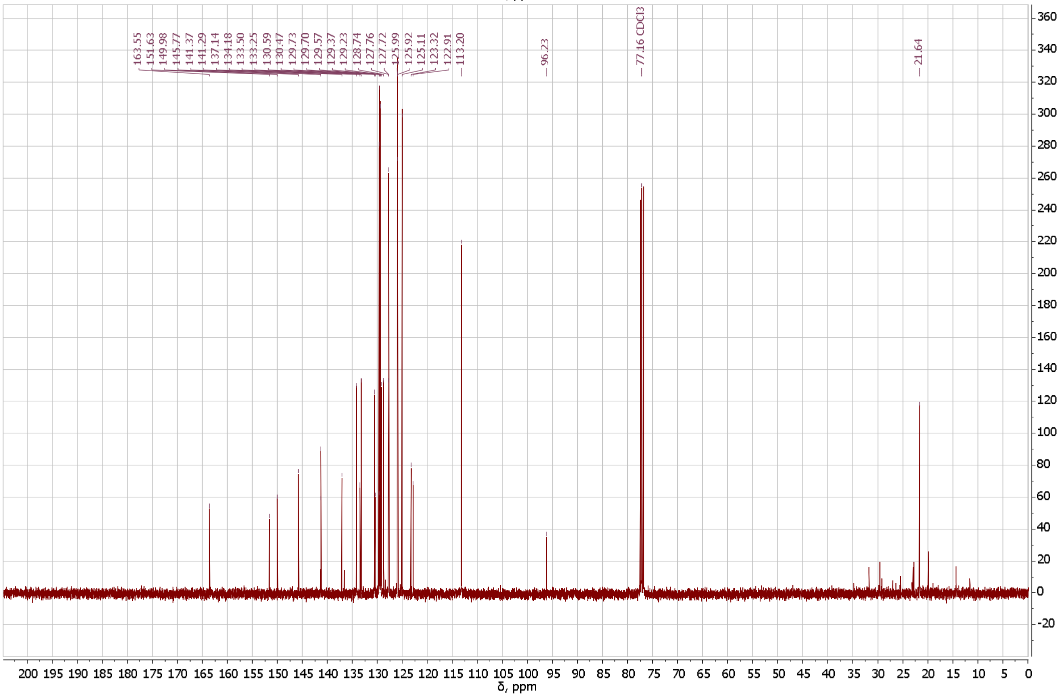


Isomer II:

$^1$H NMR (400 MHz, $CDCl_3$) δ 8.24 – 8.19 (m, 2H, Ar), 7.60 – 7.55 (m, 2H, Ar), 7.45 – 7.39 (m, 6H, Ar), 7.34 – 7.27 (m, 5H, Ar), 7.26 – 7.21 (m, 3H, Ar), 7.10 – 7.04 (m, 4H, Ar), 2.33 (s, 3H, $CH_3$).

13C NMR (101 MHz, CDCl3) δ 163.5, 151.5, 149.0, 145.6, 141.3, 141.1, 135.6, 134.9, 133.4, 130.8, 130.4, 129.8, 129.6, 129.5, 129.3, 128.9, 128.8, 128.0, 127.3, 126.1, 126.0, 125.1, 124.6, 124.1, 113.3, 95.5, 21.7.

HRMS (ESI): calcd for $C_{35}H_{25}BrN_6O_4$ $(M+Na)^+$ 695.1013, found 695.1022.

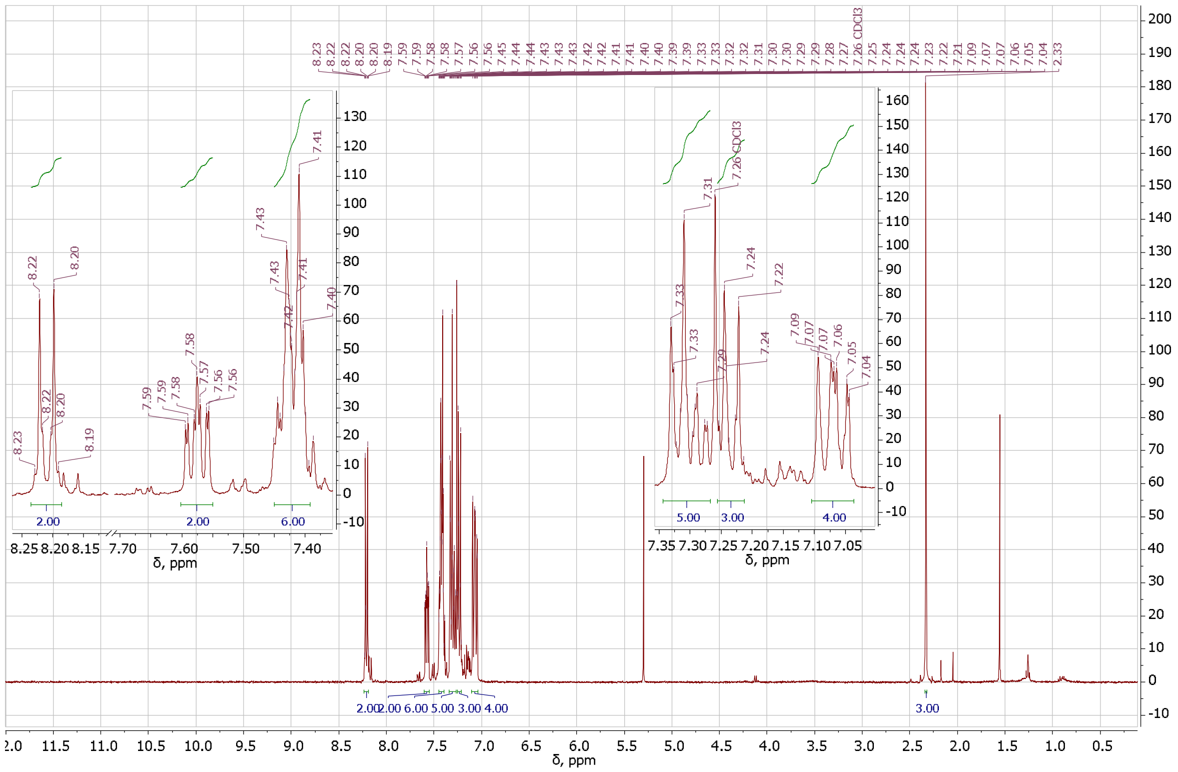

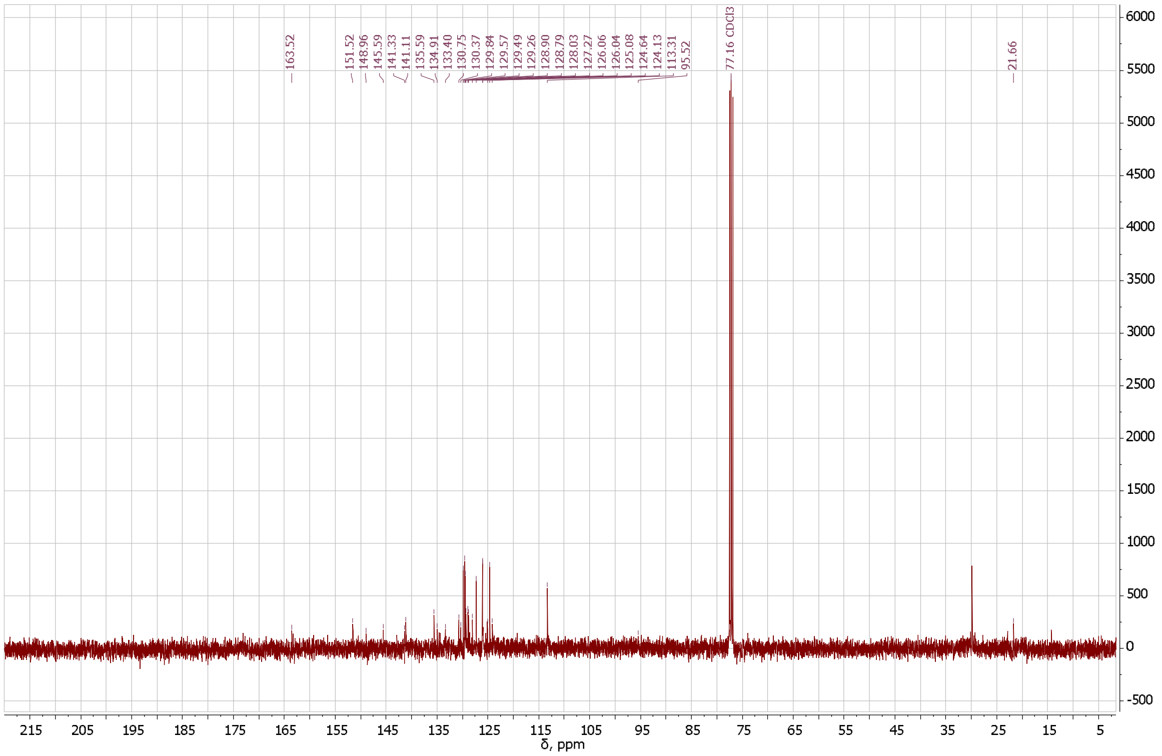

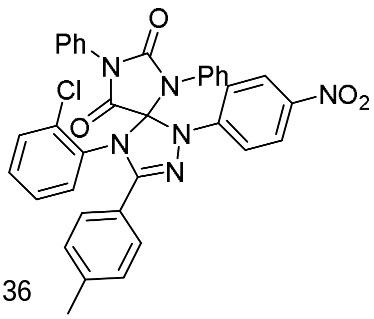


## 4-(2-Chlorophenyl)-1-(4-nitrophenyl)-6,8-diphenyl-3-(p-tolyl)-1,2,4,6,8-pentaazaspiro[4.4]non-2-ene-7,9-dione (5e)

$^1$H NMR (400 MHz, DMSO-d6) δ 8.33 (d, $J$ = 9.3 Hz, 2$H^{II}$, Ar), 8.28 (d, $J$ = 9.2 Hz, 2$H^{I}$, Ar), 7.88 (d, $J$ = 7.8 Hz, 1$H^{II}$, Ar), 7.71 – 7.64 (m, 1$H^{I}$+1$H^{II}$, Ar), 7.56 – 7.45 (m, 5$H^{I}$+7$H^{II}$, Ar), 7.44-7.35 (m, 3$H^{I}$+3$H^{II}$, Ar), 7.35-7.30 (m, 5$H^{I}$+4$H^{II}$, Ar), 7.29-7.26 (m, 1$H^{I}$, Ar), 7.23 (d, $J$ =

8.0 Hz, 2H$^{I}$, Ar), 7.19-7.12 (m, 3H$^{I}$+2H$^{II}$, Ar), 7.06 – 7.02 (m, 2H$^{II}$, Ar), 2.29 (s, 3H$^{I}$+3H$^{II}$, $CH_3$).

$^{13}$C NMR (101 MHz, DMSO-d6) δ 163.1, 163.0, 150.9, 150.7, 149.4, 148.7, 145.2, 145.0, 141.2, 141.0, 140.6, 140.5, 134.4, 133.1, 132.9, 132.6, 131.9, 131.7, 131.7, 131.3, 131.1, 130.9, 130.0, 129.8, 129.5, 129.5, 129.4, 129.3, 129.3, 128.8, 128.7, 128.6, 127.9, 127.3, 126.9, 126.8, 126.7, 126.5, 126.3, 126.0, 125.1, 123.4, 122.7, 113.1, 113.1, 113.0, 95.8, 95.1, 40.2, 40.0, 39.7, 39.5, 39.3, 39.1, 38.9, 21.0, 20.8.

HRMS (ESI): calcd for $C_{35}H_{25}ClN_6O_4$ $(M+Na)^+$ 651.1518, found 651.1527.

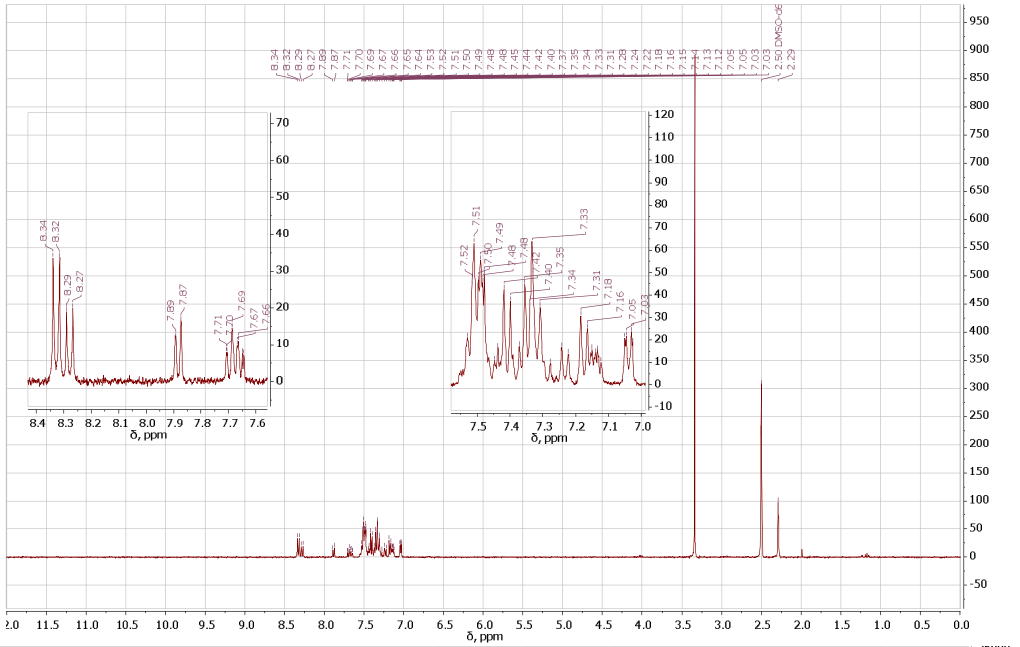


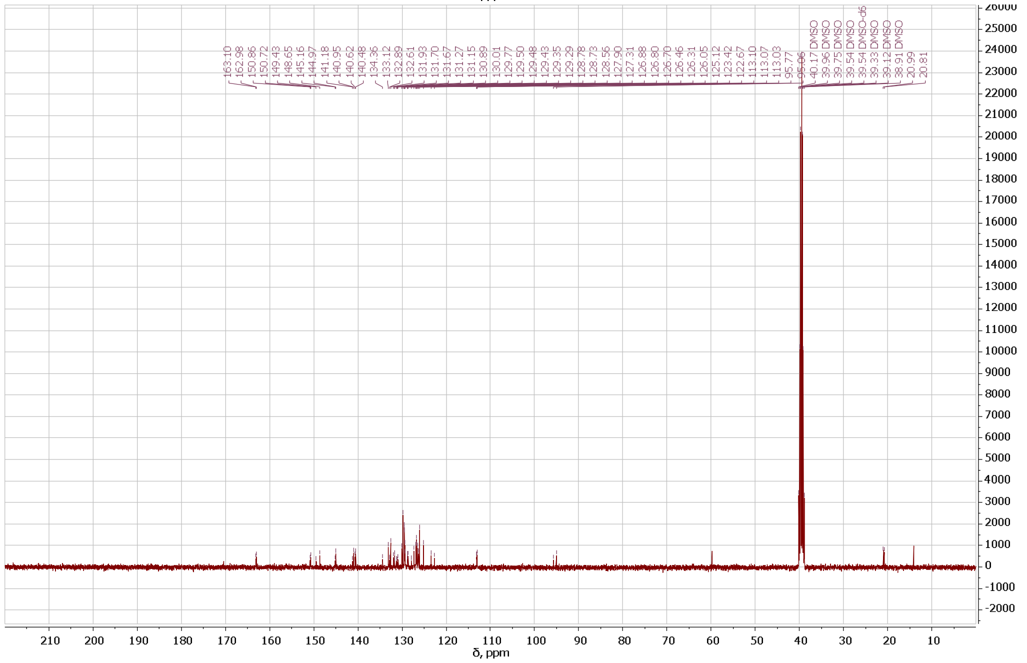


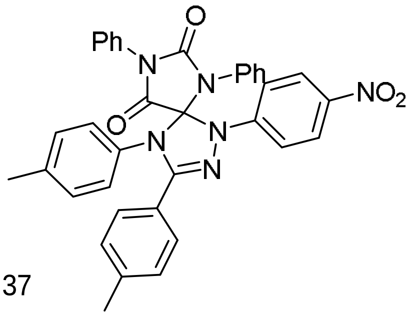

### 1-(4-Nitrophenyl)-6,8-diphenyl-3,4-di-p-tolyl-1,2,4,6,8-pentaazaspiro[4.4]non-2-ene-7,9-dione (5f)

$^{1}$H NMR (400 MHz, DMSO-d6) δ 8.32 (d, *J* = 9.0 Hz, 2H, Ar), 7.52 (d, *J* = 8.0 Hz, 3H, Ar), 7.46 (dd, *J* = 14.1, 6.4 Hz, 4H, Ar), 7.39 – 7.31 (m, 5H, Ar), 7.28 (d, *J* = 7.9 Hz, 2H, Ar), 7.18 (d, *J* =

7.9 Hz, 2H, Ar), 7.07 (d, *J* = 7.6 Hz, 4H, Ar), 2.32 (s, 3H, $CH_3$), 2.28 (s, 3H, $CH_3$).

$^{13}C$ NMR (101 MHz, DMSO-d6) δ 163.7, 150.8, 148.7, 145.4, 140.8, 140.5, 138.6, 132.8, 132.8, 130.5, 130.0, 129.7, 129.4, 129.4, 128.3, 127.8, 127.1, 126.8, 126.4, 125.6, 122.4, 112.9, 95.6, 20.9, 20.6.

HRMS (ESI): calcd for $C_{36}H_{28}N_6O_4$ $(M+Na)^+$ 631.2064, found 631.2072.

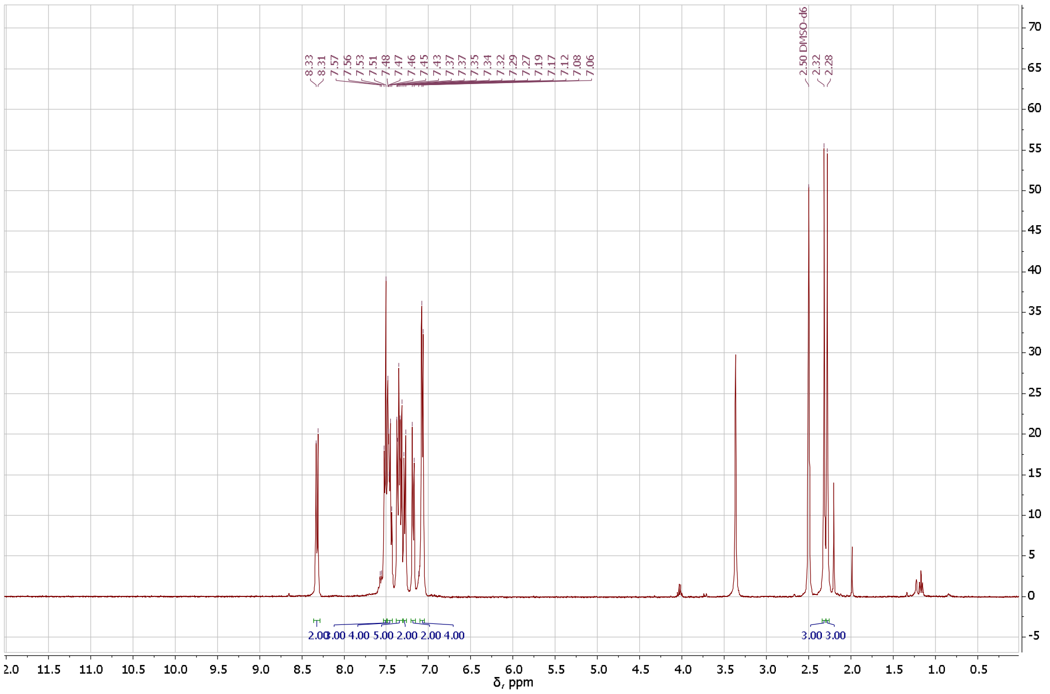


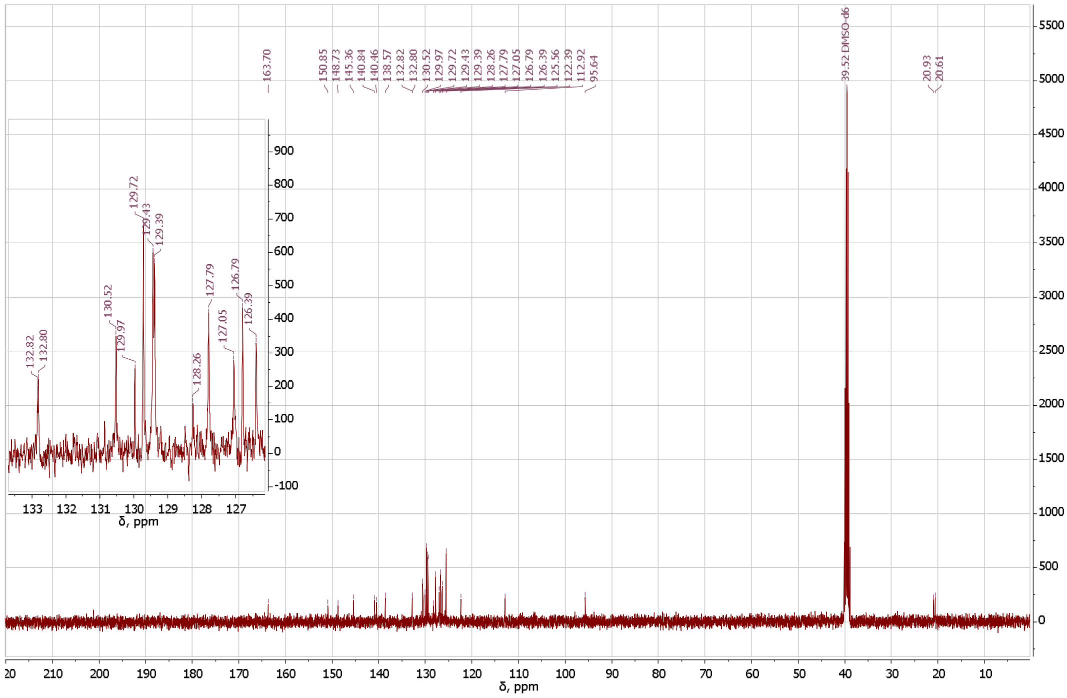


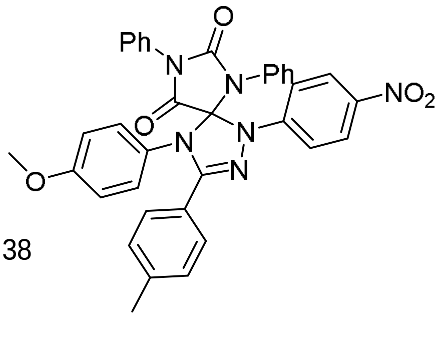


**4-(4-Methoxyphenyl)-1-(4-nitrophenyl)-6,8-diphenyl-3-(p-tolyl)-1,2,4,6,8-pentaazaspiro[4.4]non-2-ene-7,9-dione (5g)**

$^{1}H$ NMR (400 MHz, DMSO-d6) δ 8.31 (d, *J* = 9.5 Hz, 2H, Ar), 7.55 – 7.50 (m, 3H, Ar), 7.50 – 7.43 (m, 4H, Ar), 7.39 – 7.31

(m, 5H, Ar), 7.19 (d, *J* = 7.5 Hz, 2H, Ar), 7.13 (d, *J* = 8.4 Hz, 2H, Ar), 7.10 – 7.06 (m, 2H, Ar), 7.04 (d, *J* = 9.3 Hz, 2H, Ar), 3.76 (s, 3H, $OCH_3$), 2.28 (s, 3H, $CH_3$).

$^{13}C$ NMR (101 MHz, DMSO-d6) δ 163.8, 159.3, 150.9, 148.9, 145.4, 140.9, 140.4, 133.0, 130.0, 129.7, 129.5, 129.4, 129.4, 129.1, 128.2, 127.8, 127.6, 126.8, 126.4, 125.3, 122.3, 115.2, 112.9, 95.8, 55.6, 21.0.

HRMS (ESI): calcd for $C_{36}H_{28}N_6O_5$ $(M+H)^+$ 625.2194, found 625.2199.

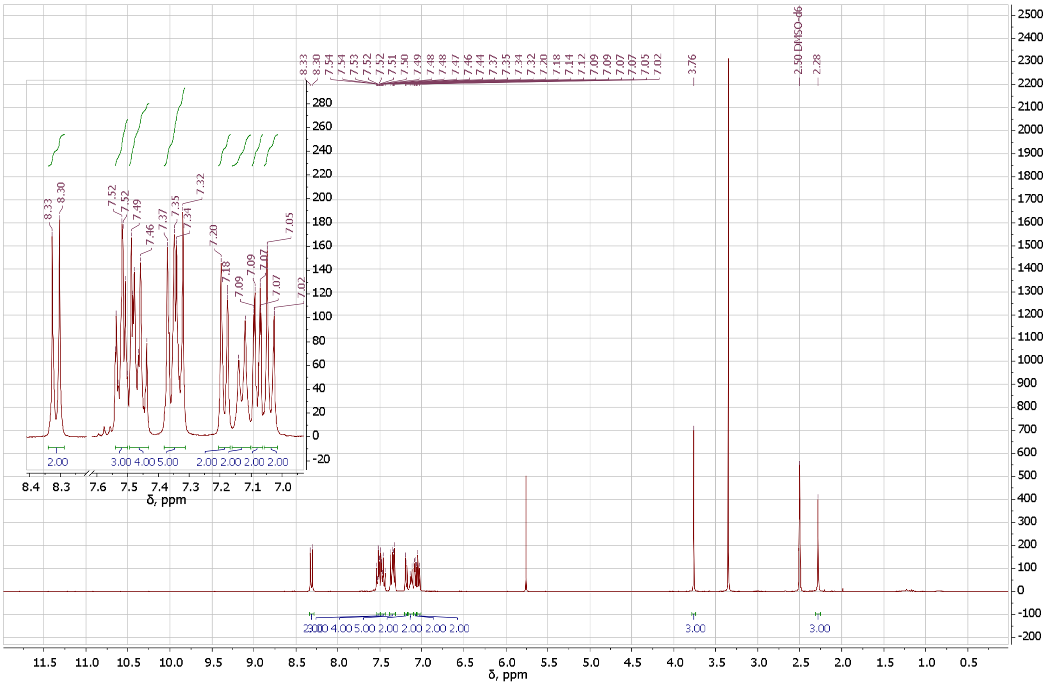


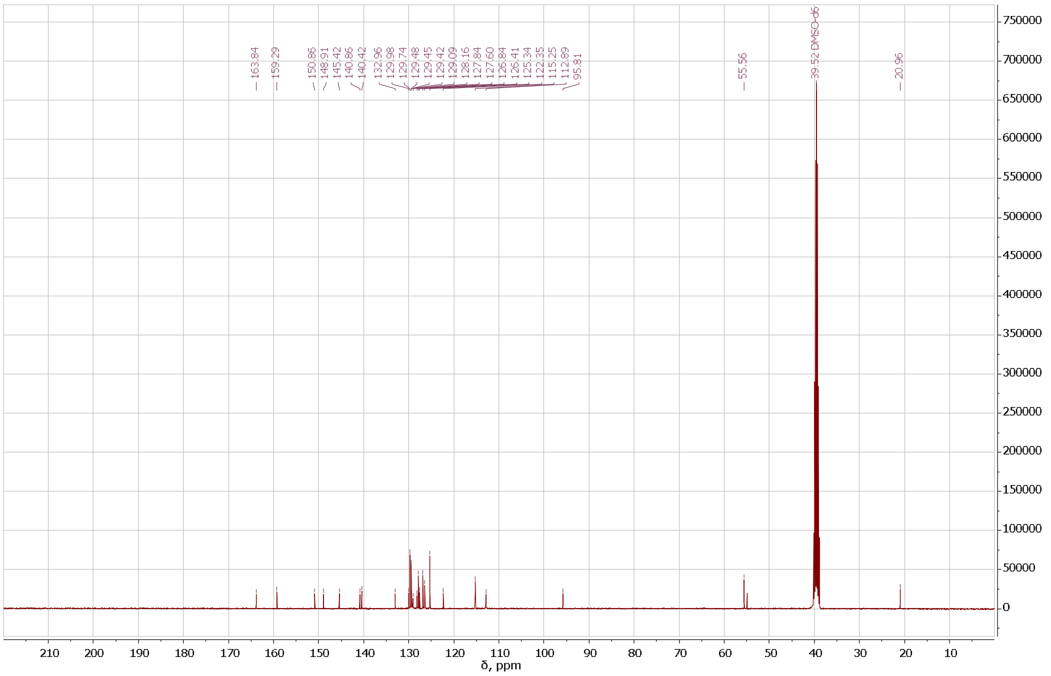


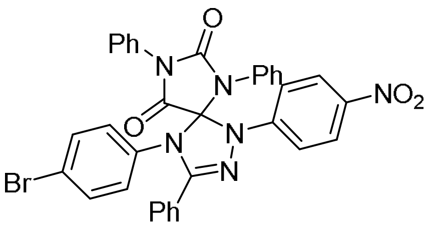


## 4-(4-Bromophenyl)-1-(4-nitrophenyl)-3,6,8-triphenyl-1,2,4,6,8-pentaazaspiro[4.4]non-2-ene-7,9-dione (5h)

$^{1}H$ NMR (400 MHz, $CDCl_3$) δ 8.28 – 8.22 (m, 2H, Ar), 7.50 – 7.46 (m, 3H, Ar), 7.46 – 7.41 (m, 4H, Ar), 7.40 – 7.33 (m, 5H, Ar), 7.30 (m, 5H, Ar), 7.16 – 7.11 (m, 2H, Ar), 6.99 – 6.93 (m,

2H, Ar).

$^{13}C$ NMR (101 MHz, $CDCl_3$) δ 164.3, 151.5, 148.7, 145.7, 141.6, 134.8, 133.3, 133.2, 130.9, 130.2, 129.9, 129.7, 129.5, 129.0, 128.9, 128.2, 128.1, 126.1, 126.0, 125.4, 124.7, 122.8, 113.3, 96.0.

HRMS (ESI): calcd for $C_{34}H_{23}BrN_6O_4$ $(M+Na)^+$ 681.0856, found 681.0853.

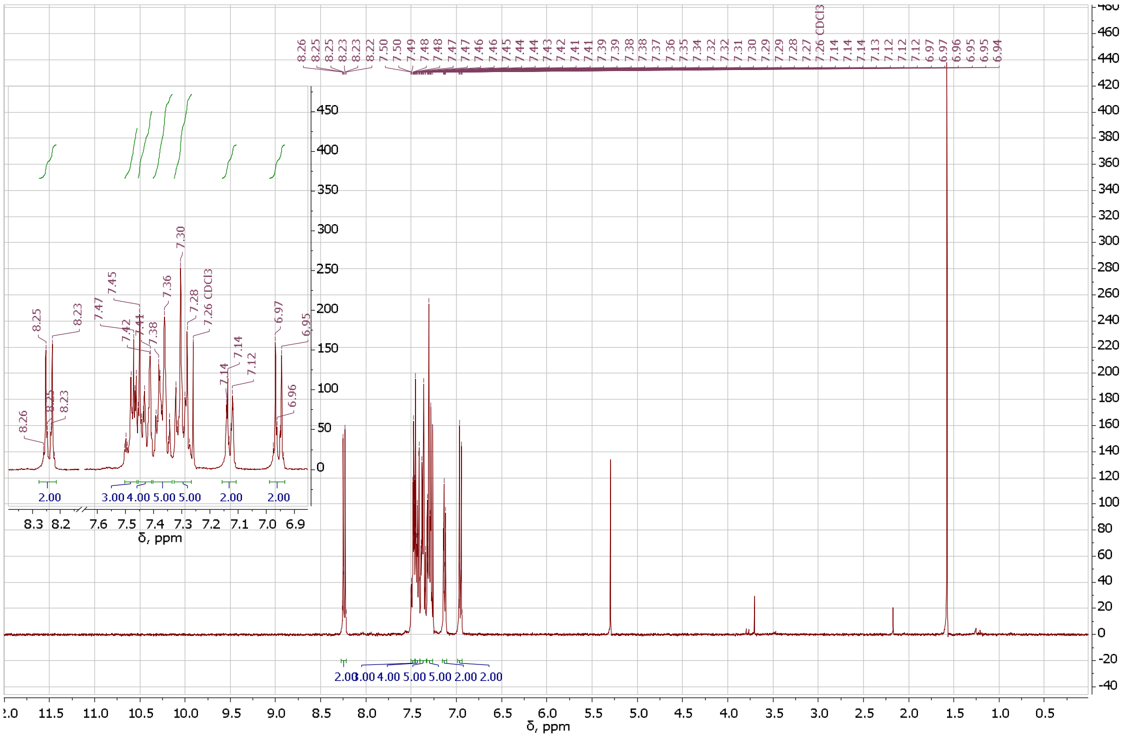


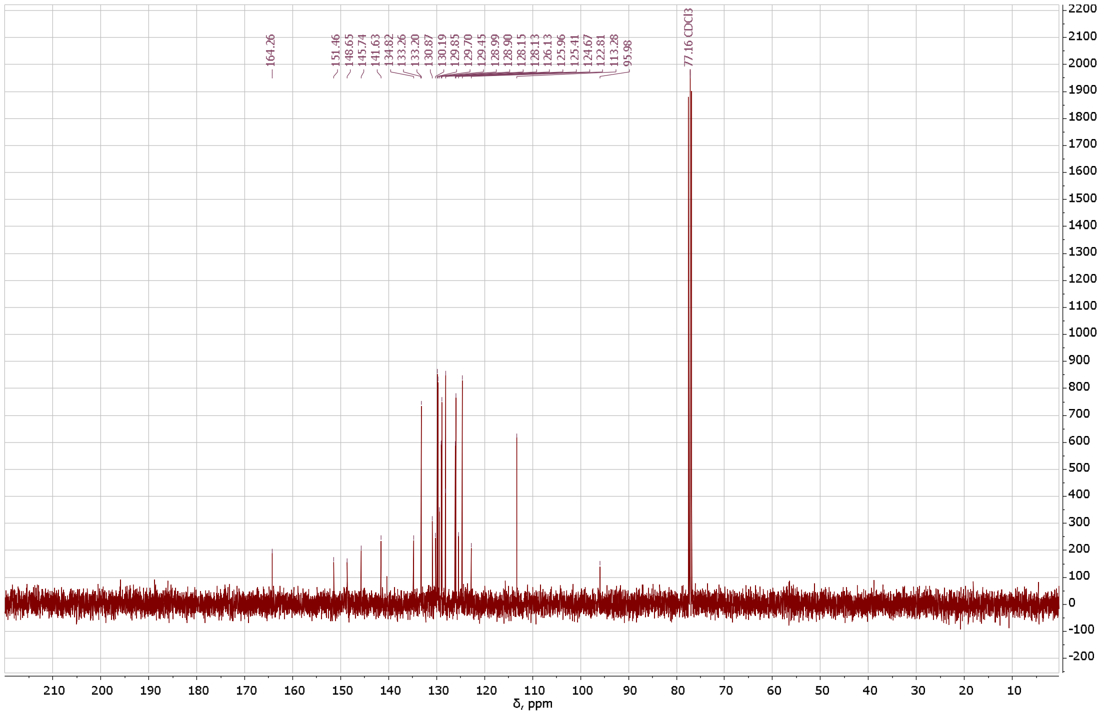


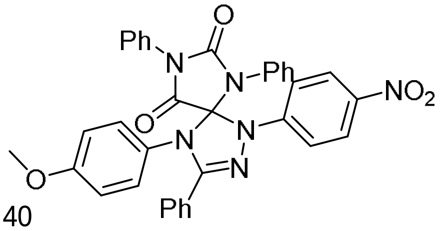


**4-(4-Methoxyphenyl)-1-(4-nitrophenyl)-3,6,8-triphenyl-1,2,4,6,8-pentaazaspiro[4.4]non-2-ene-7,9-dione (5i)**

$^{1}$H NMR (400 MHz, DMSO-d6) δ 8.34 – 8.30 (m, 3H, Ar), 7.54 – 7.51 (m, 3H, Ar), 7.50 – 7.48 (m, 2H, Ar), 7.47 – 7.43 (m, 5H, Ar), 7.40 – 7.36 (m, 4H, Ar), 7.14 (d, *J* = 8.6 Hz, 2H, Ar), 7.10 – 7.07 (m, 2H, Ar), 7.04 (d, *J* = 9.1 Hz, 2H, Ar), 3.76 (s, 3H, $OCH_3$).

$^{13}$C NMR (101 MHz, DMSO-d6) δ 163.9, 159.4, 150.9, 148.9, 145.4, 140.6, 133.0, 130.9, 130.0, 129.8, 129.5, 129.5, 129.1, 128.9, 128.2, 127.9, 127.5, 126.9, 126.4, 125.4, 125.3, 115.3, 113.0, 95.9, 55.6.

HRMS (ESI): calcd for $C_{35}H_{26}N_6O_5$ $(M+H)^+$ 611.2037, found 611.2041.

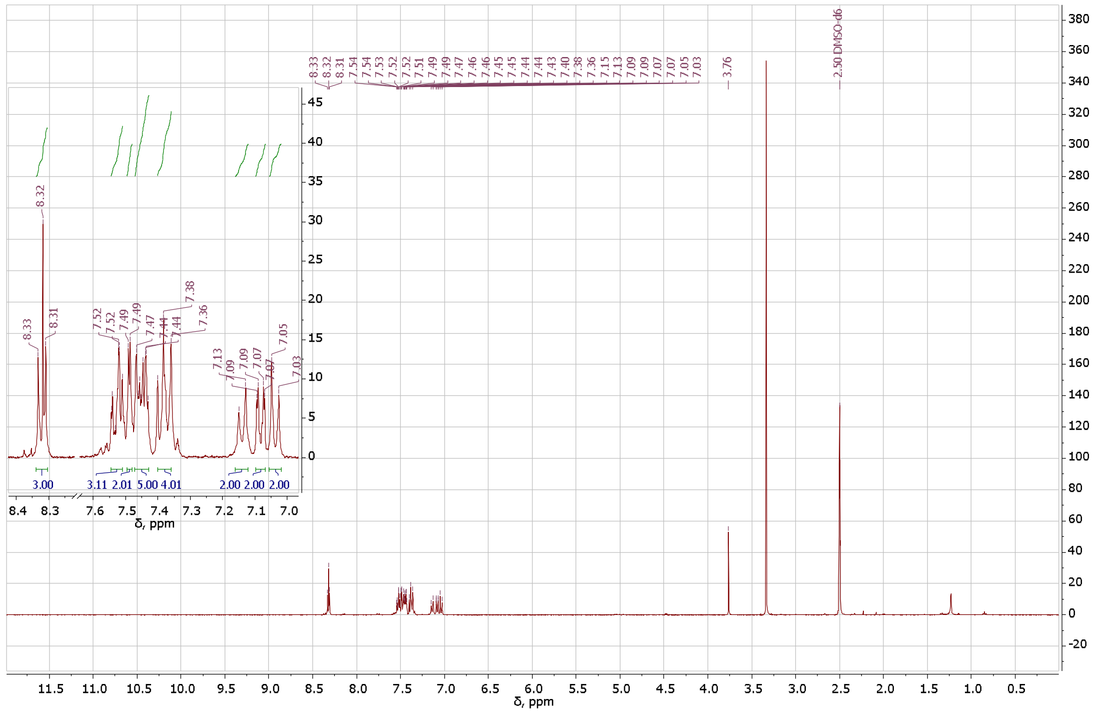


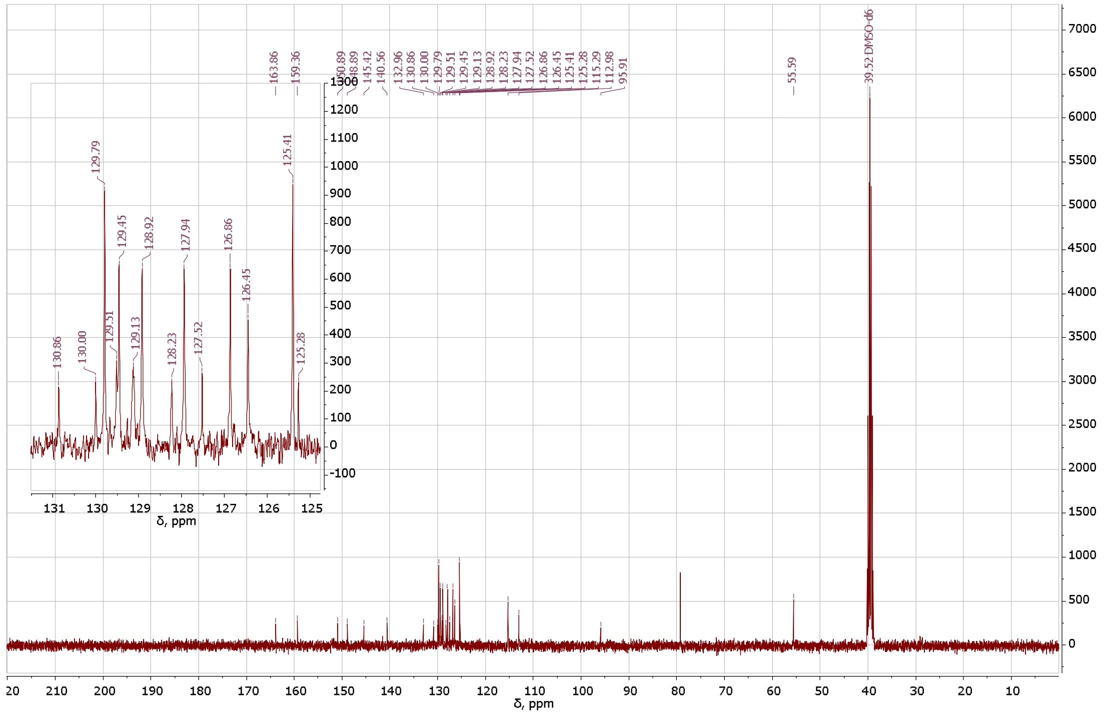


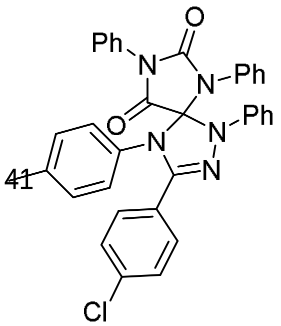


**3-(4-Chlorophenyl)-1,6,8-triphenyl-4-(p-tolyl)-1,2,4,6,8-pentaazaspiro[4.4]non-2-ene-7,9-dione (5j)**

$^1$H NMR (400 MHz, $CDCl_3$) δ 7.53 (d, *J* = 7.7 Hz, 2H, Ar), 7.44 – 7.40 (m, 2H, Ar), 7.40 – 7.34 (m, 4H, Ar), 7.34 – 7.27 (m, 6H, Ar), 7.24 – 7.21 (m, 2H, Ar), 7.13 – 7.09 (m, 4H, Ar), 7.05 – 7.01 (m, 1H, Ar), 6.97 (d, *J* = 8.3 Hz, 2H, Ar), 2.34 (s, 3H, $CH_3$).

$^{13}$C NMR (101 MHz, $CDCl_3$) δ 165.1, 151.9, 146.2, 141.4, 138.6, 135.8, 134.1, 133.5, 130.7, 130.4, 129.7, 129.4, 129.4, 129.1, 128.9, 128.8, 127.4, 127.3, 126.3, 125.2, 124.9, 122.4, 115.3, 97.8, 21.2.

HRMS (ESI): calcd for $C_{35}H_{26}ClN_5O_2$ $(M+H)^+$ 584.1848, found 584.1849.

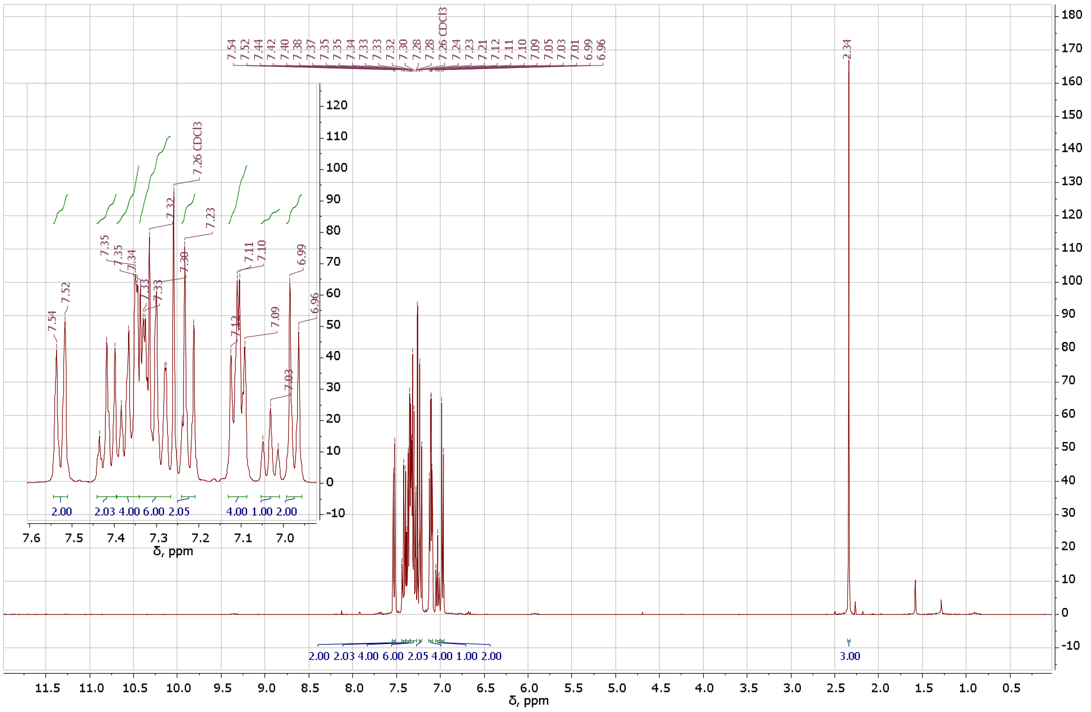


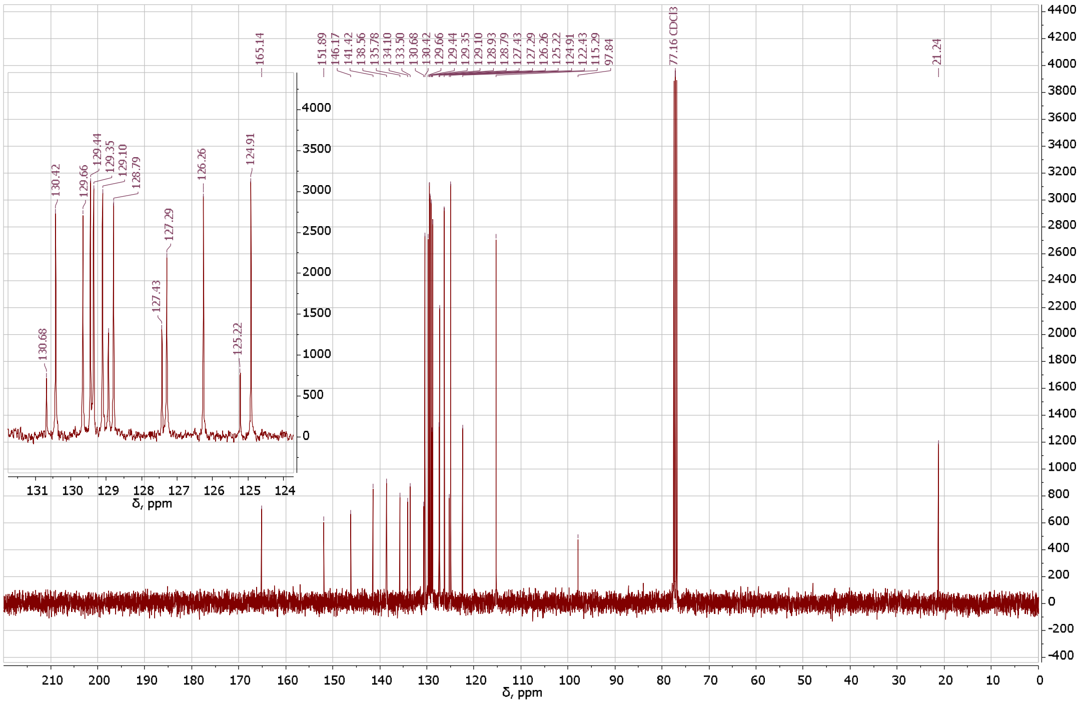


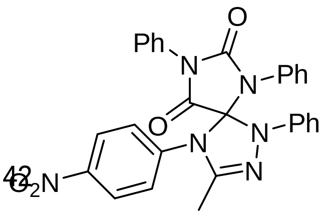


**3-Methyl-4-(4-nitrophenyl)-1,6,8-triphenyl-1,2,4,6,8-pentaazaspiro[4.4]non-2-ene-7,9-dione (5k)**

[1]H NMR (400 MHz, $CDCl_3$) δ 8.27 – 8.22 (m, 2H, Ar), 7.46 – 7.37 (m, 7H, Ar), 7.36 – 7.31 (m, 3H, Ar), 7.23 – 7.19 (m, 4H, Ar), 7.15 – 7.11 (m, 2H, Ar), 7.05 (m, 1H, Ar), 2.03 (s, 3H, $CH_3$).

[13]C NMR (101 MHz, $CDCl_3$) δ 165.5, 151.5, 147.1, 144.1, 141.7, 140.6, 133.9, 130.4, 129.8, 129.6, 129.5, 129.2, 128.1, 127.7, 126.0, 125.3, 124.8, 122.9, 115.9, 96.9, 12.1.

HRMS (ESI): calcd for $C_{29}H_{22}N_6O_4$ $(M+H)^+$ 519.1775, found 519.1772.

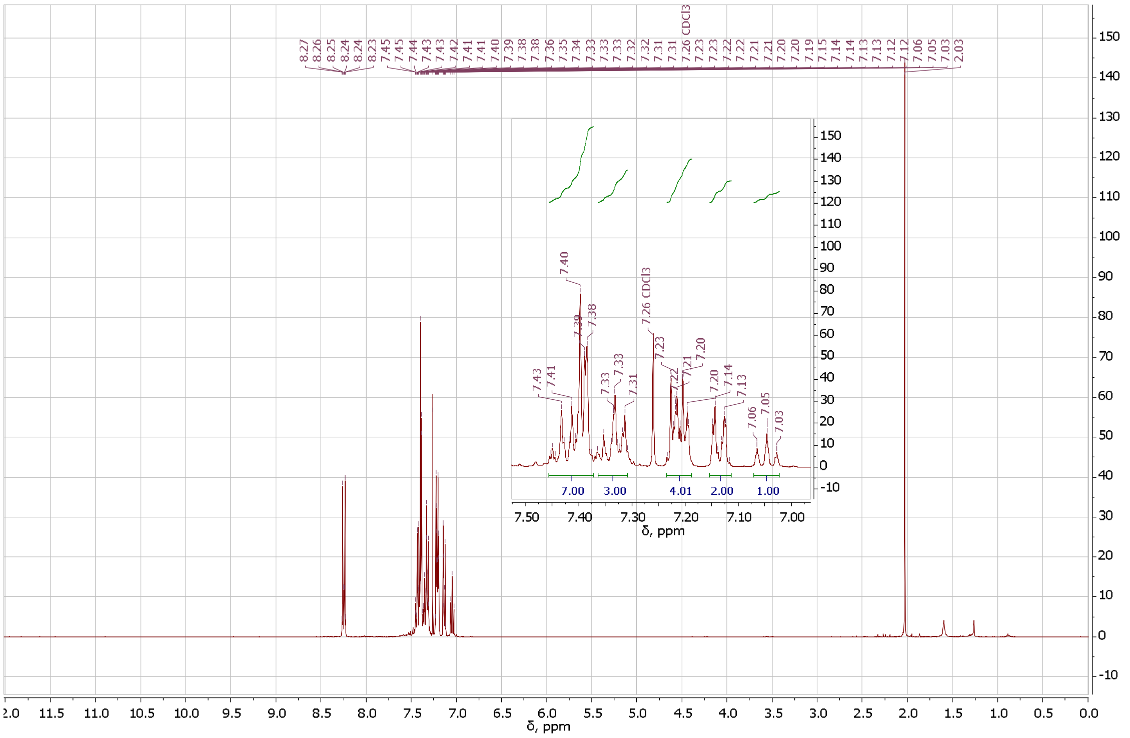


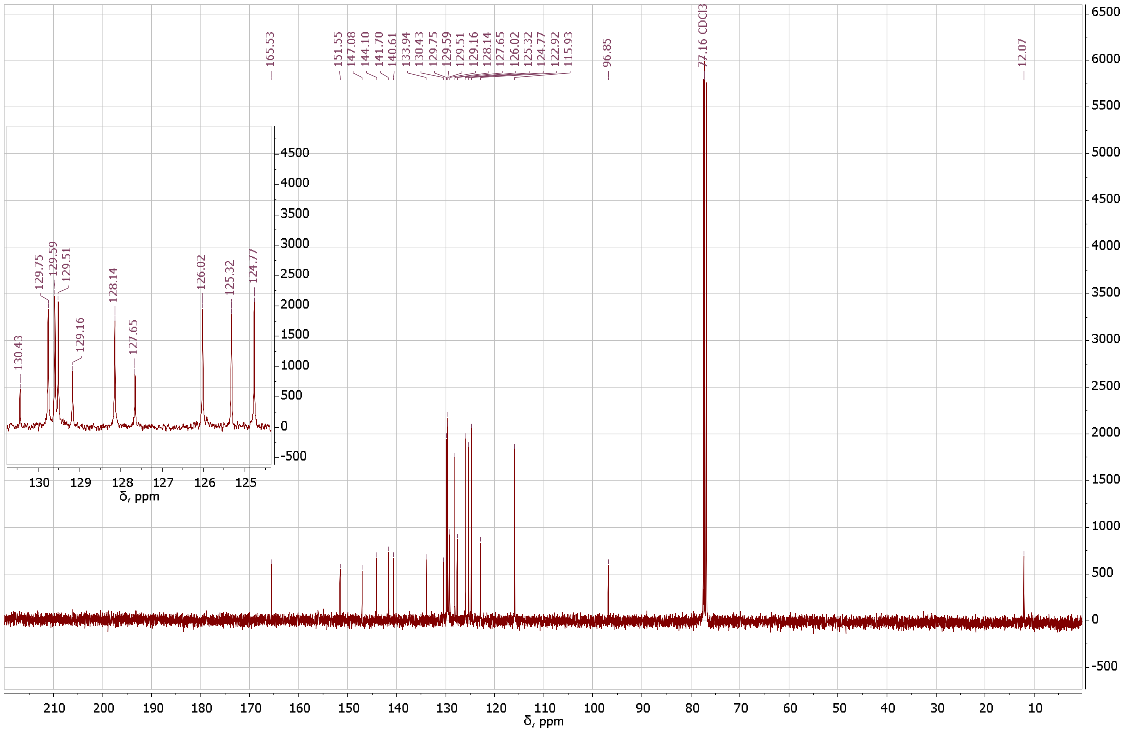


43 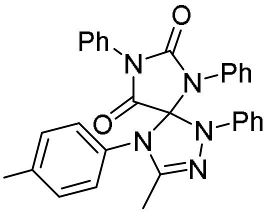


**4-(4-Bromophenyl)-3-methyl-1,6,8-triphenyl-1,2,4,6,8-pentaazaspiro[4.4]non-2-ene-7,9-dione (5l)**

$^{1}H$ NMR (400 MHz, $CDCl_3$) δ 7.54 – 7.50 (m, 2H, Ar), 7.49 – 7.45 (m, 2H, Ar), 7.45 – 7.36 (m, 5H, Ar), 7.35 – 7.28 (m, 3H, Ar), 7.23 – 7.19 (m, 2H, Ar), 7.15 – 7.10 (m, 2H, Ar), 7.03 – 6.98 (m, 1H, Ar), 6.96 – 6.90 (m, 2H, Ar), 1.93 (s, 3H, $CH_3$).

$^{13}C$ NMR (101 MHz, $CDCl_3$) δ 165.9, 151.6, 145.4, 142.0, 134.3, 133.3, 133.2, 130.6, 130.6, 129.7, 129.4, 129.0, 127.2, 126.2, 124.6, 123.4, 122.3, 115.3, 97.1, 11.6.

HRMS (ESI): calcd for $C_{29}H_{22}BrN_5O_2$ $(M+H)^+$ 552.1030, found 552.1023.

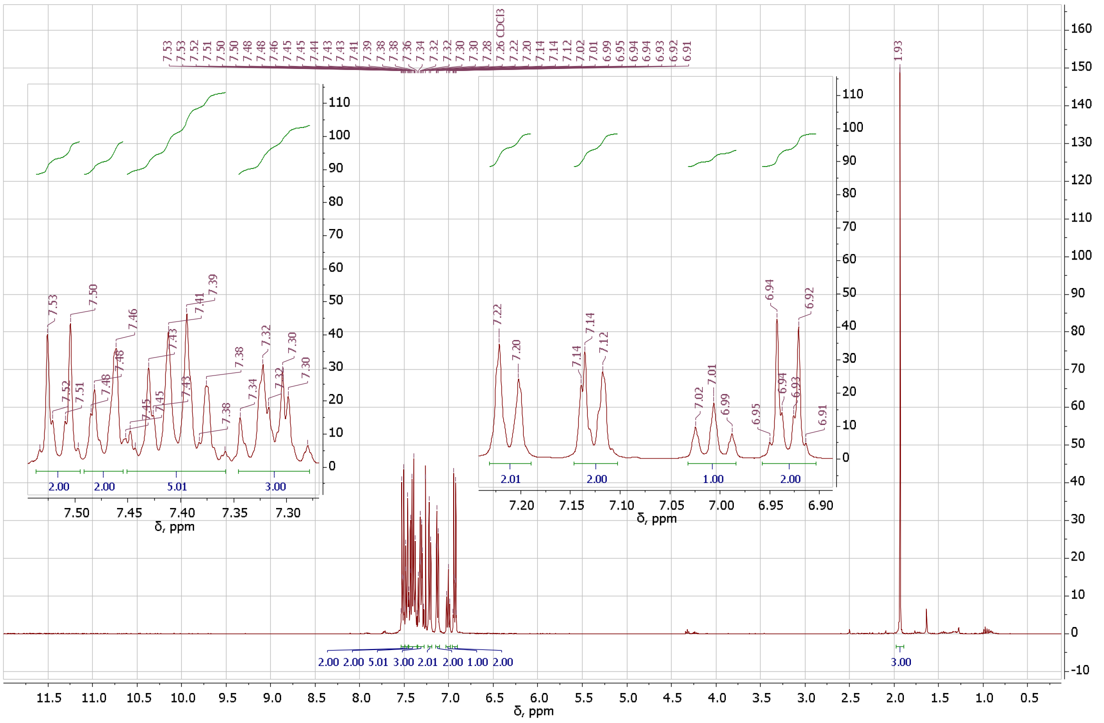


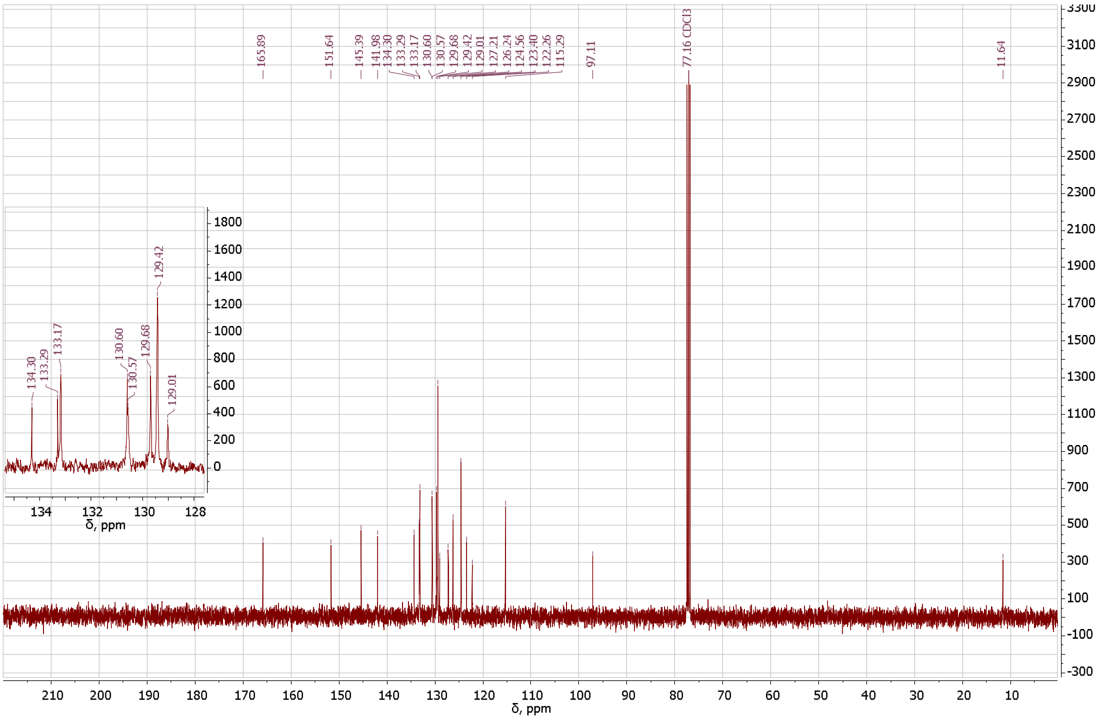

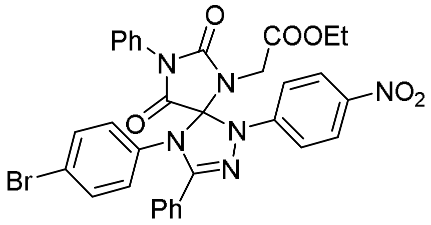


## Ethyl 2-(4-(4-bromophenyl)-1-(4-nitrophenyl)-7,9-dioxo-3,8-diphenyl-1,2,4,6,8-pentaazaspiro[4.4]non-2-en-6-yl)acetate (5m)

$^1$H NMR (400 MHz, $CDCl_3$) δ 8.23 (d, *J* = 9.2 Hz, 2H, Ar), 7.51 – 7.47 (m, 3H, Ar), 7.46 – 7.43 (m, 2H, Ar), 7.42 – 7.40 (m, 2H, Ar), 7.37 – 7.33 (m, 3H, Ar), 7.29 (d, *J* = 9.2 Hz, 2H, Ar), 7.22 (d, *J* = 8.1 Hz, 2H, Ar), 7.09 – 7.02 (m, 2H, Ar), 4.32 (d, *J* = 17.6 Hz, 1H, NC<u>H</u>$_2$), 4.21 – 4.07 (m, 3H, NC<u>H</u>$_2$+COOC<u>H</u>$_2$CH$_3$), 1.19 (t, *J* = 7.1 Hz, 3H, COOCH$_2$C<u>H</u>$_3$).

$^{13}$C NMR (101 MHz, $CDCl_3$) δ 167.4, 164.0, 152.4, 148.8, 146.0, 141.8, 135.6, 133.1, 131.0, 130.1, 129.6, 129.3, 128.9, 128.7, 128.2, 125.9, 125.7, 125.5, 122.5, 113.7, 95.4, 62.3, 41.0, 14.1.

HRMS (ESI): calcd for $C_{32}H_{25}BrN_6O_6$ $(M+Na)^+$ 691.0911, found 691,0913.

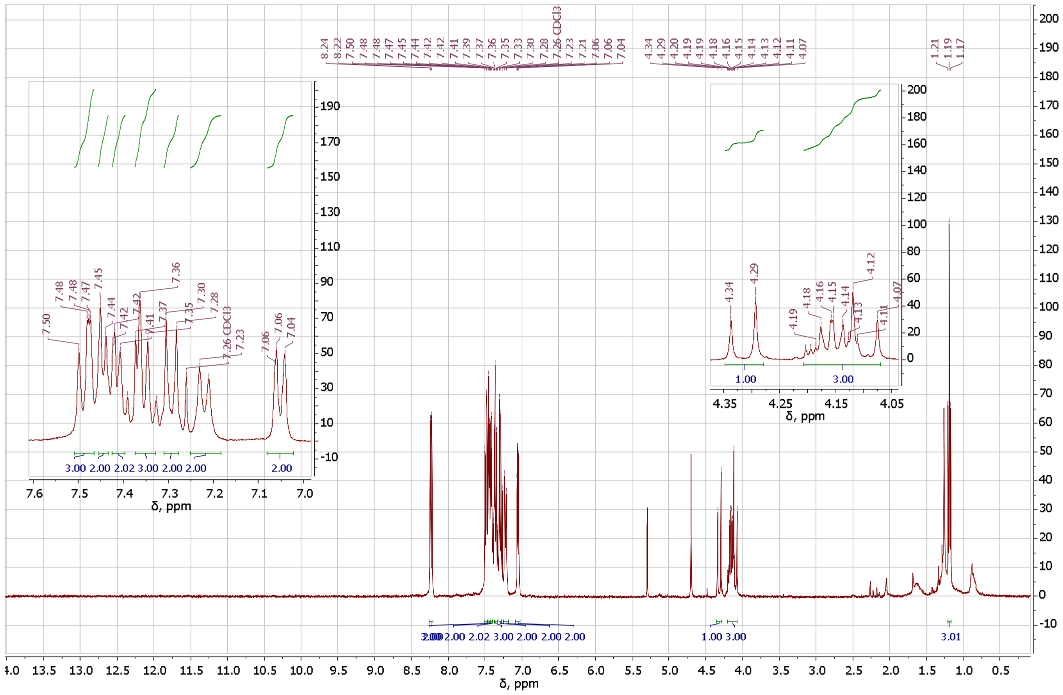

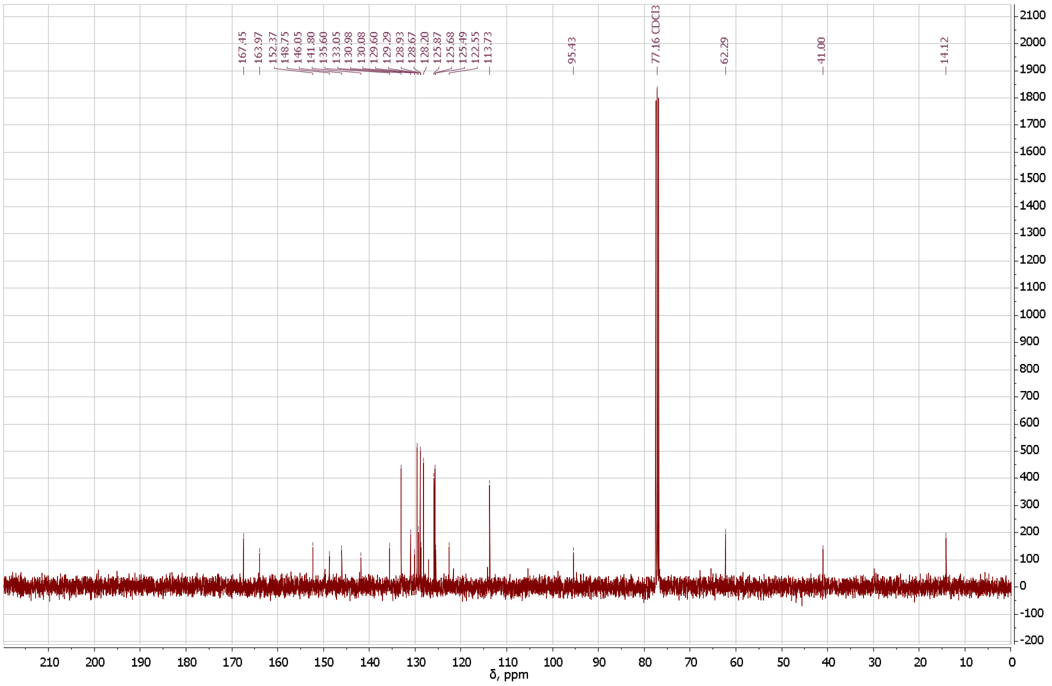


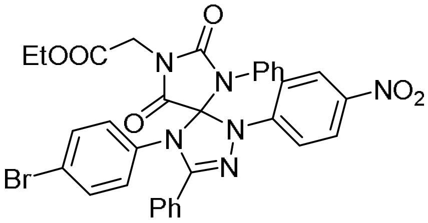


**Ethyl 2-(4-(4-bromophenyl)-1-(4-nitrophenyl)-7,9-dioxo-3,6-diphenyl-1,2,4,6,8-pentaazaspiro[4.4]non-2-en-8-yl)acetate (5n)**

$^1$H NMR (400 MHz, DMSO-d6) δ 8.24 – 8.19 (m, 2H, Ar), 7.60 – 7.56 (m, 2H, Ar), 7.46 – 7.41 (m, 2H, Ar), 7.41 – 7.37 (m, 5H, Ar), 7.37 – 7.31 (m, 3H, Ar), 7.30 – 7.26 (m, 2H, Ar), 7.03 – 6.97 (m, 2H, Ar), 4.56 – 4.45 (m, 2H, $NCH_2$), 4.20 (qd, $J$ = 7.0, 1.4 Hz, 2H, COOC$\underline{H_2}$CH$_3$), 1.21 (t, $J$ = 7.1 Hz, 3H, COOCH$_2$C$\underline{H_3}$).

$^{13}$C NMR (101 MHz, DMSO-d6) δ 167.0, 164.1, 151.1, 148.3, 144.9, 140.7, 134.3, 132.9, 132.3, 130.9, 129.8, 129.0, 128.8, 128.5, 127.9, 126.0, 125.7, 125.0, 121.4, 113.5, 95.3, 61.9, 14.0.

HRMS (ESI): calcd for $C_{32}H_{25}BrN_6O_6$ $(M+Na)^+$ 691.0911, found 691.0913.

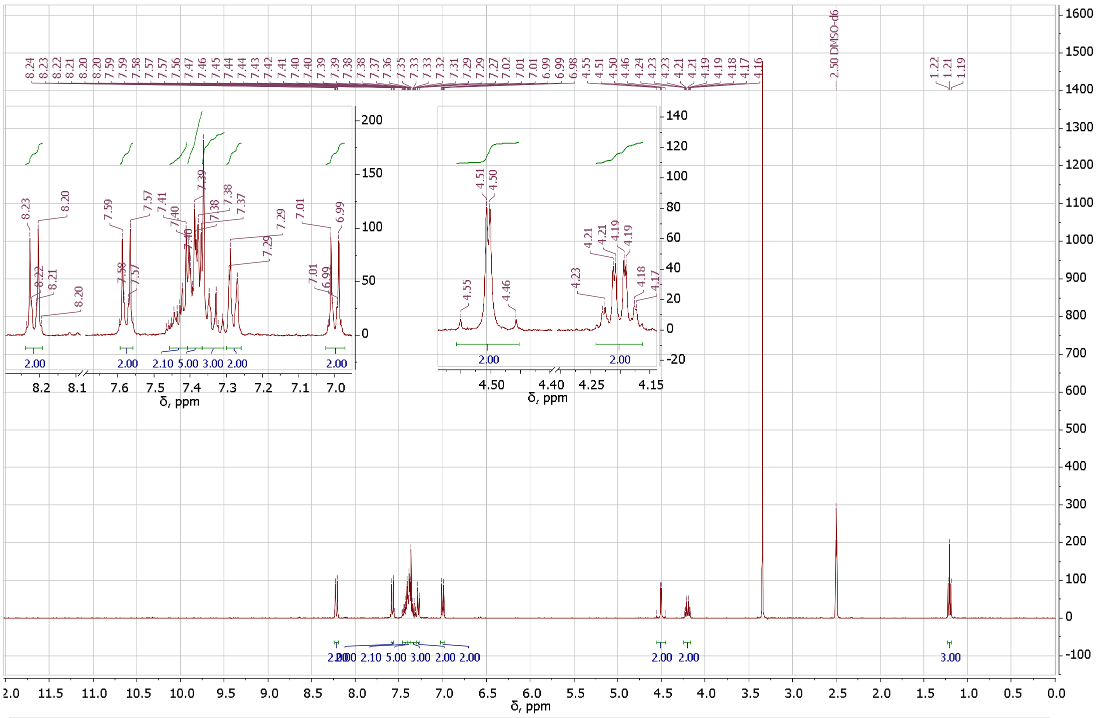


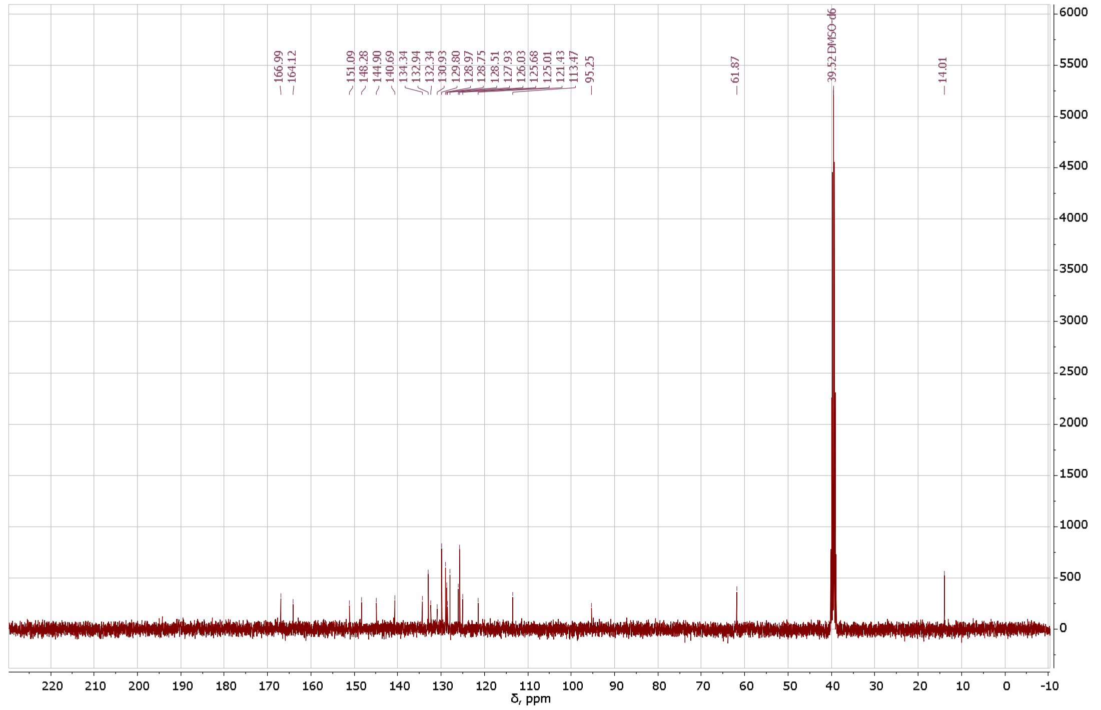


## $^1$H NMR spectral data of compounds 5 and 6

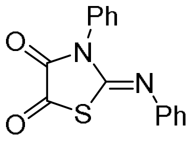


### (Z)-3-Phenyl-2-(phenylimino)thiazolidine-4,5-dione (5)

$^1$H NMR (400 MHz, $CDCl_3$) δ 7.60 – 7.53 (m, 2H, Ar), 7.53 – 7.48 (m, 1H, Ar), 7.48 – 7.42 (m, 2H, Ar), 7.39 – 7.32 (m, 2H, Ar), 7.23 – 7.18 (m, 1H, Ar), 6.94 – 6.89 (m, 2H, Ar).

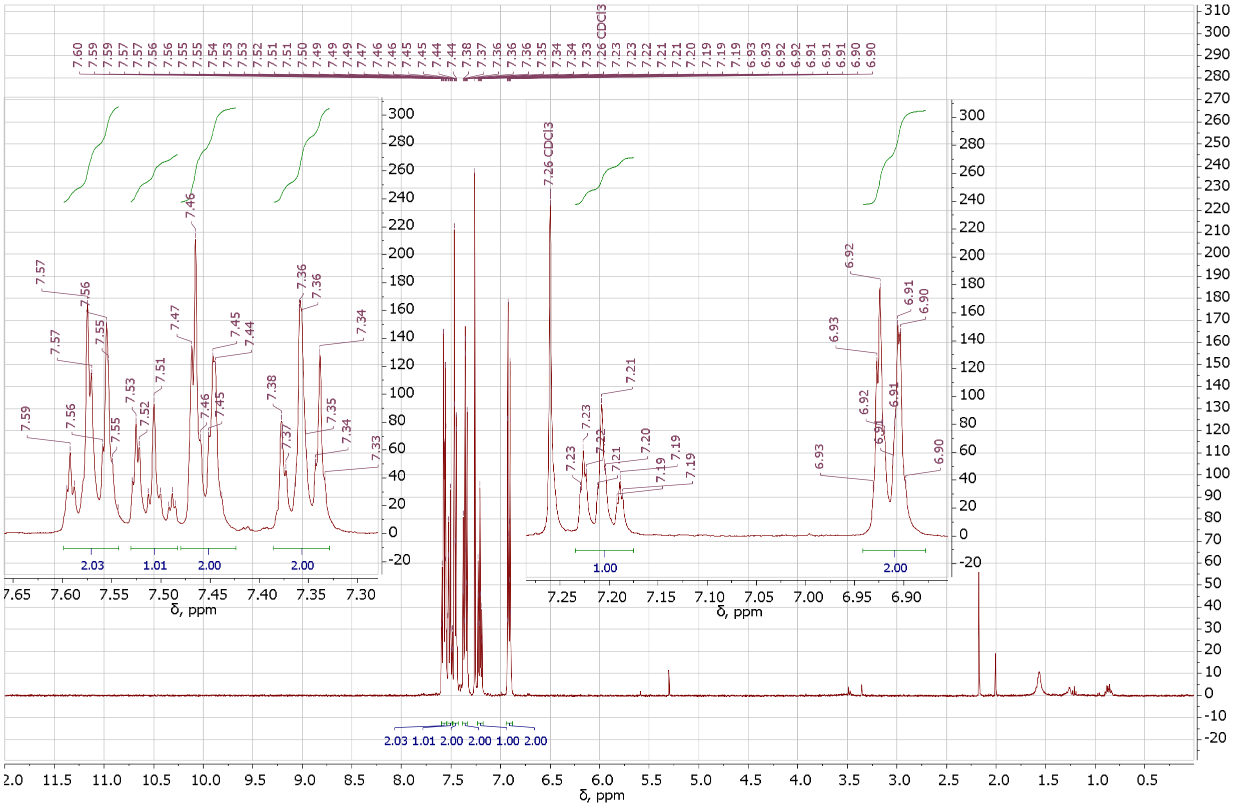

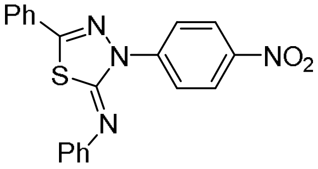


**(Z)-3-(4-nitrophenyl)-N,5-diphenyl-1,3,4-thiadiazol-2(3H)-imine (6)**

$^1$H NMR (400 MHz, $CDCl_3$) δ 8.55 – 8.50 (m, 2H, Ar), 8.35 – 8.30 (m, 2H, Ar), 7.76 – 7.70 (m, 2H, Ar), 7.50 – 7.40 (m, 5H, Ar), 7.23 – 7.17 (m, 1H, Ar), 7.12 – 7.07 (m, 2H, Ar).

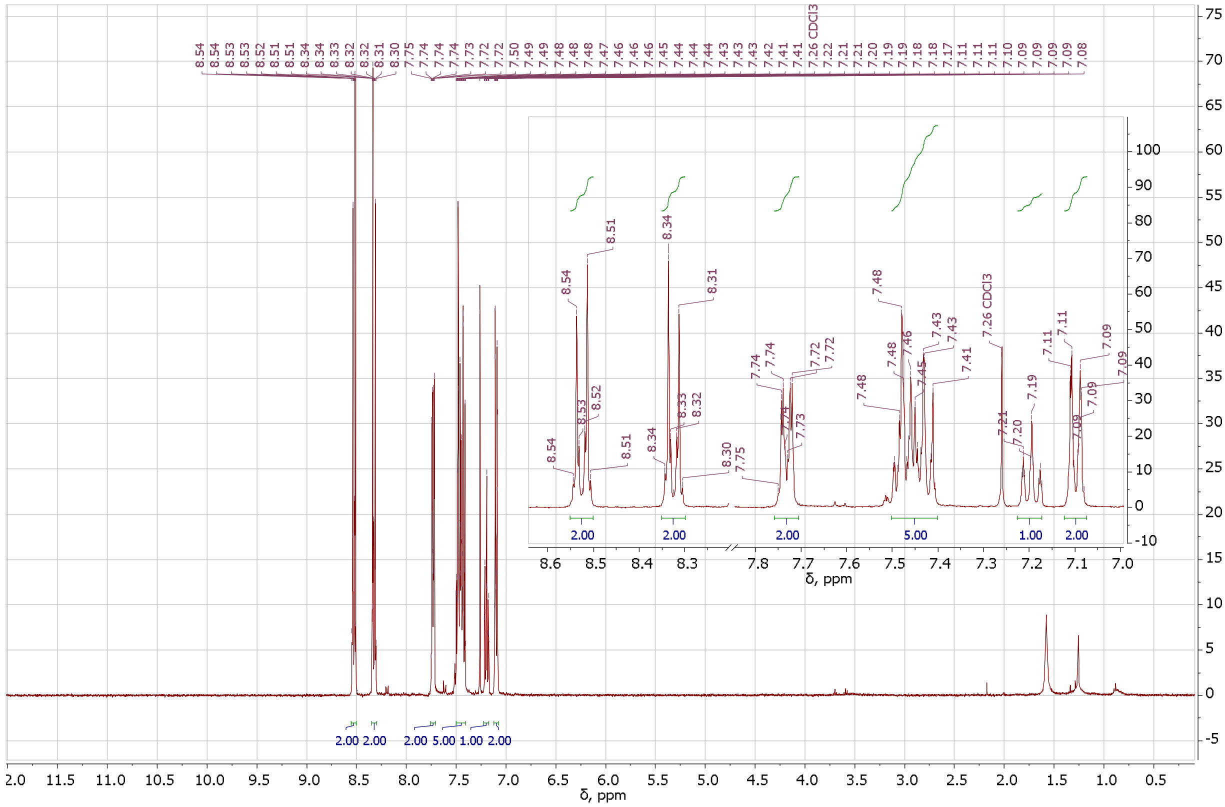

## IR spectral data of 1c, 2i, 2j and 5

### 1,3-Diphenyl-2-thioxoimidazolidine-4,5-dione (1c)

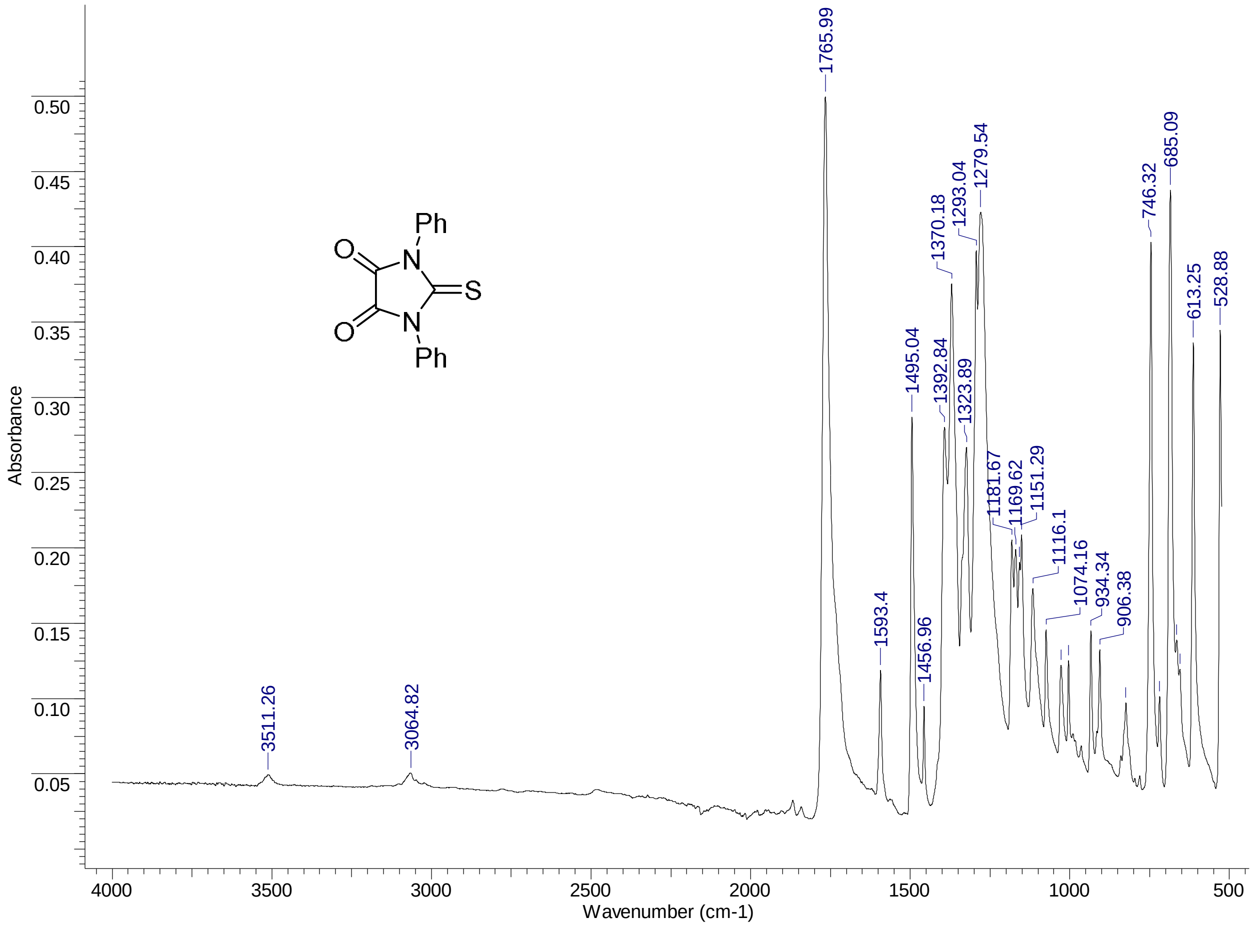

# 5-((4-Methoxyphenyl)imino)-1,3-diphenyl-2-thioxoimidazolidin-4-one (2i)

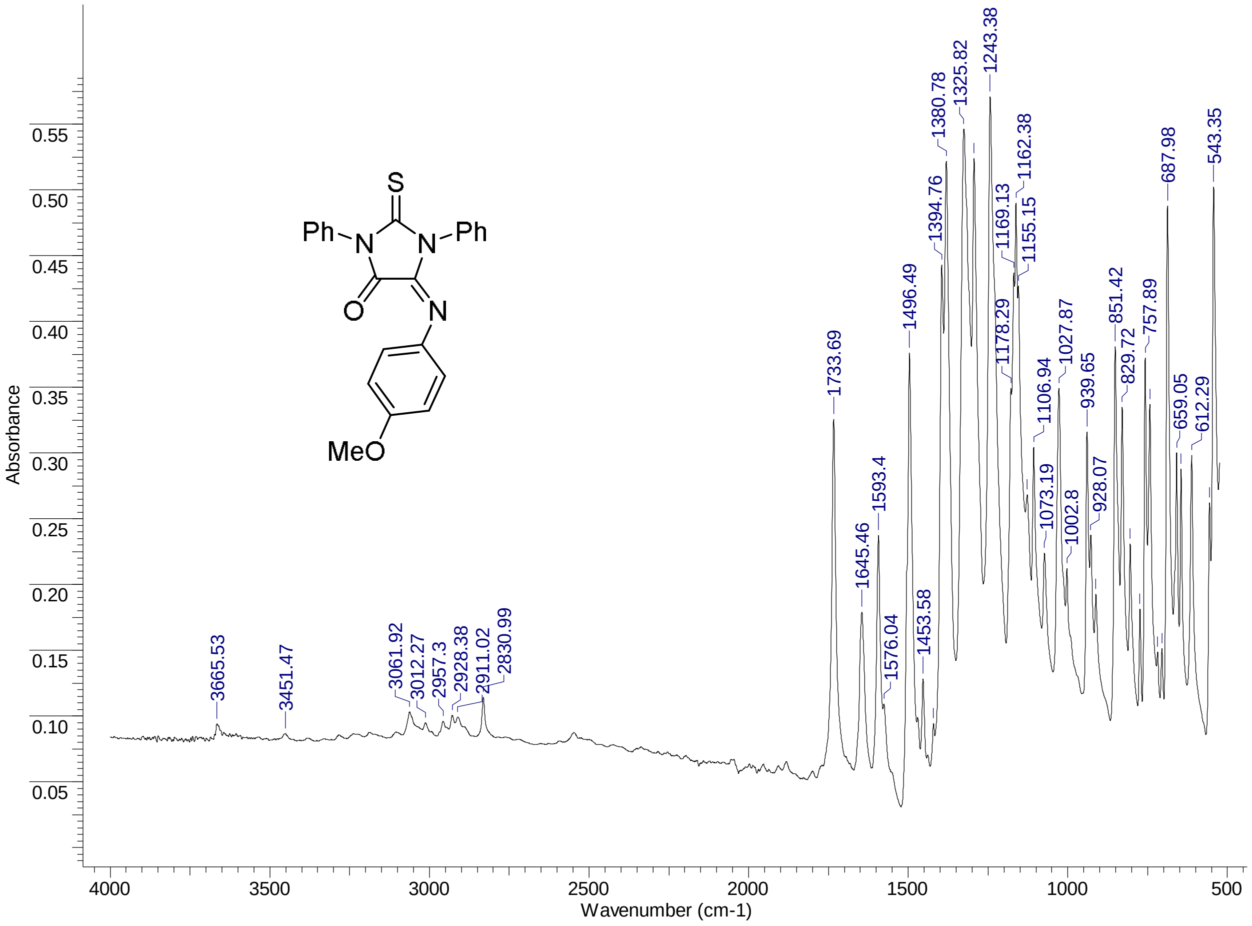

# 5-((4-Nitrophenyl)imino)-1,3-diphenyl-2-thioxoimidazolidin-4-one (2j)

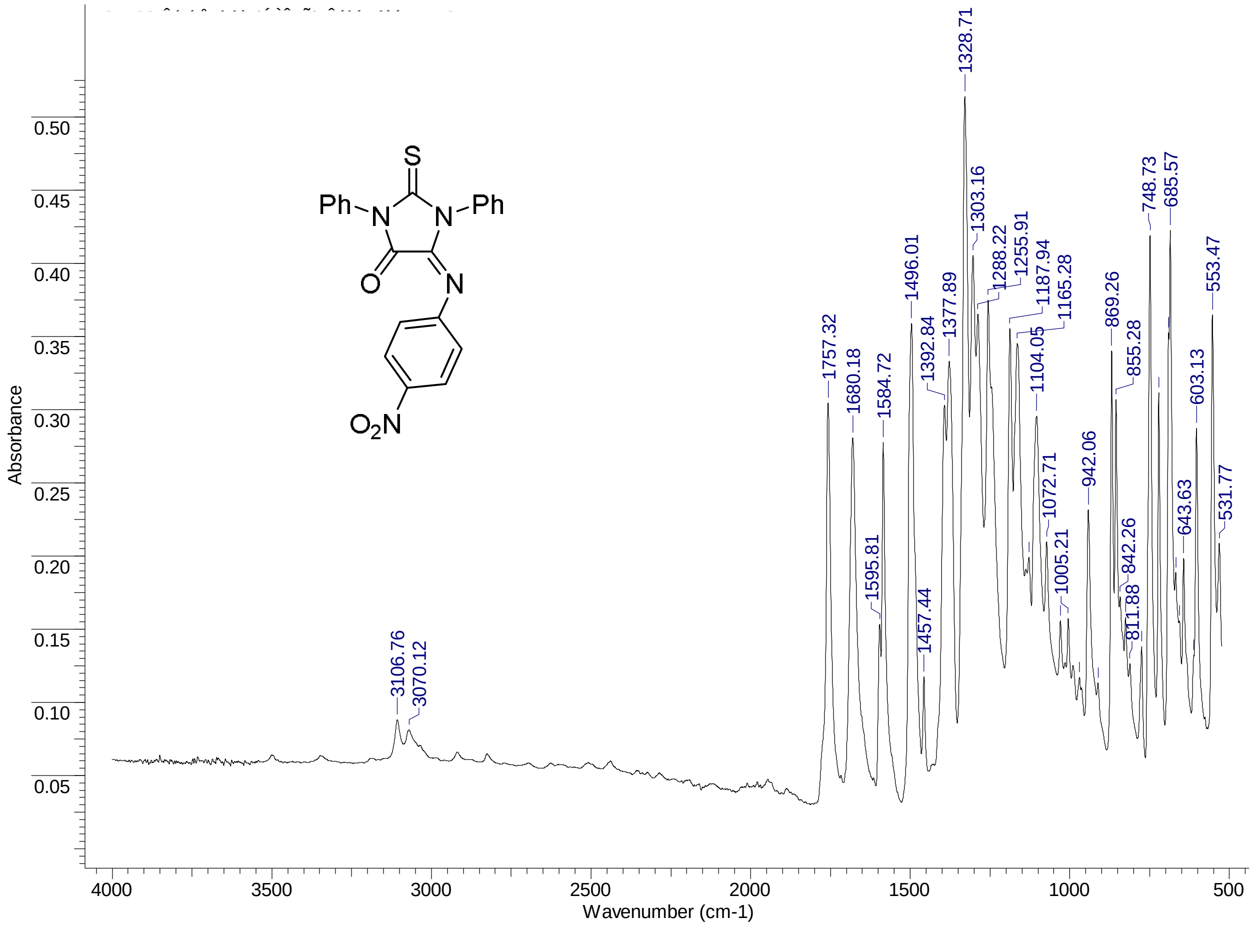

# 3-Phenyl-2-(phenylimino)thiazolidine-4,5-dione (5)

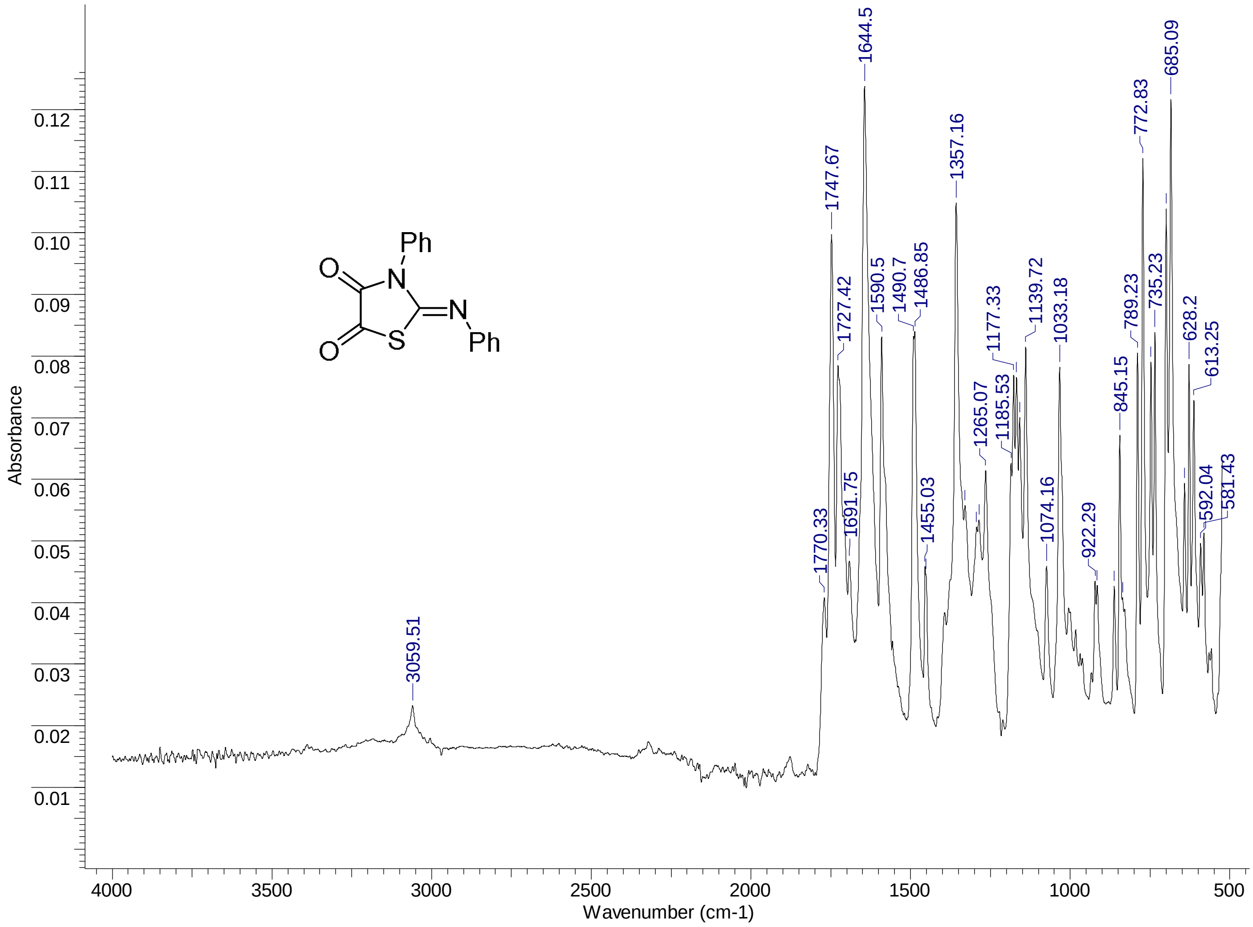

**Crystallographic data of 5c, 5g and 5i**

Temperature: 295(2) K

Wavelength: 1.54186 Å

Refinement method: Full-matrix least-squares on $F^2$

| # | **5c** | **5g** | **5i** |
|---|---|---|---|
| Empirical formula | $C_{35}H_{25}BrN_6O_4$ | $C_{36}H_{28}N_6O_5$ | $C_{36}H_{27}Cl_3N_6O_5$ |
| Formula weight | 673.52 | 624.64 | 729.98 |
| Crystal system | Triclinic | Monoclinic | Monoclinic |
| Space group | P -1 | P 21/n | P 21/n |
| a, Å | 10.7603(3) | 16.2888(6) | 11.3803(5) |
| b, Å | 11.0967(3) | 12.3447(3) | 20.6633(8) |
| c, Å | 15.1828(4) | 16.4893(6) | 15.5580(8) |
| $\alpha$, ° | 93.286(3) | 90 | 90 |
| $\beta$, ° | 94.472(3) | 104.864(3) | 108.201(3) |
| $\gamma$, ° | 98.835(3) | 90 | 90 |
| $V$, Å$^3$ | 1781.50(9) | 3204.72(19) | 3475.5(3) |
| Z | 2 | 4 | 4 |
| $d_{calc}$, Mg/m$^3$ | 1.256 | 1.295 | 1.395 |
| Absorption coefficient, mm$^{-1}$ | 1.933 | 0.726 | 2.823 |
| F(000) | 688 | 1304 | 1504 |
| Theta range for data collection, ° | 2.927 to 67.410 | 4.425 to 67.584 | 4.246 to 67.625 |
| Index ranges | -12<=h<=3<br>-13<=k<=13<br>-17<=l<=18 | -19<=h<=19<br>-10<=k<=14<br>-15<=l<=19 | -8<=h<=13<br>-24<=k<=23<br>-18<=l<=18 |
| Reflections collected | 7618 | 23681 | 25638 |
| Independent reflections [$R_{int}$] | 5047 [0.0689] | 5748 [0.0915] | 6196 [0.0802] |
| Completeness to $\theta$ = 66.511°, % | 78.8 | 99.2 | 98.6 |
| Data / restraints / parameters | 5047 / 0 / 417 | 5748 / 0 / 427 | 6196 / 0 / 452 |
| Goodness-of-fit on $F^2$ | 0.907 | 0.716 | 0.894 |
| Final R indices [$I > 2\sigma(I)$] | R1 = 0.0444<br>wR2 = 0.1144 | R1 = 0.0403<br>wR2 = 0.0729 | R1 = 0.0799<br>wR2 = 0.2182 |
| R indices (all data) | R1 = 0.0646<br>wR2 = 0.1212 | R1 = 0.1220<br>wR2 = 0.0872 | R1 = 0.1639<br>wR2 = 0.2552 |
| Extinction coefficient | 0.0019(3) | 0.00097(6) | 0.0013(3) |
| Largest diff. peak and hole, e. Å$^{-3}$ | 0.514 and -0.619 | 0.147 and -0.174 | 0.708 and -0.599 |
